# Gibberellin-mediated internode elongation for grafting and early flowering induction in Brassicaceae crops

**DOI:** 10.1270/jsbbs.25036

**Published:** 2026-01-16

**Authors:** Makishi Hara, Satomi Shimizu, Masahide Yoshizumi, Kaisei Miyaki, Yuka Machi, Shunsuke Miyashima, Keitaro Tanoi, Natsuko I. Kobayashi, Tenta Segawa, Hiroki Takagi

**Affiliations:** 1 Ishikawa Prefectural University, 1-308 Suematsu, Nonoichi, Ishikawa 921-8836, Japan; 2 The University of Tokyo, 1-1-1 Yayoi, Bunkyo-ku, Tokyo113-8657, Japan; 3 Fukushima Institute for Research, Education and Innovation (F-REI), 6-1 Yazawa-machi, Gongendo, Namie Town, Fukushima 979-1521, Japan

**Keywords:** grafting, vernalization, Brassicaceae, *FT*, *SPL3*, gibberellin

## Abstract

In Brassicaceae crops such as cabbage and turnip, which are harvested for their vegetative organs, floral induction can reduce the eating quality of these organs. Therefore, developing late-flowering varieties that are insensitive to vernalization is a key breeding objective. However, a major challenge in breeding these cultivars lies in the contradiction between the late-flowering trait and the need to induce flowering for crossing. To address this, we developed a flowering induction technique using grafting, in which gibberellin-induced elongated internodes are used as scions and grafted onto the flower stalk of *Brassica rapa* cv. ‘CHOY SUM EX CHINA 3’, which constitutively expresses *FLOWERING LOCUS T* (*FT*), as the rootstock. This method is applicable even to rosette-type Brassicaceae plants with short internodes. Histological and radioisotope analyses confirmed the reconnection of vascular bundles and the functionality of component transport across the graft junction. Additionally, gibberellin treatment was found to promote flowering in the presence of FT, most likely through an activation of *SQUAMOSA PROMOTER BINDING PROTEIN-LIKE 3*, a gene known to promote floral meristem development. This rapid flowering system offers a practical strategy for accelerating the breeding of late-flowering Brassicaceae crops.

## Introduction

The Brassicaceae family, also known as Cruciferae, comprises approximately 3,709 species across 338 genera (Warwick *et al.* 2006). This family includes widely cultivated and consumed vegetables such as turnip (*Brassica rapa*), cabbage (*B. oleracea*), and radish (*Raphanus sativus*), which are primarily valued for their vegetative organs. It also encompasses crops like broccoli (*B. oleracea*), mustard greens (*B. juncea*), and oilseed rape (*B. napus*), which are cultivated for their reproductive organs, including flowers and seeds.

In Brassicaceae crops, regulation of flowering time is critical. In vegetables such as cabbage and turnip, which are harvested for their vegetative organs, the initiation of floral bud differentiation can significantly reduce crop quality. Conversely, flowering is essential for crops cultivated for their reproductive structures, such as broccoli and oilseed rape. Extensive research into the molecular mechanisms controlling flowering in this family has focused on *Arabidopsis thaliana*, a model species in Brassicaceae (Komeda 2004). In many Brassicaceae plants, flowering requires a prolonged period of cold exposure, known as vernalization (Madrid *et al.* 2021). This process is primarily regulated by the interaction between *FLOWERING LOCUS C* (*FLC*) and *FLOWERING LOCUS T* (*FT*) genes. Prior to vernalization, *FLC* represses *FT* expression. Following cold exposure, *FLC* expression is epigenetically silenced, enabling *FT* to activate downstream floral integrator genes, which in turn promote floral meristem development (Kim and Sung 2014).

In Brassicaceae vegetables cultivated for their vegetative organs, breeding efforts have focused on delaying flowering to prevent yield and quality losses caused by floral development (Jung and Müller 2009). Consequently, research has targeted *FLC* alleles that are insensitive to cold temperatures to develop late-flowering cultivars across several Brassicaceae species, including *B. rapa*, *B. oleracea*, and *R. sativus* (Kitamoto *et al.* 2014, Li *et al.* 2022, Wang *et al.* 2018). In *B. rapa*, Kitamoto *et al.* (2014) identified two *FLC* alleles requiring extended cold exposure for suppression due to the insertion of a transposable element that makes them insensitive to epigenetic silencing. These alleles have been successfully incorporated into breeding programs, resulting in the development of the late-flowering Chinese cabbage cultivar ‘ITOSAI No. 1’ (Kitamoto *et al.* 2023). Furthermore, Shea *et al.* (2018) successfully developed a late-flowering cultivar by introgressing an *FLC* allele from *B. oleracea* into *B. rapa* via homoeologous recombination between the two genomes. This finding highlights the potential to utilize effective *FLC* alleles from one species in breeding closely related species. Consequently, late-flowering cultivars in the Brassicaceae plants could be systematically developed through the strategic application of diverse *FLC* alleles.

However, breeding late-flowering cultivars presents a challenge due to the lengthy process involved. Each generation requires an extended period of low-temperature treatment to induce flowering, which significantly prolongs the breeding timeline. To address this issue, Motoki *et al.* (2019) developed a grafting technique to induce flowering within a shorter time frame. In this approach, flowering was induced in an extremely late-flowering *B. oleracea* cultivar by grafting its stem onto the flower stem of *R. sativus*, allowing FT protein from *R. sativus* to move to the apical meristem of *B. oleracea*. While this strategy successfully induced flowering in some Brassicaceae plants with sufficiently elongated internodes for grafting, its applicability to rosette-type species with very short internodes—such as turnip, Chinese cabbage, and radish—has been challenging.

Gibberellin (GA) is known to promote internode elongation in plants (McKim 2020). Based on this property, our previous study applied gibberellic acid (GA_3_) treatment to seedlings of *R. sativus* to overcome the limitation of their naturally short internodes (Nishikawa *et al.* 2023). This treatment successfully promoted internode elongation, making them suitable for use as scions in grafting. The elongated internodes were then successfully grafted onto the flower stalk of *B. rapa* cv. ‘CHOY’, which expresses *FT* without requiring vernalization. As a result, the grafted scion flowered, facilitated by the transfer of FT from the ‘CHOY’ rootstock to the scion.

However, the optimal conditions for GA_3_ treatment and grafting techniques have not yet been fully established. For successful application to the grafting process, GA_3_ treatment must produce a compact scion that allows for easier handling, minimizes stress on the rootstock, and reduces the risk of transpiration loss. Additionally, the grafting procedure described in a previous study was highly complex and required considerable space, making it inefficient and resulting in a low success rate. Therefore, it is essential to determine the optimal GA_3_ treatment conditions for preparing suitable scions and to develop more efficient grafting techniques.

Furthermore, GA is known to promote not only internode elongation but also flower bud differentiation under short-day conditions in Arabidopsis via the GA signaling pathway (Wilson *et al.* 1992). In Arabidopsis, this pathway enables the induction of floral buds by GA_3_ treatment alone, but whether this pathway functions similarly in other Brassicaceae species remains unclear. Recently, in Pakchoi (*B. rapa* cv. ‘Caixin’), knockout mutants of DELLA family genes—negative regulators of GA signaling—exhibited an early flowering phenotype, suggesting that GA treatment may promote flowering (Wang *et al.* 2023). Additionally, Song *et al.* (2019) and Jung *et al.* (2020) reported that GA treatment following vernalization promoted early flowering in lines of *B. rapa* and *R. sativus*, respectively, both of which have weak vernalization requirements for floral induction. However, GA treatment alone does not consistently induce flowering across all Brassicaceae species, and the underlying mechanisms for this variability remain unclear. Therefore, it is important to evaluate how GA treatment for internode elongation in scions affects floral transition in Brassicaceae species.

In this study, to extend the applicability of grafting-based floral induction to a wider range of Brassicaceae species with rosette-type growth habits, we developed an integrated approach that combines GA_3_-induced internode elongation with a simplified grafting technique. For this object, we investigated the optimal GA_3_ treatment conditions to elongate internodes and evaluated the grafting success using these elongated scions. The graft compatibility and molecular transport in the grafted plant were confirmed by histological analyses and radioisotope analyses. Furthermore, to understand the effects of GA_3_ treatment on the flowering pathway, we compared gene expression patterns between GA_3_-treated and untreated samples using RNA-seq analysis.

## Materials and Methods

### Plant materials

*B. rapa* cv. ‘CHOY SUM EX CHINA 3’ and ‘Kohiki’ were obtained from the National Agriculture and Food Research Organization (NARO) Genebank of Japan (https://www.gene.affrc.go.jp/index_en.php). The NARO Genebank accession numbers of these cultivars are provided in Supplemental Table 1. *B. rapa* cv. ‘Tsukena no 2’ and ‘Yellow Sarson’ were obtained from Kobe University. *R. sativus* cv. ‘Benikururi’ was purchased from Matsunaga seed in Japan.

### GA_3_ Treatment Conditions

For GA_3_ treatment, Gibberellin A_3_ (Tokyo Chemical Industry, Japan) was applied to the shoot apex by droplet application. The GA_3_ solution was prepared by mixing with 0.1% of the spreading agent ‘Approach BI’ (MARUWA BIOCHEMICAL, Japan) to enhance penetration and adhesion.

### Grafting methods

Scions were prepared from plants grown in 3 × 3 cm cell trays. These plants were treated with 0.5 mM GA_3_ twice, applied at 2 days intervals after the emergence of two fully expanded true leaves. Rootstocks were also grown in 3 × 3 cm cell trays for approximately 15 days before being transplanted into 9 cm pots. After bolting (approximately four weeks of cultivation in 9 cm pots), the flower stalks of the rootstocks were used for grafting. The soil used in this cultivation was Tanemaki Baido (Takii Seed, Japan), with a balanced N:P:K ratio of 380:290:340.

Grafting was carried out using Method 2, as shown in Supplemental Fig. 14 of Motoki *et al.* (2022). Scions were cut into a V-shape and inserted into a vertical slit made at the center of the horizontally cut flower stalk of the rootstock (Supplemental Fig. 1). The junction of grafted plants was secured with ‘Tsugiki Clip No. 2’ (Sakata Seed, Japan), then covered with a clear plastic bag to maintain humidity, followed by a black plastic bag to suppress transpiration (designated as day 0 of curing). On day 3 of curing, the black plastic bag was removed. From day 4 to day 7, the clear plastic bag was gradually opened by cutting small holes that were progressively enlarged, allowing the plants to acclimate to ambient conditions. In this study, grafted ‘Benikururi’ plants were cultivated from October 18 to December 27, 2024, in a greenhouse at Ishikawa Prefectural University. Supplemental lighting was provided by MF250:L/BU-SC-2 (Panasonic, Japan) from 7:00 to 19:00 to complement the weak natural sunlight during the winter season in the Hokuriku region and to maintain plant growth.

### Histological analysis

For paraffin section, the plant tissues were fixed in FAA solution (45% ethanol, 45% distilled water, 5% formalin, 5% acetic acid) for 24 h, then dehydrated through a graded series of butanol (10%, 20%, 35%, 55%, 75%, and 100%, 24 h each). Samples were infiltrated with melted paraffin at 60°C for 3 days, cooled to room temperature, and embedded after 24 h. Sections (20 μm) were cut with a microtome and mounted on glass slides. Deparaffinization was performed by immersing slides in 100% xylene for 5 min. Sections were stained with 0.05% toluidine blue (WALDECK) for 3 min, rinsed in tap water for 5 min, and mounted with EUKITT (O. KINDLER) under cover slips.

Confocal laser scanning microscopy was performed using Leica Stellaris 5 with a solid-state red laser (638 nm) to excite toluidine blue. A series of 2D confocal images was recorded at 5-μm intervals, and a projected image was subsequently generated using ImageJ software.

### Radioisotope analysis

Carbon-14-labeled carbon dioxide (^14^CO_2_) was applied to the grafted plants by enclosing the rootstock in a plastic bag filled with ^14^CO_2_ gas which was generated by mixing NaH^14^CO_3_ (5 MBq) solution with lactic acid. After 4 hours, the scion and the junction of the grafted plant, both was not in the bag, were collected and contacted to an imaging plate (GE Healthcare UK, Amersham, UK) for autoradiography. Before the contact, the thick stem including the junction was split in half lengthwise so that the inside of the stem could be observed. A part of the rootstock stem, which was in the bag and in direct exposure to the ^14^C gas, was also sampled for reference. The imaging plate was scanned using an Amersham Typhoon scanner (Amersham, UK).

Phosphorus-32 (^32^P-phosphate) was applied to the plant via the cut surface of the hypocotyl by dipping it into 15 mL of 1/2 Hoagland solution containing inorganic phosphate (Pi; 0.1 μM) and ^32^P-labeled phosphate (5 MBq). The ^32^P distribution was sequentially obtained using a nondestructive imaging system namely Real-time Radioisotope Imaging System (RRIS) (Sugita *et al.* 2016). Radioactivity images were captured every 30 minutes to monitor the progression of ^32^P distribution.

### RNA-seq analysis

For RNA extraction, shoot apex region containing one unexpanded leaf (<3 cm) from three individual plants were pooled to create a single biological replicate (Supplemental Fig. 2). Total RNA was extracted using the RNeasy Plant Mini Kit (QIAGEN), followed by genomic DNA removal with TURBO DNase (Thermo Fisher Scientific). RNA libraries were prepared using the NEBNext Ultra II RNA Library Prep Kit for Illumina (New England Biolabs) and sequenced as paired-end reads on the Illumina NovaSeq 6000 platform. The RNA-seq reads were aligned to the *B. rapa* reference genome ‘Chiifu V4.1’ (http://brassicadb.cn/#/) using HISAT2 version 2.1.0 (Kim *et al.* 2019). SAM and BAM file conversions were performed using SAMtools version 1.6 (Li *et al.* 2009). Read counts for each gene, based on the GTF annotation of the Chiifu V4.1 reference genome, were obtained using FeatureCounts version 2.0.0 (Liao *et al.* 2014). The resulting count data were normalized to TPM (Transcripts Per Million) values using a custom script (Wagner *et al.* 2012). To identify differentially expressed genes (DEGs) in response to GA_3_ treatment, we performed two-tailed t-tests between GA_3_-treated and untreated samples at the same time point, using three biological replicates. Sequence reads from the RNA-seq analysis have been deposited in a publicly available database at DDBJ, and accession numbers are provided in Supplemental Table 2.

## Results

### Optimizing conditions of GA_3_ treatment for internode elongation

**Fig. 1.**
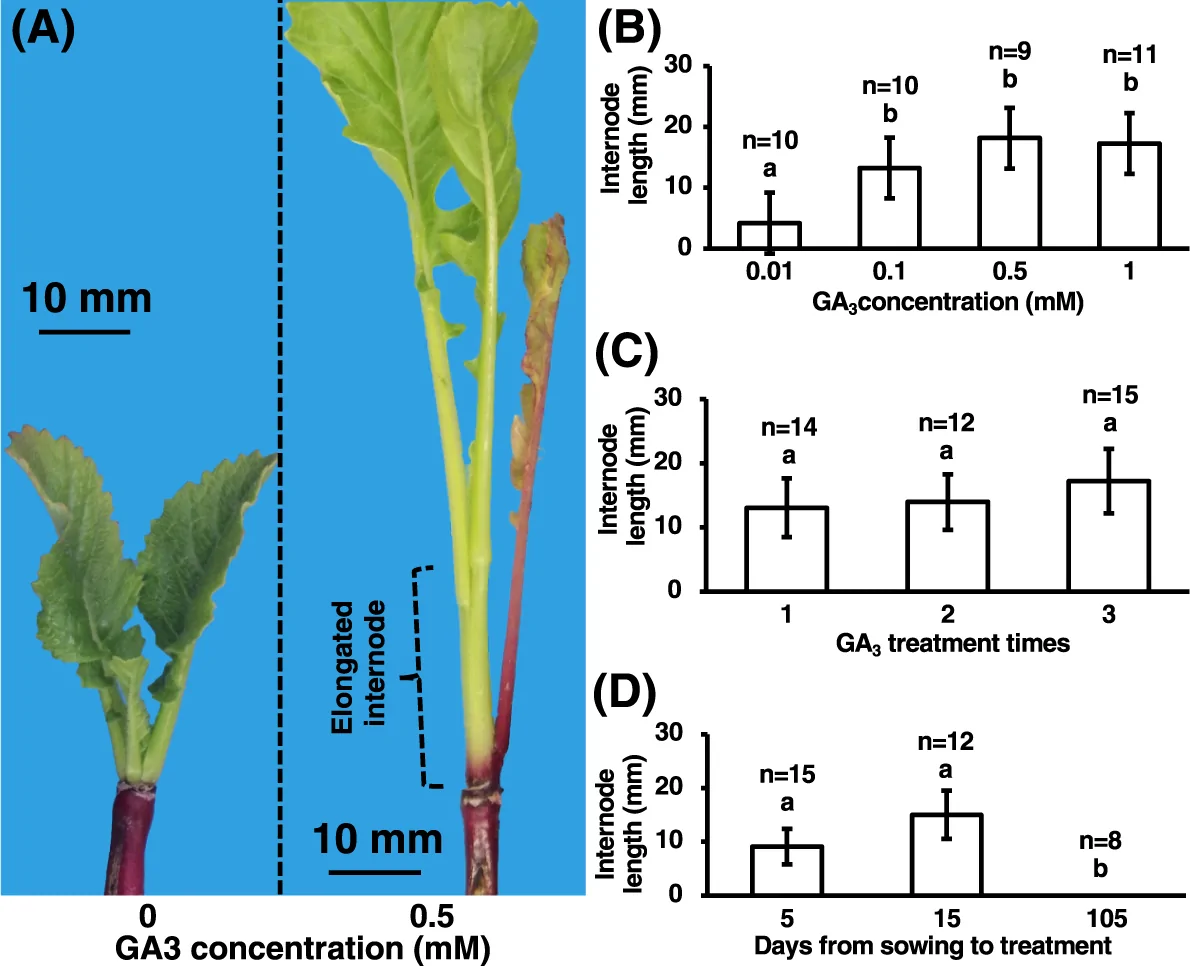
Effect of GA_3_ treatment on internode elongation in ‘Benikururi’. (A) Internode elongation after 4 weeks of 0.5 mM GA_3_ treatment. (B–D) Length of the elongated internode under different GA_3_ treatment conditions: (B) GA_3_ concentration. Each GA_3_ concentration was applied three times, every other day, at the stage when two main leaves had emerged. No internode elongation was observed in untreated plants (0 mM GA_3_). (C) Number of treatments. A 0.5 mM GA_3_ solution was applied at each treatment when two main leaves had emerged. Treatments were conducted two or three times, every other day. (D) Days after sowing at the time of treatment. The shoot apex was treated with 0.5 mM GA_3_ twice. *n* = number of treated individuals. Bars represent mean values of the total length of elongated internodes that emerged after GA_3_ treatment, and error bars indicate standard deviations. Different letters on the bars denote statistically significant differences (p < 0.05) according to Tukey’s test.

To determine an effective and stable conditions for using GA_3_ to promote internode elongation, we tested various treatment protocols on rosette-leaved *R. sativus* cv. ‘Benikururi’ (hereafter referred to as ‘Benikururi’). Initially, GA_3_ solutions at various concentrations (0.01, 0.1, 0.5, and 1 mM) were applied to the shoot apex three times in total, at two-day intervals, starting 15 days after sowing. After one month, untreated control plants showed no internode elongation, whereas all GA_3_-treated plants exhibited visible internode elongation, with elongation increasing with concentration up to 0.5 mM (Fig. 1a, 1b, Supplemental Fig. 3a). There was no statistically significant difference in elongation between the 0.5 mM and 1 mM treatments, indicating that 0.5 mM is sufficient to induce elongation. At this concentration, elongation was predominantly observed in the second internode, located between the second and third true leaves, rather than in the third internode (Supplemental Fig. 3b). This observation suggests that the response to GA_3_ may depend on the developmental stage of each internode at the time of application. Furthermore, the presence of a spreader was essential for achieving elongation (Supplemental Fig. 3c). Based on these results, we conclude that 0.5 mM GA_3_ with a surfactant is the optimal concentration for use in subsequent experiments.

To further evaluate the effect of application frequency, we examined the impact of applying 0.5 mM GA_3_ once, twice, or three times at two-day intervals. Although no significant differences in internode elongation were observed among the different frequencies (Fig. 1c), these results helped confirm that repeated applications do not provide additional benefit beyond a single treatment. Nonetheless, because a single application may occasionally be ineffective due to insufficient contact with the shoot apex, we adopted a two-time treatment protocol in the following experiments to ensure consistency.

Furthermore, we investigated the optimal timing for initiating GA_3_ treatment by applying it to seedlings at 5, 15, and 105 days after sowing (Fig. 1d, Supplemental Fig. 4). At 5 days, no main leaves had emerged, while by 15 days, most plants had developed two main leaves. By 105 days, plants exhibited more than five leaves and signs of hypocotyl enlargement. Internode elongation was observed in plants treated at both 5 and 15 days, with the 15-day-old group exhibiting slightly greater elongation. In contrast, no elongation was observed in the 105-day-old plants, likely because the rosette leaf arrangement at this stage physically blocked GA_3_ absorption at the shoot apex. Depletion of nutrients supplied by the fertilizer contained in the soil, which remained effective for only 30–40 days, may also have affected internode elongation. Based on these findings, 15 days after sowing was selected as the optimal timing for GA_3_ application in subsequent experiments.

Consequently, in this study, 15-day-old plants were treated twice with 0.5 mM GA_3_ to induce internode elongation suitable for grafting.

### Comparison of grafting using different scion organs

**Fig. 2.**
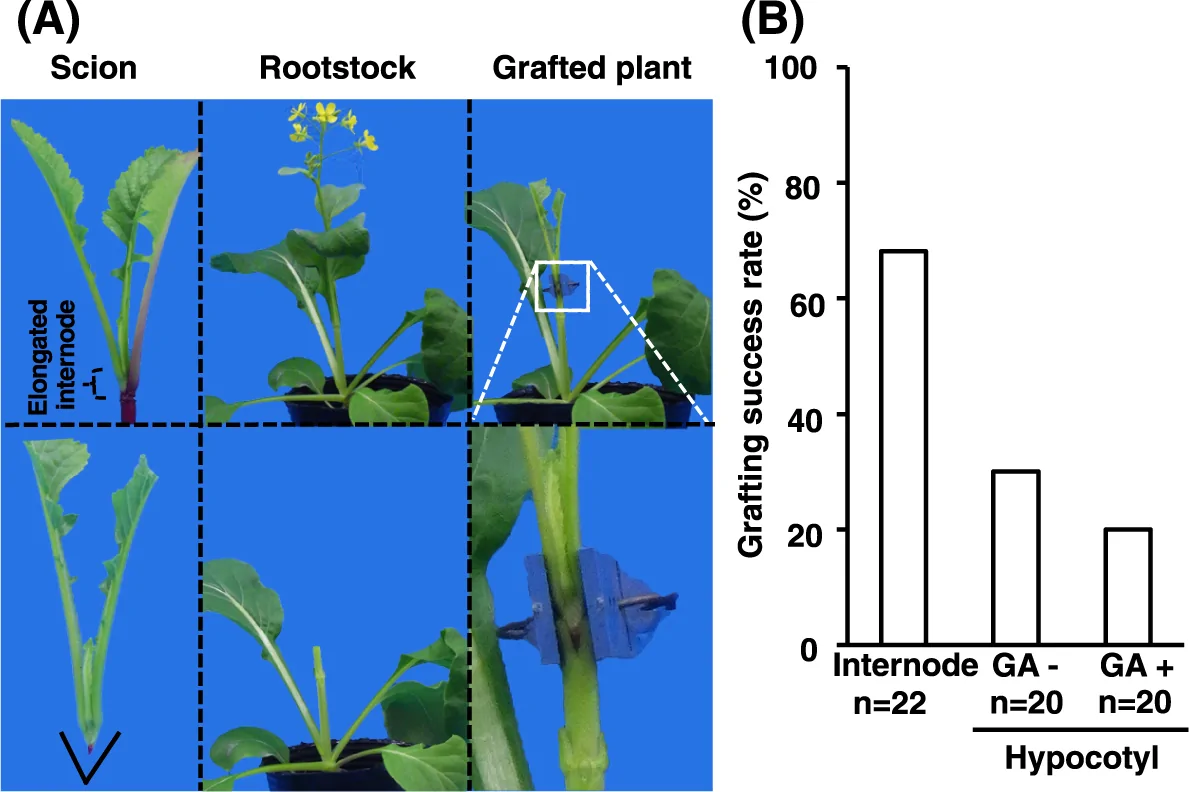
Grafting with elongated internode or hypocotyl as scions. Elongated internodes or hypocotyls of ‘Benikururi’ were used as scions grafted onto the flower stalk of the rootstock ‘CHOY’. (A) Grafting process using GA_3_-induced elongated internodes. (B) Grafting success rates depending on the organ used as the scion. *n* = number of individuals.

To evaluate the effect of elongated internodes on grafting success, we compared grafting success rates using either elongated internodes or hypocotyls of ‘Benikururi’ as scions grafted onto the flower stalk of *B. rapa* cv. ‘CHOY SUM EX CHINA 3’ (hereafter referred to as ‘CHOY’). In this grafting procedure, scions were cut into a V-shape at the target organ of ‘Benikururi’. A vertical slit was then made at the center of the horizontally cut flower stalk of ‘CHOY’, into which the scion was inserted (Fig. 2a).

Grafting success was evaluated seven days after the procedure, based on the emergence of a true leaf from scions, as most unsuccessful grafts exhibited desiccation and failed to survive beyond this point. The success rate for the elongated internode exceeded 68.2%, while that for the hypocotyl was less than 30%, regardless of whether it was treated with GA_3_ or not (Fig. 2b). Therefore, the use of an elongated internode increases the grafting success rate when using the flower stalk of ‘CHOY’ as the rootstock.

**Fig. 3.**
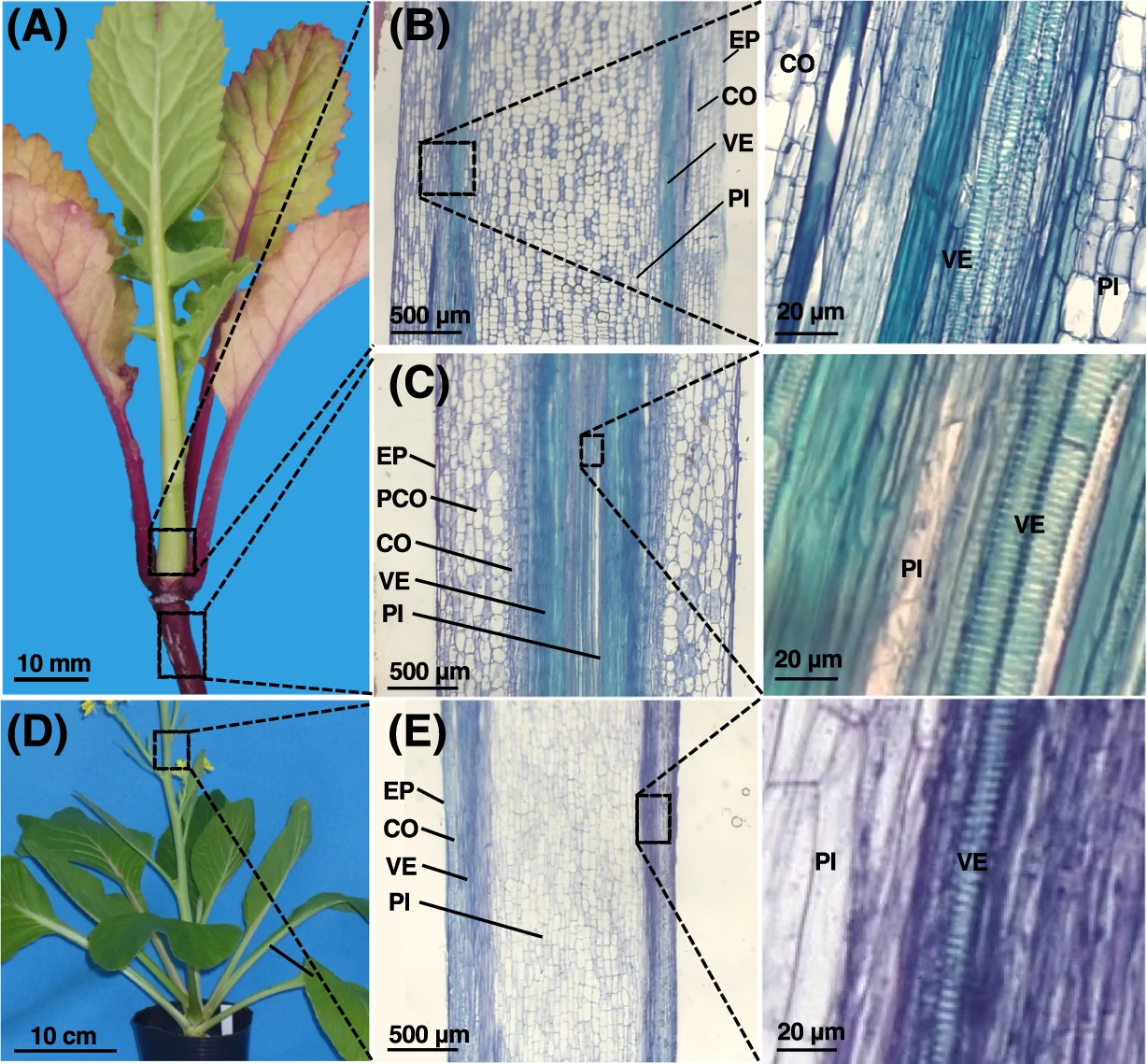
Histological observations of organs used for grafting. (A–C) ‘Benikururi’ at 4 weeks after GA_3_ treatment. (A) Whole plant used as scion. (B, C) Vertical sections of the internode (B) and hypocotyl (C). (D, E) ‘CHOY’ at the flowering stage, used as rootstock. (D) Whole plant. (E) Vertical section of the flower stem. Abbreviations: EP, epidermis; PCO, primary cortex; CO, cortex; VE, vessel; PI, pith.

To understand why the elongated internode was more suitable for grafting than the hypocotyl, we conducted histological observations using paraffin sections (Fig. 3, Supplemental Fig. 5). In GA_3_-treated ‘Benikururi’, the collateral vascular bundles in the elongated internode were arranged concentrically around the central pith, forming a distinct ring-like structure (Fig. 3a–3c). In contrast, vascular bundles in the hypocotyl were more centrally concentrated, with a smaller pith and a more prominent primary cortex. Histological analysis of the flower stalk of ‘CHOY’ revealed a tissue organization closely resembling that of the elongated internode of ‘Benikururi’ (Fig. 3d, 3e), suggesting that this anatomical similarity may contribute to the higher grafting success rate observed with elongated internodes.

### Component transport through the newly connected vascular bundles

**Fig. 4.**
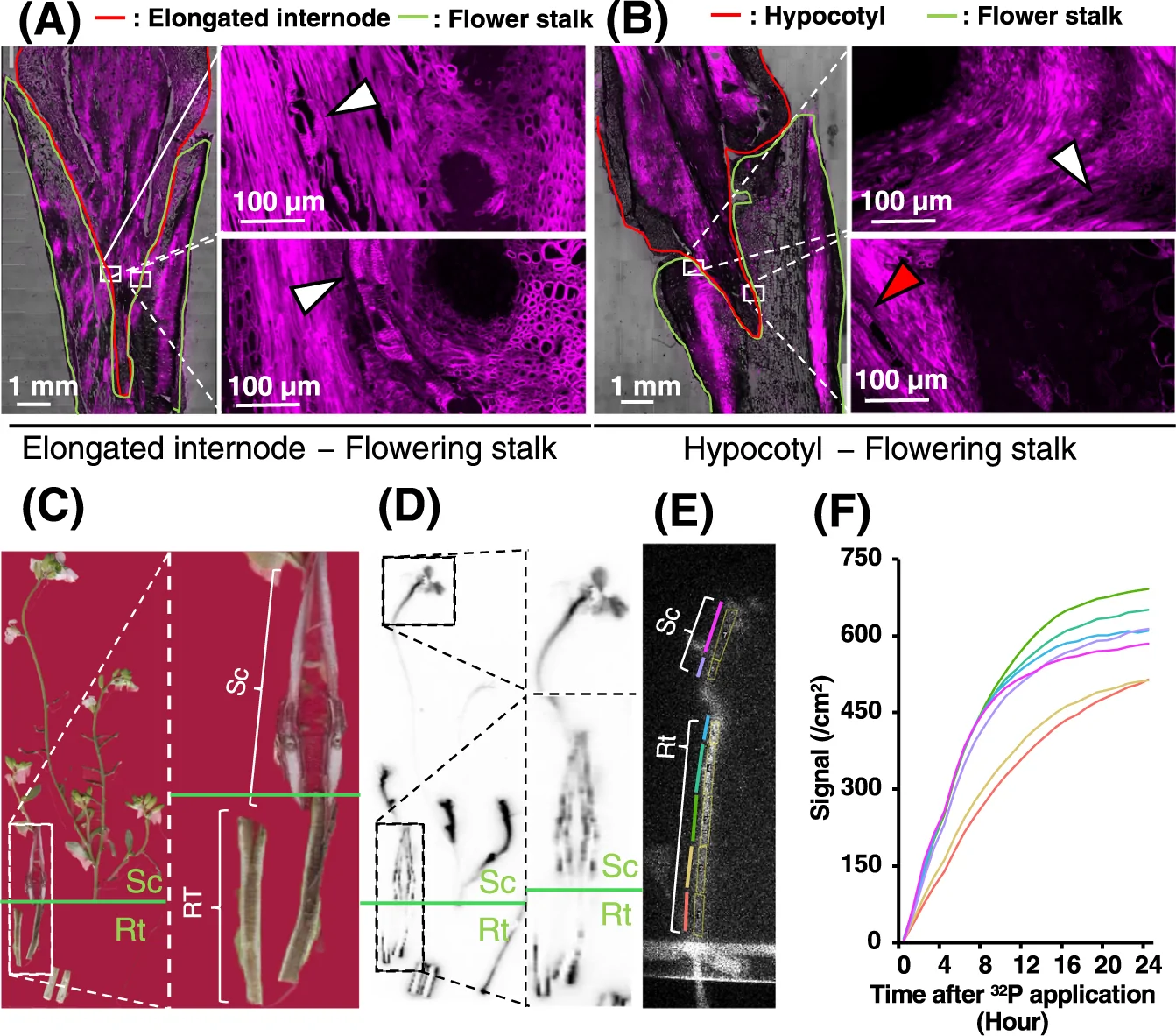
Component transport through newly connected vascular bundles. (A, B) Confocal microscopy images of the graft junctions between GA_3_-induced elongated internode and flower stalk (A), and between hypocotyl and flower stalk (B). The white arrowheads indicate the xylem vessels within the callus-like tissue. The red arrowheads indicate the xylem vessels of scion. (C, D) Detection of phloem transport using ^14^CO_2_ in an individual grafted between the GA_3_-induced elongated internode of ‘Benikururi’ and the flower stalk of ‘CHOY’: normal light image (C) and corresponding radioisotope signal image (D). The green line indicates the boundary between the scion and the rootstock. (E) Radioisotope signal image showing xylem transport of ^32^P absorbed from the roots in the same grafted combination as in (C, D). Signal intensities were recorded in regions marked by different colored bars. (F) Signal intensities at 30-minute intervals corresponding to each region shown in (E). Rt, Rootstock; Sc, Scion.

To determine whether vascular connections between scion and flower stalk would be formed after grafting, we first conducted detailed histological observations of longitudinal sections of grafted junction in both elongated internode–flower stalk and hypocotyl–flower stalk combinations. In both cases, cells developing secondary cell wall structures were observed at the boundary between scion and rootstock (Fig. 4a, 4b), suggesting that vascular reconnection had occurred between the scion and rootstock in both combinations. Notably, in grafts between the elongated internode and the flower stalk, vascular connections were observed across multiple vascular bundles surrounding the pith in both the scion and rootstock (Fig. 4a). In contrast, grafts between the hypocotyl and the flower stalk showed connections between the scion and only a limited portion of the rootstock’s vascular bundles (Fig. 4b). These findings suggest that the higher grafting success rate observed with the elongated internode may be attributed to its more favorable anatomical structure, which promotes efficient vascular reconnection.

Next, to investigate whether the newly connected vascular bundles support functional transport of flowering signals such as FT, we examined the movement of radioisotopes in individuals grafted between the elongated internode and the flower stalk. To assess phloem transport, carbon-14-labeled carbon dioxide (^14^CO_2_)—a radioisotope absorbed by the leaves and incorporated into photosynthetic assimilates—was applied exclusively to the lower leaves of ‘CHOY’ (Fig. 4c, 4d). In grafted ‘CHOY’ individuals, radioactivity was detected in the upper parts of the shoot, where ^14^CO_2_ had not been applied directly, indicating successful upward translocation through the phloem. To evaluate the speed of xylem transport across the graft junction, we supplied phosphorus-32 (^32^P-phosphate), a radioisotope absorbed from the roots and transported via the xylem (Fig. 4e, 4f). The consistent signal intensity across the graft junction suggests that the speed of xylem transport was not reduced by grafting. These findings collectively support that vascular connectivity is not a limiting factor for FT movement or the speed of floral induction in our grafting system.

### Effect of GA treatment on early flowering in the presence of *FT*

**Fig. 5.**
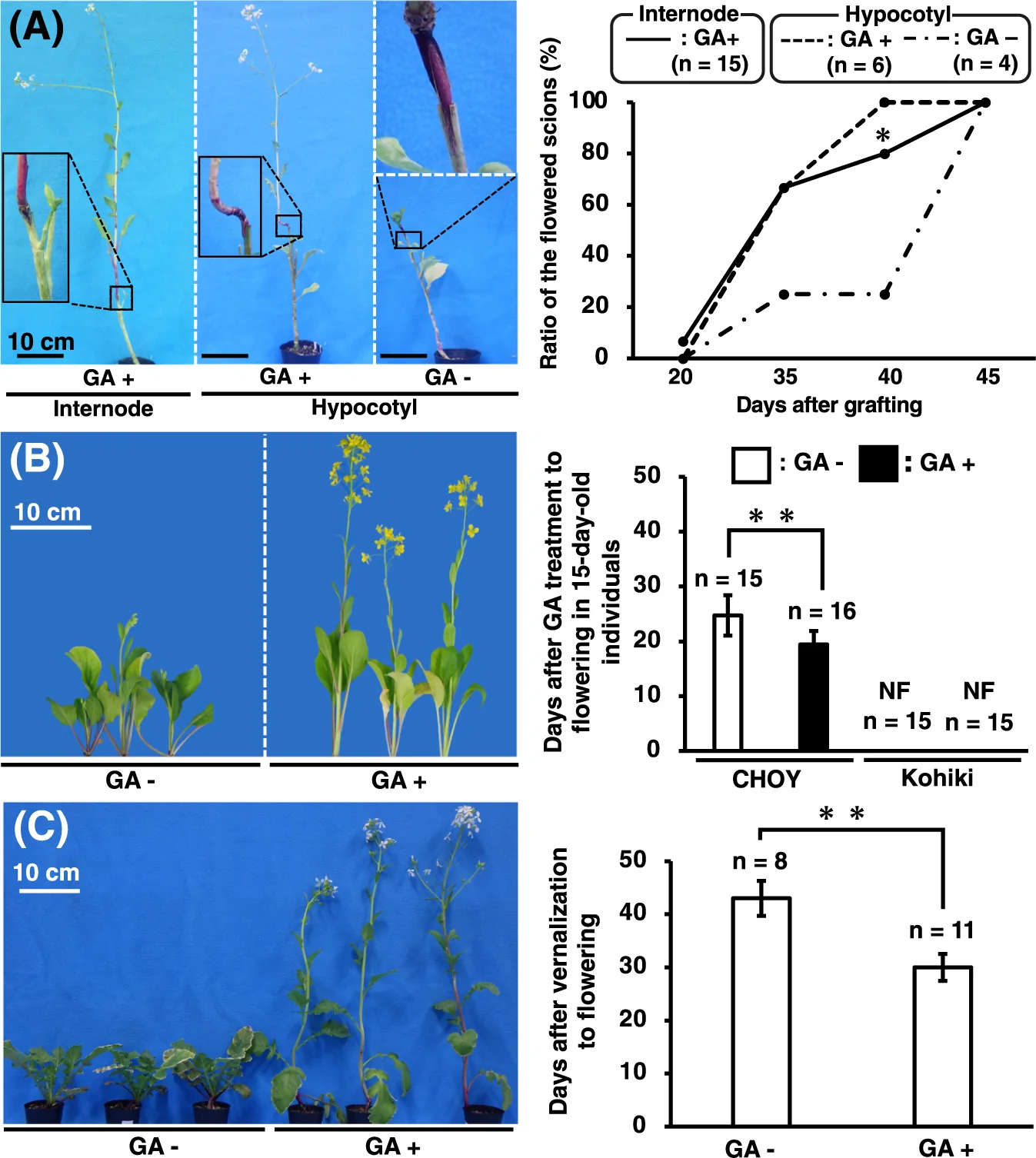
Effect of GA_3_ treatment on early flowering. (A) Flowering response in grafted scions treated with GA_3_. The line graph shows the proportion of scions that flowered over time after grafting onto the flowering stem of ‘CHOY’. * indicates a significant difference in flowering ratio between GA_3_-treated and untreated hypocotyls (Fisher’s exact test, p < 0.05). (B) Effect of GA_3_ on flowering in *FT*-expressing cultivar ‘CHOY’. The image shows ‘CHOY’ plants three weeks after GA_3_ treatment. The graph presents the number of days to flowering in ‘CHOY’ and ‘Kohiki’ with or without GA_3_ treatment. ** indicates a significant difference in flowering time between GA_3_-treated and untreated plants (t-test, p < 0.01). NF: No flowering. Bars and error bars represent means and standard deviations, respectively. (C) Effect of GA_3_ on flowering in plants with *FT* expression induced by vernalization. The image shows ‘Benikururi’ individuals 30 days after transfer from vernalization to non-vernalization conditions. The graph illustrates days to flowering in GA_3_-treated and untreated plants. GA_3_ was applied once per week for three consecutive weeks during the vernalization period. ** indicates a significant difference in flowering time between GA_3_-treated and untreated plants (t-test, p < 0.01). GA+: GA_3_-treated; GA–: untreated. Bars and error bars represent mean values and standard deviations, respectively. *n* = number of individuals.

All grafted scions eventually flowered within 45 days after grafting, regardless of GA_3_ treatment and/or grafted organ. However, the timing of flowering varied depending on GA_3_ treatment. To evaluate whether GA_3_ affects the speed of floral transition, we monitored flowering time after grafting (Fig. 5a). By 40 days after grafting, 12 out of 15 scions with GA_3_-treated elongated internodes (80%) and all 6 scions with GA_3_-treated hypocotyls had flowered, whereas only 1 out of 4 scions grafted with untreated hypocotyls (25%) had flowered. To examine whether GA_3_ alone can induce flowering, we applied GA_3_ to ‘Benikururi’ at the same developmental stage used for scion grafting. However, this treatment failed to induce flowering even after five months (Supplemental Fig. 6). These results suggest that GA_3_ alone is insufficient to trigger flowering, and that GA_3_ promotes earlier flowering only in the presence of FT supplied by the rootstock. However, due to the limited sample size in the hypocotyl-to-flower-stalk graft group, we were unable to definitively conclude whether the floral promotive effect of GA_3_ is strictly dependent on FT.

To confirm the effect of GA on flowering in the presence of FT, we performed two additional experiments (Fig. 5b, 5c). First, we compared the flowering time of ‘CHOY’ and ‘Kohiki’ plants with and without GA_3_ treatment (Fig. 5b). These two cultivars were selected because they differ in *FT* expression under non-vernalized conditions. ‘CHOY’ consistently expresses *FT* as early as one week after sowing, even without vernalization (Nishikawa *et al.* 2023), whereas the *B. rapa* turnip cultivar ‘Kohiki’ requires cold exposure to induce *FT* expression. Therefore, this contrast made them suitable materials for assessing the effect of GA_3_ under FT-present versus FT-absent conditions. Plants of both cultivars were grown in a fully enclosed cultivation room under controlled conditions (22°C, 14 h light/10 h dark, 06:00–20:00). GA_3_-treated ‘CHOY’ plants flowered approximately seven days earlier than untreated controls. Under the same conditions, a similar acceleration of flowering was observed in another *B. rapa* cultivar, ‘Yellow Sarson’, which does not require vernalization and constitutively expresses *FT* due to loss-of-function mutations in all *FLC* genes (Supplemental Fig. 7). In contrast, the *B. rapa* turnip cultivar ‘Kohiki’ did not flower under the same non-vernalization conditions, regardless of GA_3_ treatment. These findings confirm that GA_3_ alone is insufficient to induce flowering and that *FT* expression is essential for GA_3_-mediated flowering promotion. They also suggest that GA_3_ enhances the plant’s responsiveness to FT rather than directly initiating flowering. Alternatively, GA_3_ may act by suppressing FT antagonists, thereby facilitating earlier floral transition.

To evaluate the broader applicability of the GA_3_-induced promotion of flowering in the presence of FT in vernalization-requiring Brassicaceae cultivars, we further examined GA_3_ treatment in ‘Benikururi’, a cultivar that expresses *FT* only after vernalization (Fig. 5c). Plants were treated with GA_3_ three times at weekly intervals, beginning immediately after the start of a four-week vernalization period. When cultivated in a greenhouse with supplemental lighting (MF250:L/BU-SC-2, Panasonic, Japan; 07:00–19:00) from 21 February to 25 March 2025, GA_3_-treated plants flowered within 30 ± 2.55 days (mean ± SD), whereas untreated plants flowered significantly later, at 43 ± 3.30 days (mean ± SD) (Supplemental Fig. 8).

These results reinforce the idea that GA_3_ enhances sensitivity to FT-mediated flowering signals rather than acting independently. This finding highlights the potential utility of GA_3_ treatment in accelerating flowering even in Brassicaceae crops that require vernalization. Notably, this promotive effect was also observed in the *B. rapa* cultivar ‘Tsukena no 2’, which carries two cold-insensitive *FLC* alleles (*BrFLC2* and *BrFLC3*) and typically requires prolonged cold exposure to flower (Supplemental Fig. 9). In our grafting experiments, the scions of ‘Tsukena no 2’ with GA_3_-treated elongated internodes flowered within 31 days after grafting, demonstrating that GA_3_ can effectively accelerate flowering in cultivars with a strong vernalization requirement when FT is present.

### Effect of GA treatment on the expression of flowering-related genes

To assess how GA_3_ treatment influences the expression of flowering-related genes in the shoot apex under FT-present and FT-absent conditions, we performed RNA-seq analysis on two *B. rapa* cultivars, ‘CHOY’ and ‘Kohiki’, at five and seven days after GA_3_ application (Supplemental Fig. 1). The reason for not using ‘Benikururi’ in this analysis is that *FT* expression in this cultivar requires vernalization, which induces broad changes in gene expression and plant development. These cold-induced genes could confound the interpretation of GA_3_-specific responses under *FT*-expressing conditions.

**Fig. 6.**
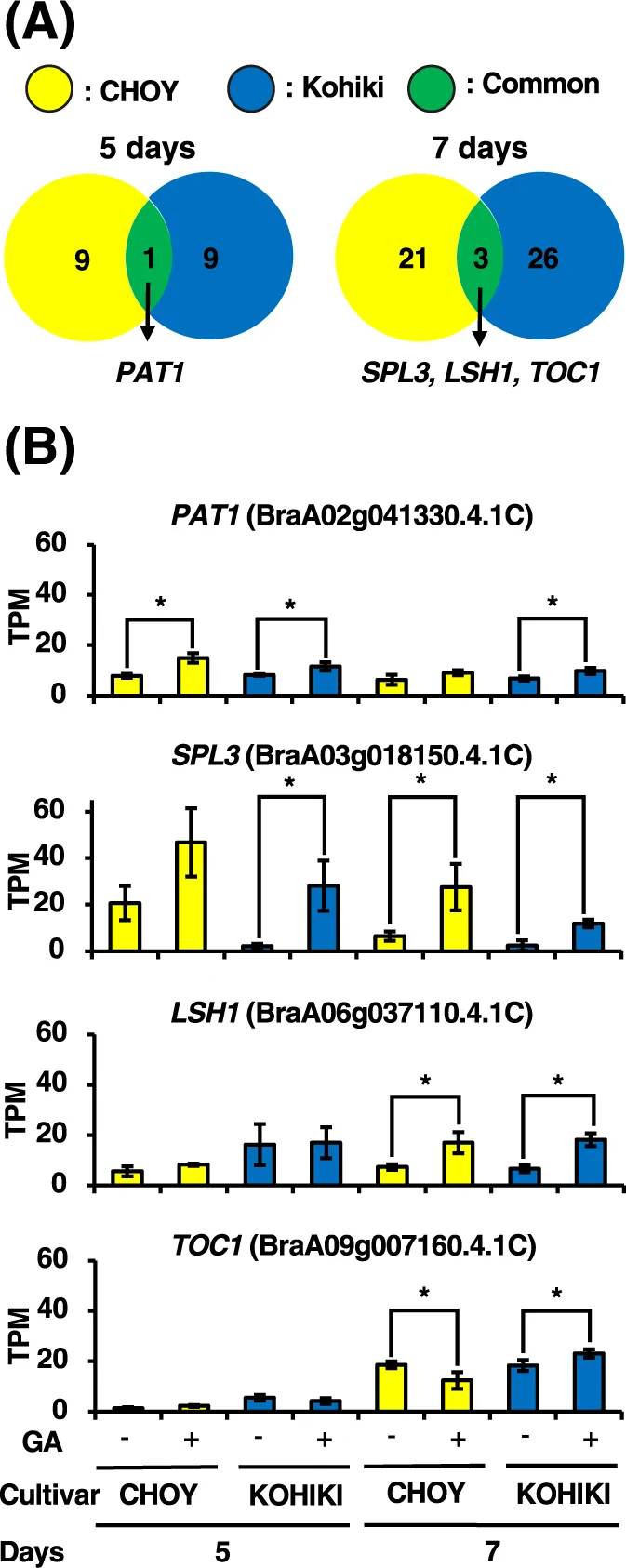
Effect of GA_3_ treatment on gene expression in the shoot apex. RNA-seq analysis was conducted using RNA extracted from the shoot apex region of ‘CHOY’ and ‘Kohiki’ at 5 and 7 days after GA_3_ application. (A) Pie charts showing the number of flowering-related genes that were differentially expressed in each cultivar following GA_3_ treatment. (B) TPM values for genes commonly differentially expressed in both cultivars at either 5 or 7 days. Bars and error bars represent the mean ± SD deviation from three biological replicates. Asterisks indicate samples showing significant differences (t-test, p < 0.05) between GA_3_-treated and untreated plants within the same cultivar and time point. Although *PAT1* and *LSH1* have four and two splicing variants, respectively, only one representative isoform is shown, as the TPM values were identical across all variants.

We first identified differentially expressed genes (DEGs) following GA_3_ treatment (Fig. 6a). At 5 days post-treatment, 1,044 DEGs were detected in ‘CHOY’ and 889 in ‘Kohiki’. These numbers slightly increased at 7 days post-treatment, reaching 1,047 in ‘CHOY’ and 1,454 in ‘Kohiki’. Next, we screened the DEGs for genes known to be involved in flowering in Arabidopsis (Supplemental Table 3). At 5 days after treatment, 10 such flowering-related genes were identified in both ‘CHOY’ (Supplemental Table 4) and ‘Kohiki’ (Supplemental Table 5). At 7 days post-treatment, the number of flowering-related DEGs increased to 24 in ‘CHOY’ (Supplemental Table 6) and 29 in ‘Kohiki’ (Supplemental Table 7).

Among the flowering-related genes identified, we classified them into two categories based on their expression patterns and their putative positions in the flowering regulatory network: (1) genes expressed independently of *FT*, which may act upstream of or in parallel with *FT*, and (2) genes expressed specifically in the presence of *FT*, likely functioning downstream or in coordination with *FT*.

For category (1), we identified four candidates—*PHYTOCHROME A SIGNAL TRANSDUCTION 1* (*PAT1*), *SQUAMOSA PROMOTER BINDING PROTEIN-LIKE 3* (*SPL3*), *LIGHT-DEPENDENT SHORT HYPOCOTYLS 1-LIKE* (*LSH1*), and *TIMING OF CAB EXPRESSION 1* (*TOC1*) were identified as DEGs following GA_3_ treatment in both ‘CHOY’ and ‘Kohiki’ at both time points (Fig. 6b). Among them, *PAT1*, *SPL3*, and *LSH1* were upregulated in both cultivars, while *TOC1* showed opposite expression patterns, suggesting that its regulation may involve factors beyond GA_3_ treatment. Notably, *SPL3* expression increased markedly at 7 days post-treatment, showing a 4.26-fold increase in ‘CHOY’ and a 4.90-fold increase in ‘Kohiki’ relative to pre-treatment levels. In contrast, the maximum changes in *PAT1* and *LSH1* expression were more moderate, with less than a 1.90-fold increase in ‘CHOY’ at day 5 and a 2.72-fold increase in ‘Kohiki’ at day 7.

For category (2), we identified four candidates—*VERNALIZATION1* (*VRN1*), *SHORT VEGETATIVE PHASE* (*SVP*), *FAR-RED ELONGATED HYPOCOTYL 1* (*FHY1*), and *DWARF IN LIGHT 2* (*DFL2*) were identified as ‘CHOY’-specific DEGs at both 5 and 7 days after treatment (Supplemental Tables 4, 6). *FHY1* was excluded due to inconsistent expression trends across time points. The remaining three genes may also be upregulated by GA_3_ under *FT*-expressing conditions; however, their fold changes were relatively modest (less than 1.76-fold), especially when compared to the pronounced induction of *SPL3*. Moreover, it remains unclear whether their upregulation was a direct effect of GA_3_ or a secondary consequence of early flowering in this analysis.

These results suggest that *SPL3*, a transcription factor known to activate flowering-related genes in the GA pathway in Arabidopsis (Wang 2014), is a strong candidate mediating GA_3_-induced in the *FT* expressing cultivar ‘CHOY’. In support of this, the expression of *SPL3* downstream targets such as *LEAFY* (*LFY*), *FRUITFULL* (*FUL*), and *APETALA1* (*AP1*), which are known to promote flowering in *Arabidopsis* (Jung *et al.* 2016), was slightly upregulated in ‘CHOY’. However, their TPM values remained generally very low (Supplemental Fig. 10).

## Discussion

In this study, we established a rapid flowering system for Brassicaceae plants by grafting GA_3_-induced elongated internodes as scions onto the flowering stalk of an *FT*-expressing rootstock. This integrated strategy enhances both the practicality and efficiency of floral induction in rosette-type Brassicaceae crops. In addition, our results suggest that GA_3_ treatment applied to scions not only promotes internode elongation but also accelerates floral induction in the presence of FT.

In GA_3_ treatment for internode elongation in scions, ensuring the penetration of the GA_3_ solution around the shoot apex is crucial. When the shoot apex is covered by leaves after prolonged growth (Fig. 1d, Supplemental Fig. 3), internode elongation may not occur. Therefore, the optimal timing of GA_3_ treatment should depend on the growth rate and the degree of leaf coverage around the shoot apex, suggesting that breeders should carefully determine the appropriate timing of GA_3_ treatment for each crop.

The technique for grafting described in this study (Fig. 2a, Supplemental Fig. 4) was based on the method described by Motoki *et al.* (2022), which involves covering the scion with a black plastic bag after grafting onto a compact rootstock. This approach also improved grafting efficiency in our system. Compared with our previous grafting process, which required large equipment to maintain humidity around the entire plant, the method used in this study reduces space requirements by maintaining humidity only at the graft junction. This localized humidity control minimizes transpiration from the scion while still allowing the rootstock to perform photosynthesis. Additionally, ‘CHOY’, used as the rootstock, has leaves that emerge at a sharp angle relative to the stalk, compared with the rat-tail radish used by Motoki *et al.* (2019). Therefore, when ‘CHOY’ is used as the rootstock, the required growing space is reduced compared to lines with rosette-type leaves that extend more horizontally, allowing for the simultaneous grafting of multiple individuals.

Histological observations suggest two potential reasons for the difficulty encountered when grafting between hypocotyls and flower stalks. First, due to differences in vascular bundle arrangements, the cambium layers of the scion and rootstock may fail to align properly, thereby impeding vascular reconnection and graft establishment. Second, in hypocotyls excised for grafting, the central region is primarily composed of fully differentiated xylem cells, which lack the capacity for dedifferentiation since they are non-living cells. Improving grafting success with hypocotyls would eliminate the need for internode elongation, reducing both the time and labor required for GA_3_ treatment. Moreover, this approach could enable the induction of flowering in cultivars that do not respond to GA-induced internode elongation. Therefore, to overcome the limitations of hypocotyl grafting, alternative approaches like side or saddle grafting should be considered.

SPL3 appears to be induced by GA_3_ independently of FT, but its ability to activate downstream floral genes may depend on FT presence. In Arabidopsis, *SPL3* is known to induce floral meristem formation by directly activating the expression of flowering-related genes such as *LFY*, *FUL* and *AP1* (Jung *et al.* 2016). In our study, *SPL3* was upregulated in both ‘CHOY’ (*FT*-expressing) and ‘Kohiki’ (*FT*-deficient). However, *LFY*, *FUL*, and *AP1* showed increased expression only in ‘CHOY’, suggesting that SPL3 alone is not sufficient to activate these targets without FT. Jung *et al.* (2020) found that applying GA after vernalization led to early flowering in *R. sativus*, and attributed this effect to GA-induced upregulation of *FT* and *SOC1*. However, they did not report on *SPL3* expression, leaving it unclear whether this gene was involved in their system. In contrast, neither ‘CHOY’ nor ‘Kohiki’ showed consistent upregulation of *FT* or *SOC1* across both 5 and 7 days after GA_3_ treatment. The discrepancy between their results and ours may be due to differences in the sampled tissue. Jung *et al.* (2020) analyzed entire shoots following GA_3_ spraying, whereas our study examined only the shoot apex region after localized GA_3_ application. To clarify the role of *SPL3* in GA-mediated flowering acceleration in *B. rapa*, and how it mechanistically differs from Arabidopsis and the GA-responsive flowering pathway reported in *R. sativus* by Jung *et al.* (2020), future analysis using *SPL3* mutants will be essential. Additionally, considering the timing of RNA sampling is critical. In our study, samples were collected at five and seven days after GA_3_ treatment. While slight change in *LFY*, *FUL*, and *AP1* expression were observed, more pronounced expression changes may require sampling at later time points beyond day 7. Conversely, to detect expression changes in early GA-responsive genes such as those involving DELLA proteins, earlier sampling before day 5 may be necessary. Further studies with refined sampling time points will be necessary to capture the full profile of gene expression response following GA_3_ treatment.

The rapid flowering induction technique using GA_3_-treated scions in grafting can significantly accelerate the breeding of late-flowering Brassicaceae plants when combined with DNA marker-assisted selection (MAS). MAS allows for the identification of desirable individuals without relying on phenotype-based selection, thereby eliminating the need for a cultivation period to confirm the late-flowering trait. In Brassicaceae, a key breeding strategy for inducing late flowering involves the introduction of *FLC* alleles that are insensitive to cold temperatures. Recent genetic studies have successfully identified such *FLC* alleles in various Brassicaceae species, including *B. rapa*, *B. oleracea*, and *R. sativus* (Kitamoto *et al.* 2014, Li *et al.* 2022, Wang *et al.* 2018). For some of these species, DNA markers that distinguish specific *FLC* alleles are already available (Okazaki *et al.* 2007, Schranz *et al.* 2002). The grafting system allows up to three generation cycles per year, each cycle completed within 120 days. The integration of GA_3_-induced grafting and MAS facilitates the rapid and efficient development of near-isogenic lines carrying late-flowering *FLC* alleles on desirable genetic backgrounds. Notably, this grafting approach enables flowering within two months after sowing, even in *B. rapa* cultivars such as ‘Tsukena no 2’, which possess two cold-insensitive alleles, *FLC2* and *FLC3*, and typically require prolonged cold exposure to flower (Supplemental Fig. 9). In contrast, development of the late-flowering cultivar ‘ITOSAI No. 1’, which harbors both *FLC2* and *FLC3*, required more than nine years using MAS-based breeding (Kitamoto *et al.* 2023). By combining FT supplementation from the rootstock with GA_3_-induced flowering in the scion, flowering can now be achieved within two and a half months, accelerating the development of late-flowering Brassicaceae crops.

Consequently, the combined use of *FT*-expressing rootstocks and gibberellin-induced scions for grafting broadens the applicability of this strategy to cultivars with diverse shoot morphologies, including rosette-type plants with short internodes, and greatly enhances the efficiency of breeding late-flowering Brassicaceae crops.

## Author Contribution Statement

MH established the grafting protocol using GA_3_ treatment and conducted histological observations. SS and MY performed grafting and investigated flowering time post-grafting. KM, YM and TS prepared the RNA-seq libraries and analyzed gene expression levels. SM observed paraffin-embedded sections using confocal microscopy. KT and NK performed radioisotope analysis. HT designed and supervised the study and wrote the manuscript. All authors contributed to the writing of the manuscript.

## Supplementary Material

Supplemental Figures

Supplemental Tables
