# Supplementary material for: Gibberellin-mediated internode elongation for grafting and early flowering induction in Brassicaceae crops
Source: Breed Sci. 2026 Jan 16;76(2):133–43. doi: 10.1270/jsbbs.25036 (PMC13494322; doi:10.1270/jsbbs.25036)
Supplement: Supplementary file 1 [file 76_133_s1.pdf]

doi: 10.1270/jsbbs.25036

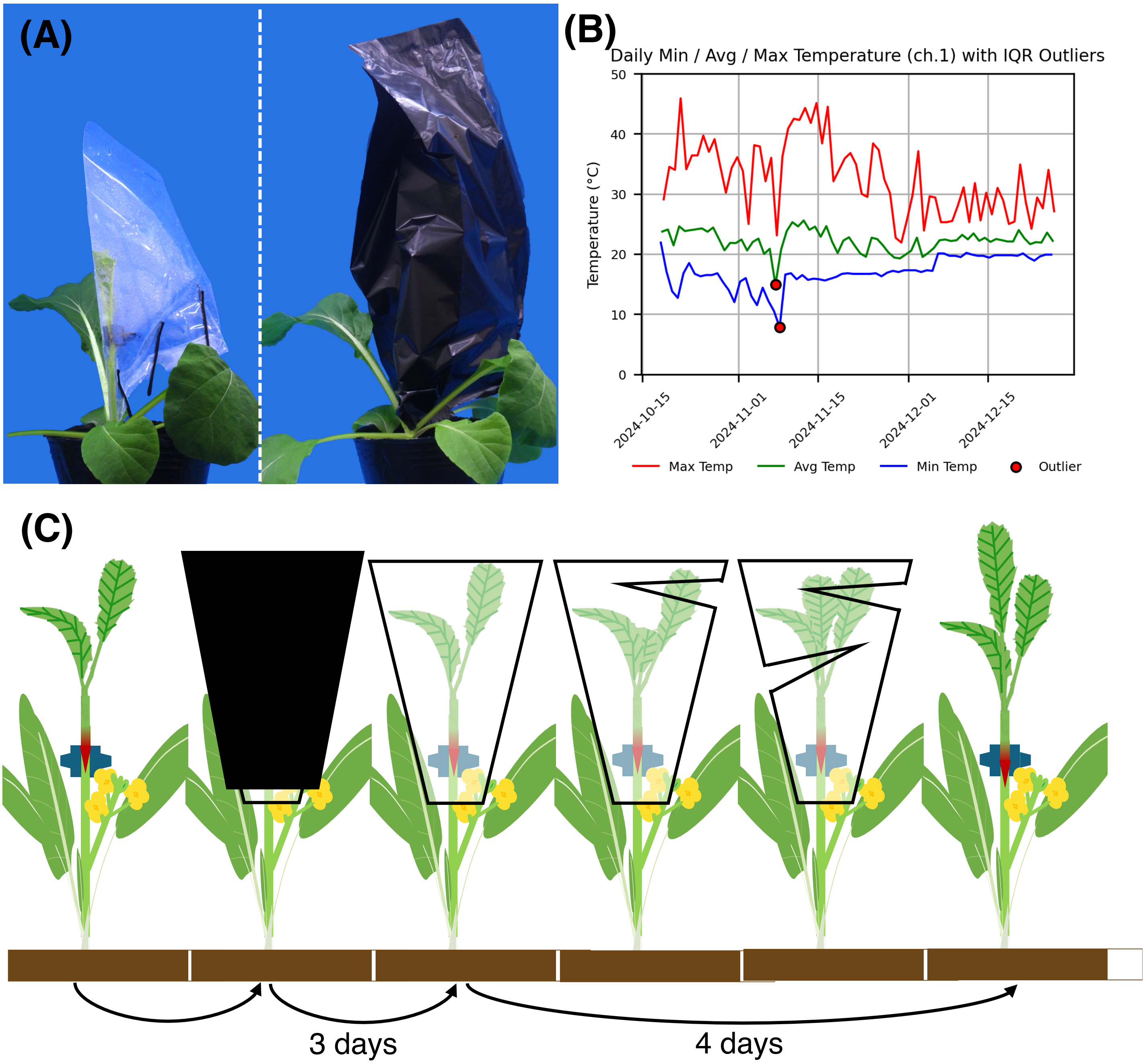

Supplemental Figure 1. The management of scion after grafting.
(A) Appearance of plants immediately after grafting. To maintain humidity, the scion is covered with a clear plastic bag sprayed with water on the inside. A black plastic bag is then placed over the clear bag to suppress transpiration. **(B)** Temperature in a greenhouse at Ishikawa Prefectural University (from October 18 to December 27, 2024). Outliers are indicated as values exceeding the upper limit (Q3 + 1.5 × IQR) or falling below the lower limit (Q1 − 1.5 × IQR), based on the interquartile range (IQR) method. **(C)** Flowchart of post-grafting management. Three days after grafting, the black plastic bag is removed. To acclimate the scion, a small hole is made in the clear plastic bag and gradually enlarged over the next four days. One week after grafting, all plastic bags are removed.

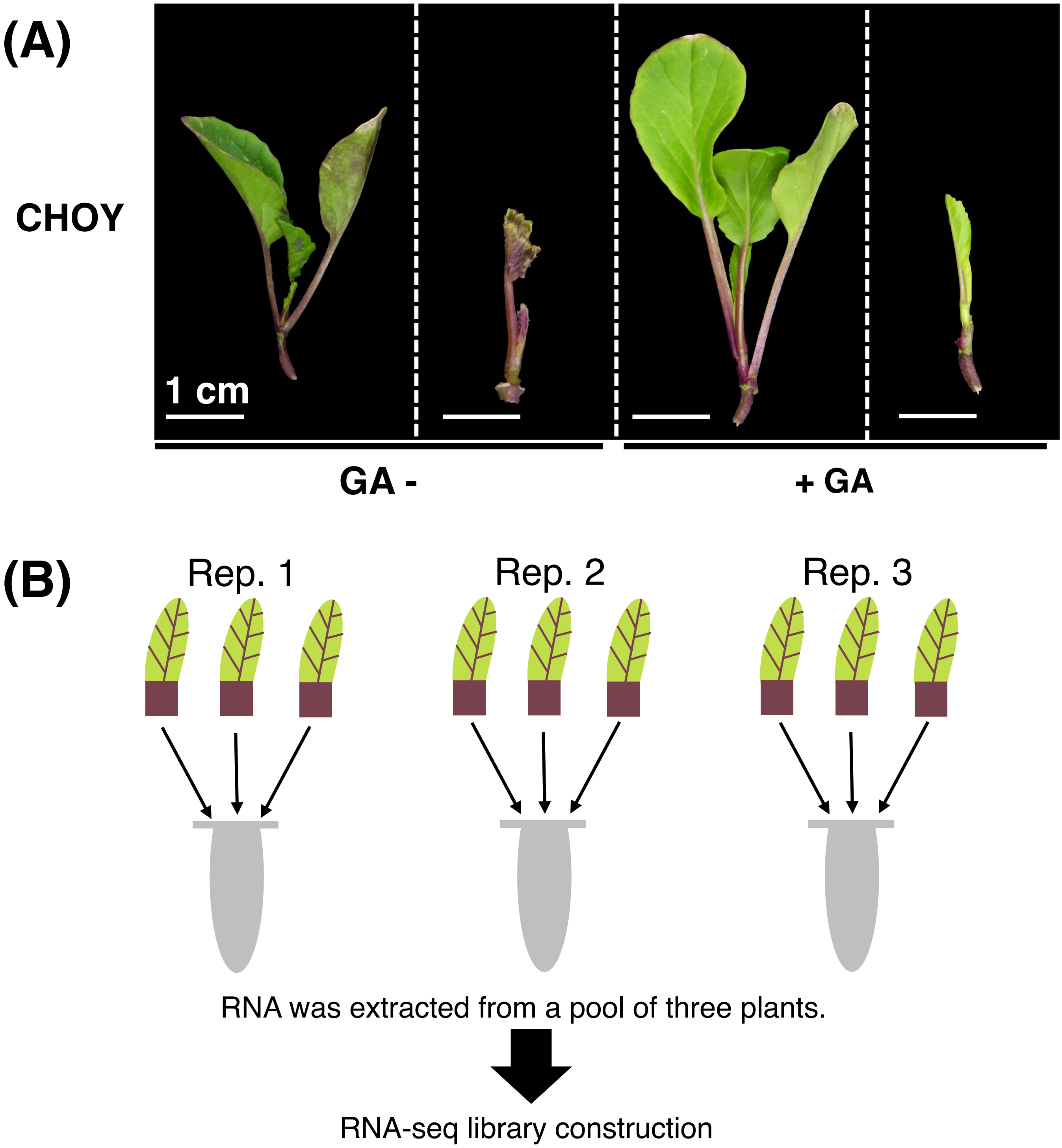

Supplemental Figure 2. Sampling for RNA-seq.
(A) RNA-seq samples of 'CHOY' collected at 22 days after sowing, with or without GA_3 treatment applied at 15 days after sowing. Plants were cultivated in a fully enclosed cultivation room at under controlled conditions (22℃, 14 h light / 10 h dark, 06:00–20:00). For the RNA extraction, the shoot apex region and one unexpanded leaf were sampled. Tissue from three individuals plants were pooled to preparer a single biological replicate, and three biological replicate samples were used for RNA extraction and library construction. **(B)** Flowchart illustrating the sampling procedure for RNA extraction.

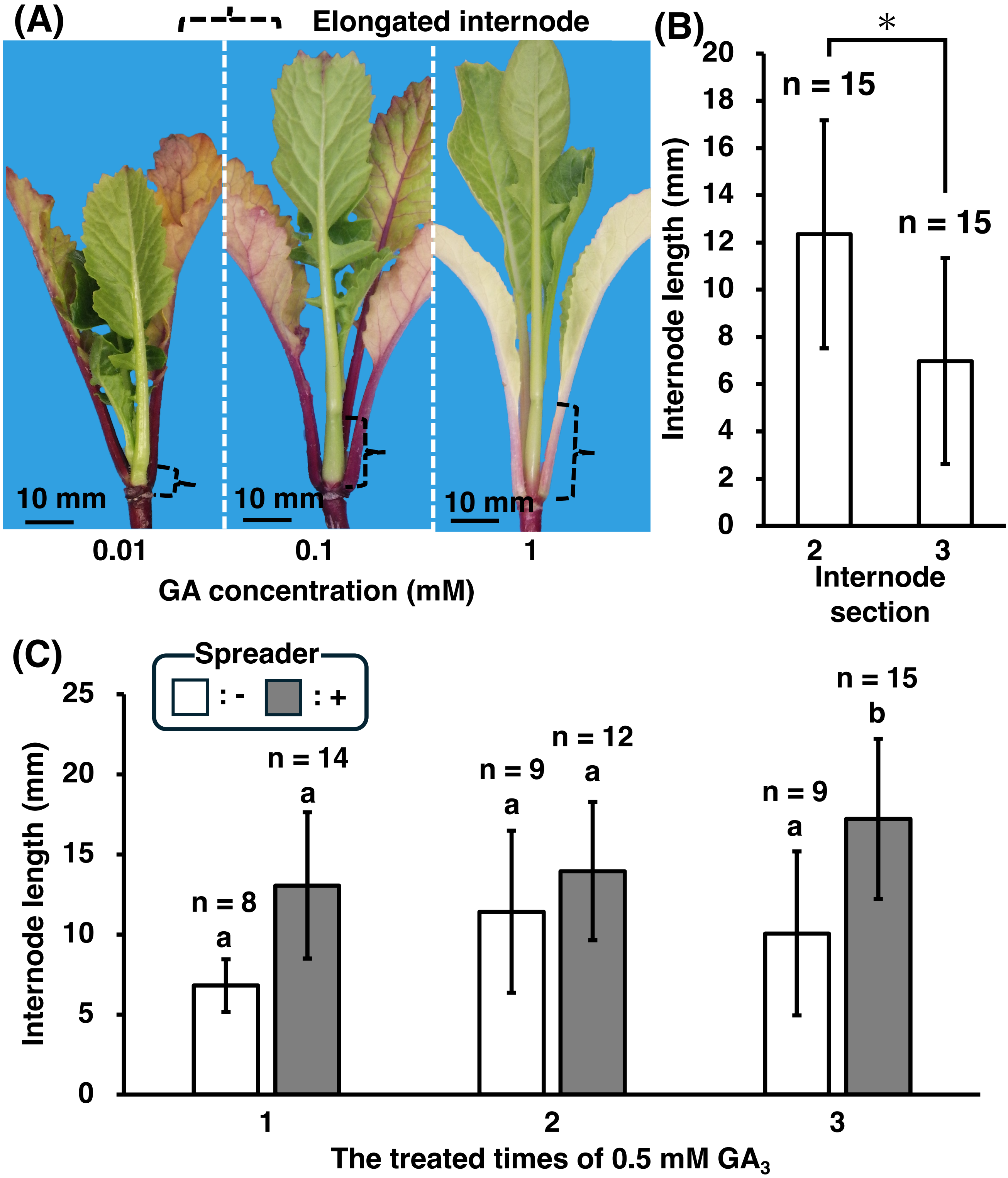

Supplemental Figure. 3 Internode elongation of 4 weeks after GA_3 treatment in 'Benikururi'.

(A) GA_3 was applied three times at each concentration, every other day, starting when two main leaves had emerged. **(B)** Internode elongation in the second and third sections following three applications of 0.5 mM GA_3 with a spreader. Bars and error bars represent the mean ± standard deviation (SD). *n* = number of individuals. * = $p < 0.05$ (two-tailed t-test). **(c)** Internode lengths measured four weeks after the initial application of 0.5 mM GA_3 solution without spreader. "n" indicates the number of treated individuals. Bars and error bars represent mean values and S.D, respectively. Different letters on the bars indicate statistically significant differences ($P < 0.05$) according to Tukey's test. Note:The effect of the spreader is assessed by comparison with the corresponding "with spreader" condition presented in Fig. 1C.

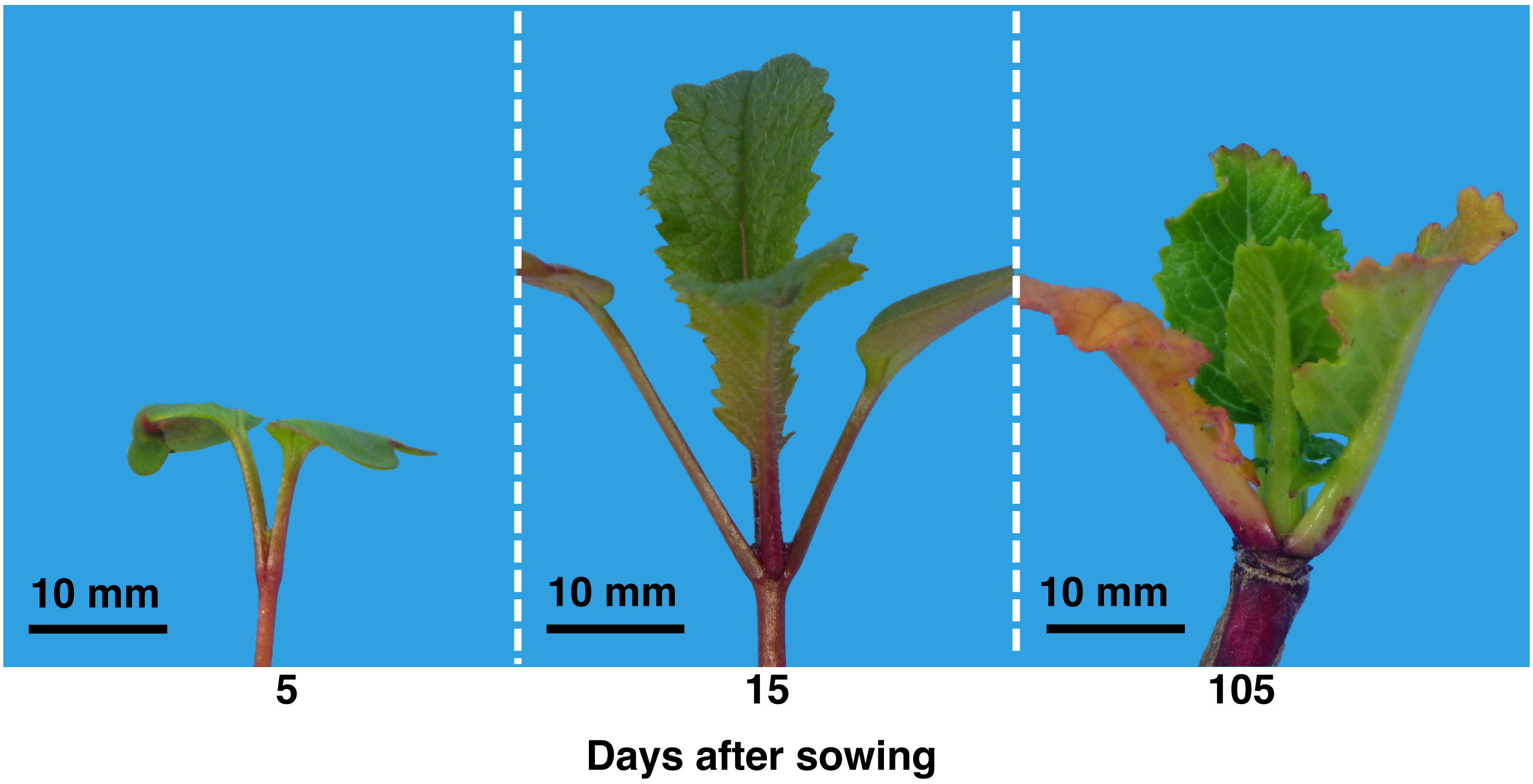

Supplemental Figure 4. Growth of ‘Benikururi’ at different days after sowing.

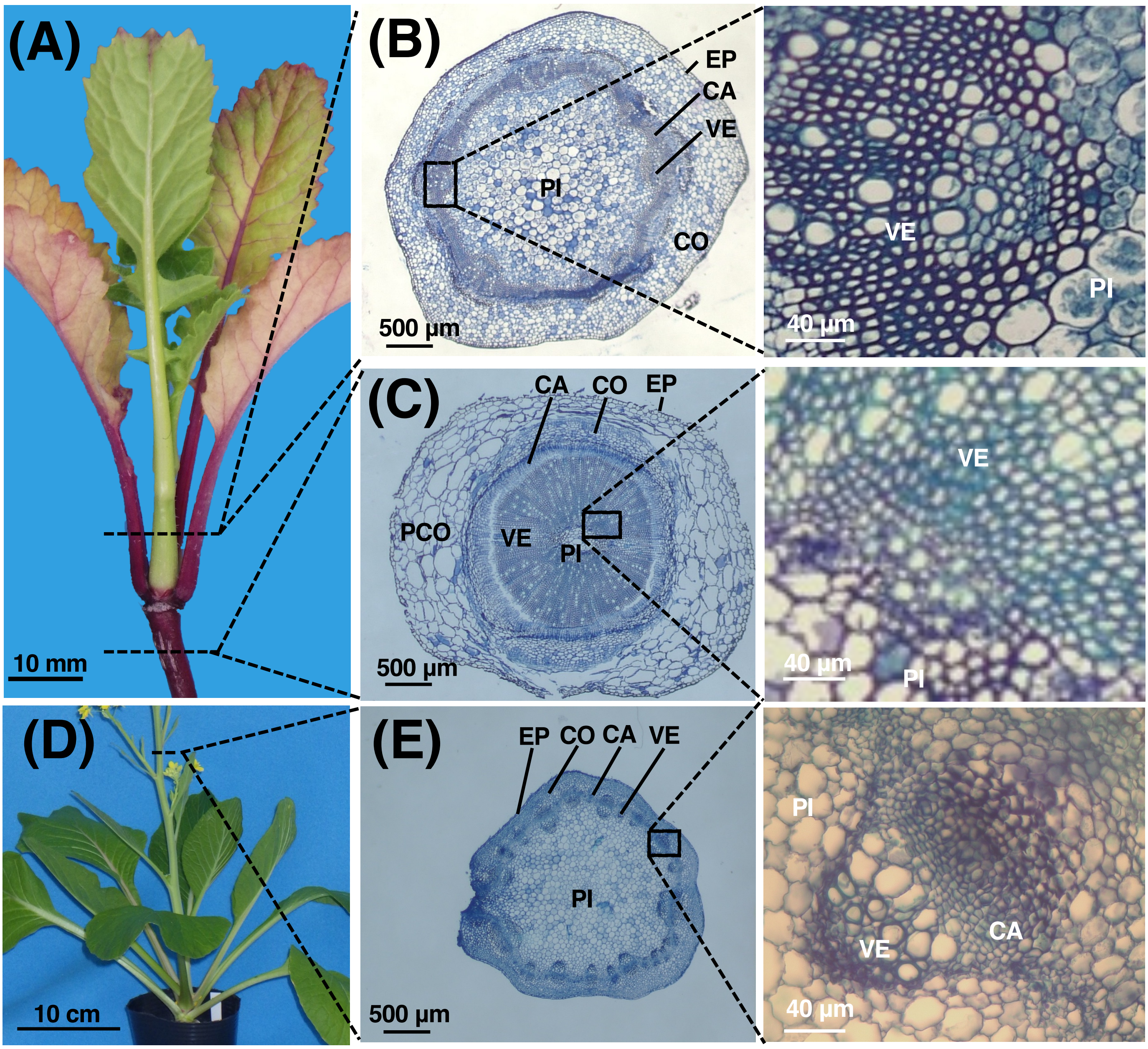

Supplemental Figure 5. Horizontal sections of organs used for grafting.
(A–C) 'Benikururi' at 4 weeks after GA_3 treatment. **(A)** Whole plant used as scion. **(B, C)** Horizontal sections of the elongated internode (B) and hypocotyl (C). **(D, E)** 'CHOY' at the flowering stage, used as rootstock. **(D)** Whole plant. **(E)** Horizontal section of the flower stalk. Abbreviations: EP, epidermis; PCO, primary cortex; CO, cortex; CA, cambium; VE, vessel; PI, pith.

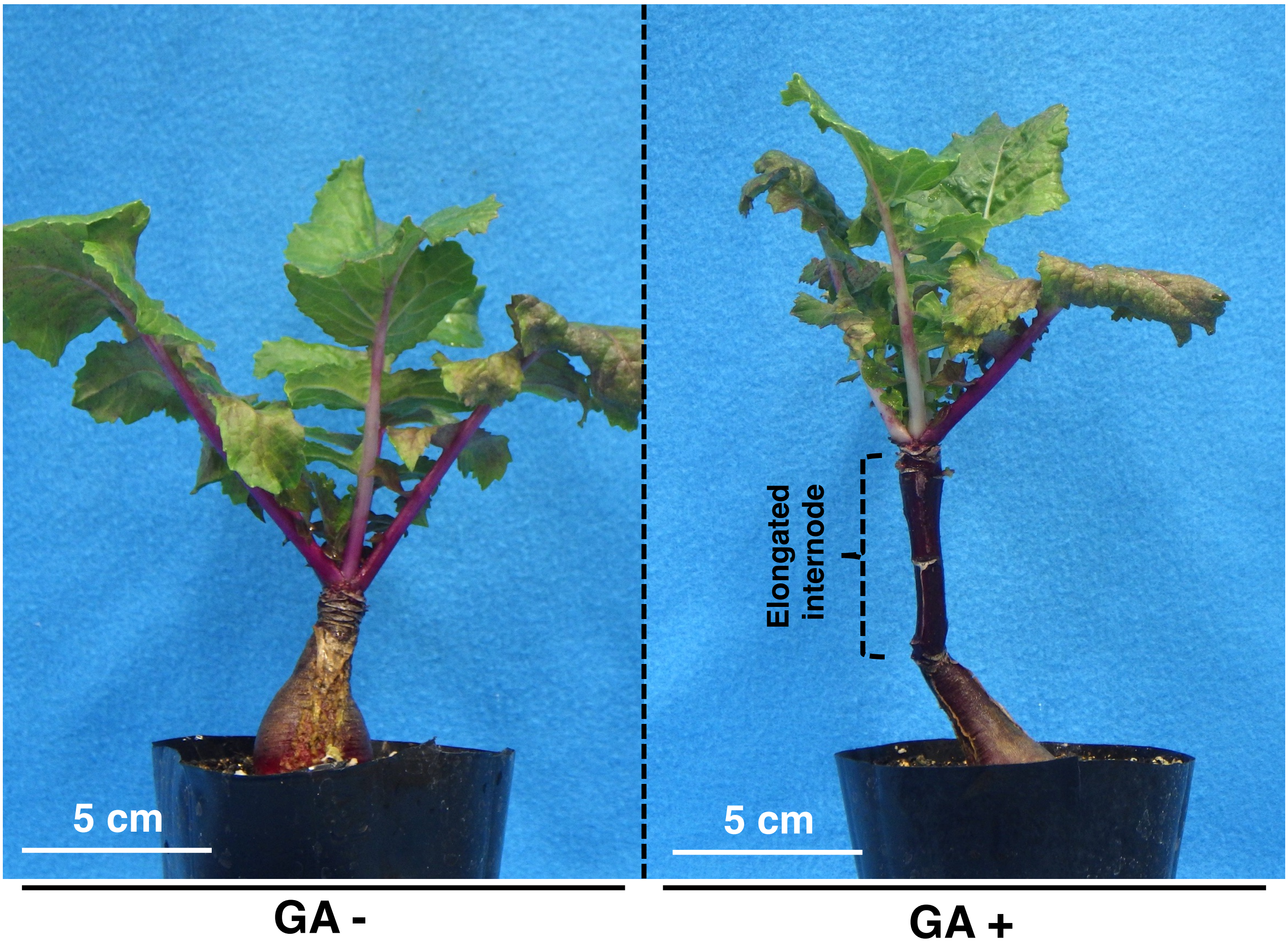

Supplemental Figure 6. No flowering induced by GA_3 treatment alone.
'Benikururi' plants five months after sowing, with and without GA_3 treatment applied at the juvenile stage (15 days after sowing, as shown in Supplemental Figure 4).

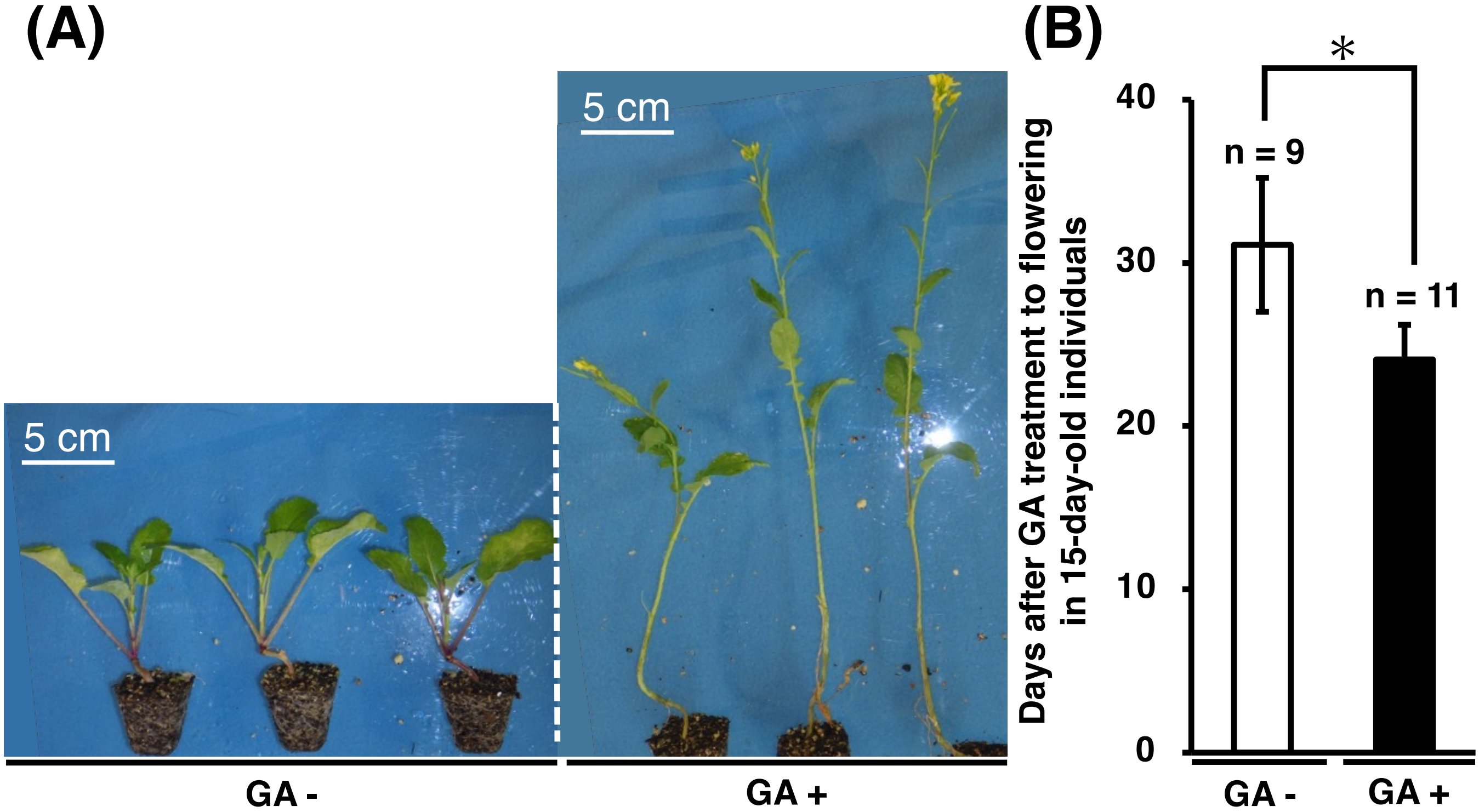

Supplemental Figure 7. Effect of GA_3 on early flowering in 'Yellow Sarson'.
(A) 'Yellow Sarson' plants four weeks after GA_3 treatment. **(B)** Graph showing the number of days to flowering in 'Yellow Sarson' with and without GA_3 treatment. Bars and error bars represent the mean ± standard deviation (SD). *n* = number of individuals. GA+ and GA− indicate individuals treated with GA_3 and untreated individuals. * = $p < 0.05$ (two-tailed t-test).

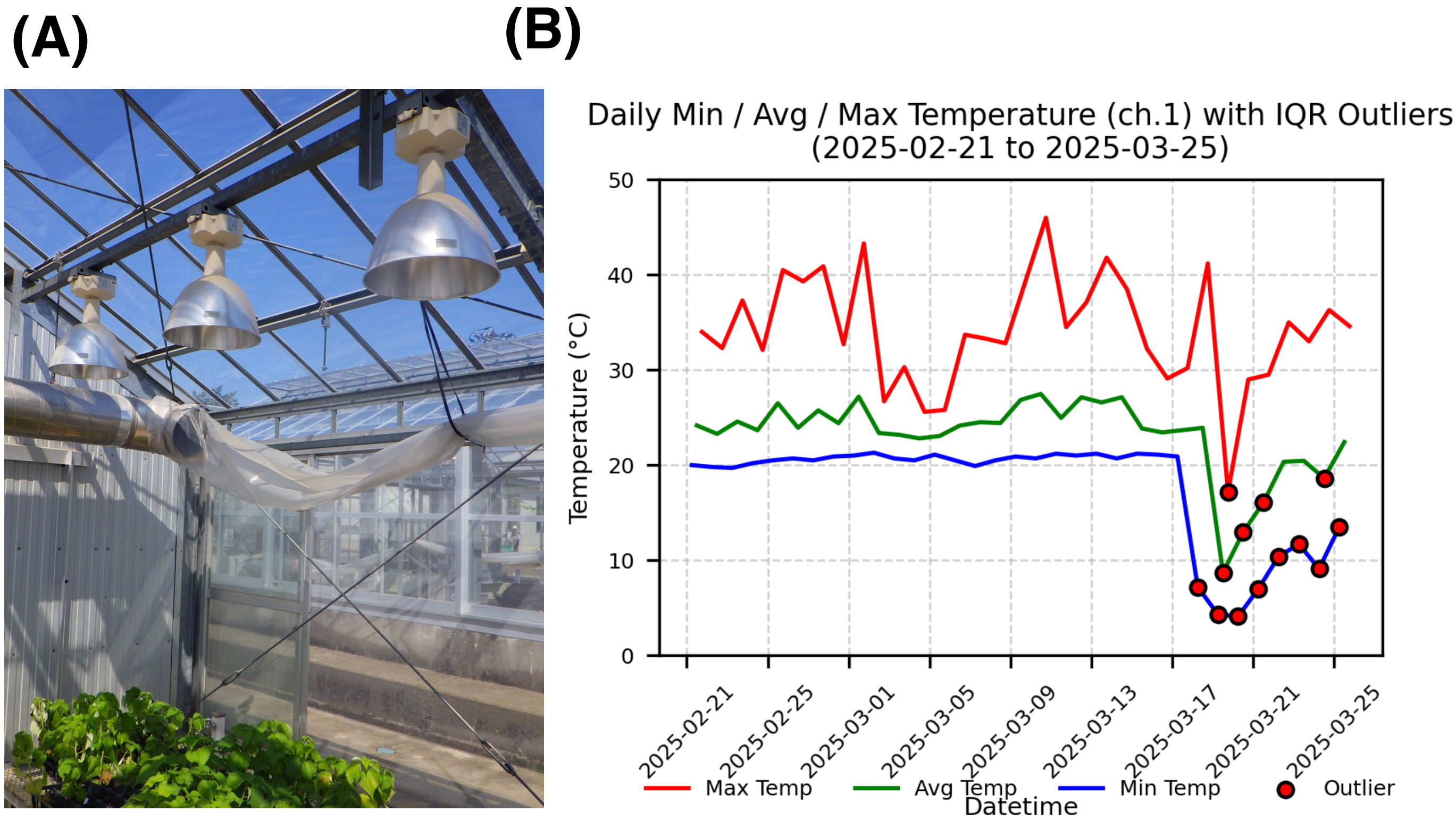

Supplemental Figure 8. Greenhouse environment during the cultivation for Fig. 5c at Ishikawa Prefectural University.

(A) Representative photograph of the supplemental lighting setup in the greenhouse at Ishikawa Prefectural University (MF250:L/BU-SC-2, Panasonic, Japan). This image illustrates the configuration used for the experiments of Fig. 2, Fig. 5c and was not taken during the 21 Feb–25 Mar 2025 period. Lamps were operated from 07:00 to 19:00 to compensate for low natural irradiance in late winter and early spring seasons. **(B)** Temperature in a green house at Ishikawa Prefectural University (from February 21 to March 25, 2025). Outliers are indicated as values exceeding the upper limit (Q3 + 1.5 × IQR) or falling below the lower limit (Q1 − 1.5 × IQR), based on the interquartile range (IQR) method.

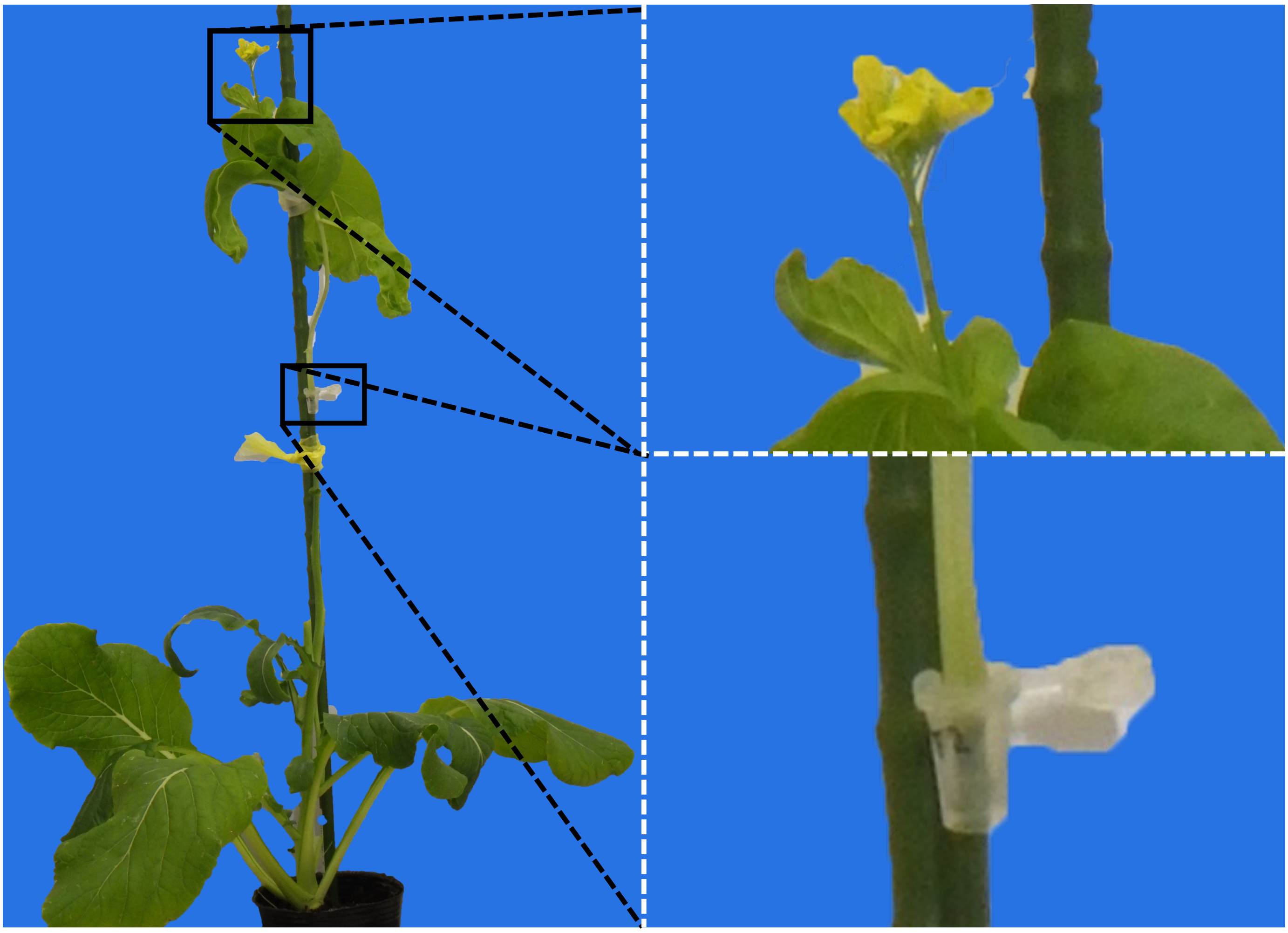

Supplemental Figure 9. Flower bud induction by grafting in the late-flowering cultivar ‘Tsukena no 2’.
A ‘Tsukena no 2’ scion, elongated by GA_3 treatment, was grafted onto a ‘CHOY’ rootstock using the splice grafting method. Flowering was observed 31 days after grafting.

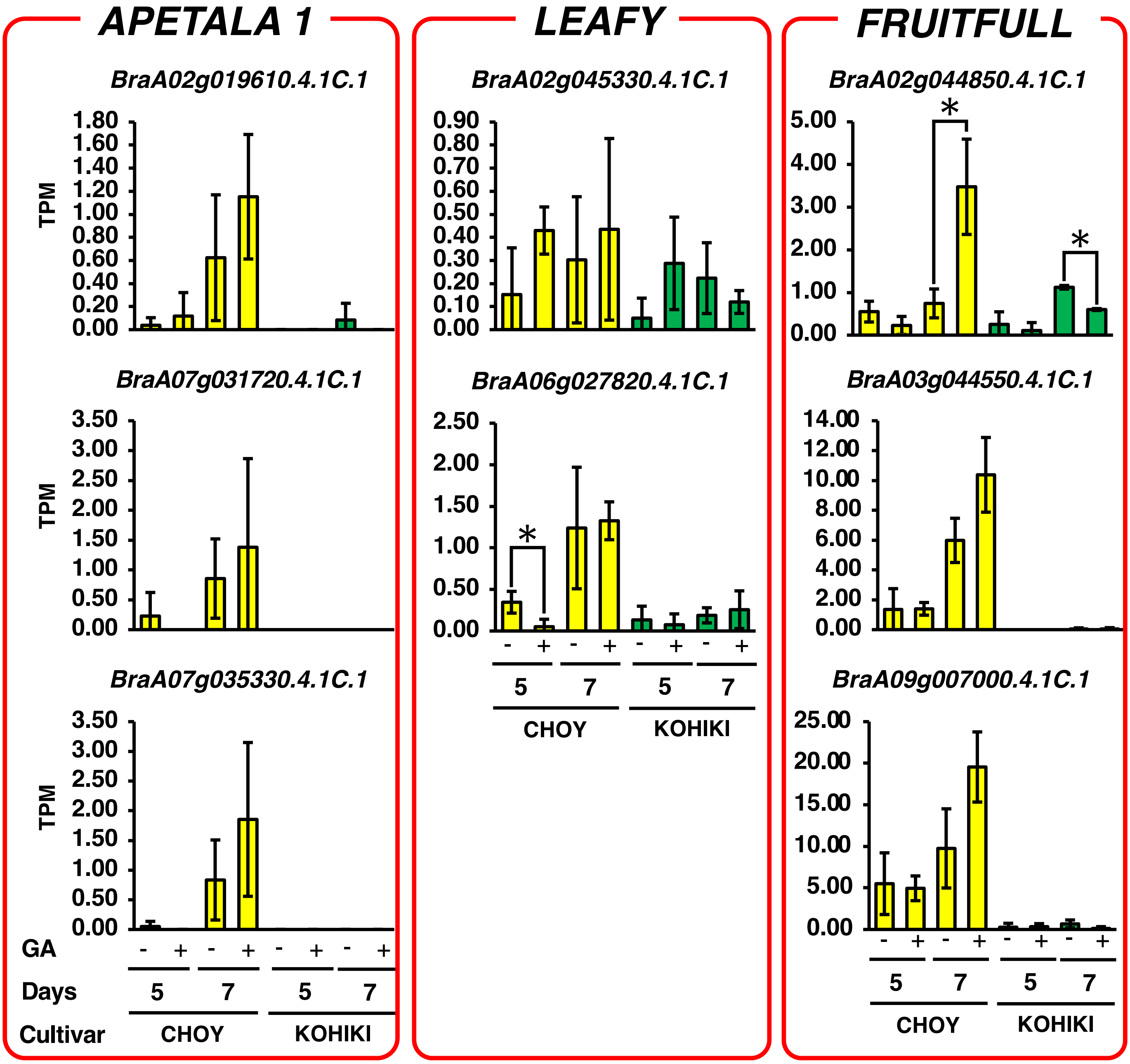

Supplemental Figure 10. Effect of GA_3 treatment on the expression of *SPL3* target genes in the shoot apex.

RNA-seq analysis was conducted using RNA extracted from the shoot apex region of ‘CHOY’ and ‘Kohiki’ at 5 and 7 days after GA_3 application. The data shown were obtained from the RNA-seq dataset presented in Fig 6. Bars and error bars represent the mean ± SD from three biological replicates. Asterisks indicate samples showing significant differences $p < 0.05$, t-test between GA-treated and untreated samples at same time point).
