# Supplementary material for: Gibberellin-mediated internode elongation for grafting and early flowering induction in Brassicaceae crops
Source: Breed Sci. 2026 Jan 16;76(2):133–43. doi: 10.1270/jsbbs.25036 (PMC13494322; doi:10.1270/jsbbs.25036)
Supplement: Supplementary file 2 [file 76_133_s2.pdf]

doi: 10.1270/jsbbs.25036

Supplemental Table 1. Summary of the Brassicaceae plants used in this study.

Culutivar	Species	Distribution source	Accession numbers
Benikururi	*R. sativus*	Matsunaga seed	-
Tsukena no 2	*B. rapa*	Kobe University	-
Yellow sarson	*B. rapa*	Kobe University	-
CHOY SUM EX CHINA3 ('CHOY')	*B. rapa*	NARO[a]	37372
Kohiki	*B. rapa*	NARO[a]	26896

NARO = National Agriculture and food Research Organization.

Supplemental Table 2. Summary of paired-end short-read RNA-seq data obtained from Illumina sequencing[a].

Sample name	Cultivar	Treatment	Replication	DDBJ bioproject accession
CHOY_CTR_5_1	*B. rapa* cv. 'CHOY'	GA-	1	DRR665501
CHOY_CTR_5_2			2	DRR665502
CHOY_CTR_5_3			3	DRR665503
CHOY_CTR_7_1			1	DRR665513
CHOY_CTR_7_2			2	DRR665514
CHOY_CTR_7_3			3	DRR665515
CHOY_GA_5_1		GA+	1	DRR665498
CHOY_GA_5_2			2	DRR665499
CHOY_GA_5_3			3	DRR665500
CHOY_GA_7_1			1	DRR665510
CHOY_GA_7_2			2	DRR665511
CHOY_GA_7_3			3	DRR665512
KOHIKI_CTR_5_1	*B. rapa* cv. 'Kohiki'	GA-	1	DRR665507
KOHIKI_CTR_5_2			2	DRR665508
KOHIKI_CTR_5_3			3	DRR665509
KOHIKI_CTR_7_1			1	DRR665519
KOHIKI_CTR_7_2			2	DRR665520
KOHIKI_CTR_7_3			3	DRR665521
KOHIKI_GA_5_1		GA+	1	DRR665504
KOHIKI_GA_5_2			2	DRR665505
KOHIKI_GA_5_3			3	DRR665506
KOHIKI_GA_7_1			1	DRR665516
KOHIKI_GA_7_2			2	DRR665517
KOHIKI_GA_7_3			3	DRR665518

[a] Average read length was 150 bp, and sequencing was performed using the NovaSeq 6000 platform.

Supplemental Table 3. Relationship between flowering-related genes known in Arabidopsis and their corresponding gene accession numbers in B. rapa.

Gene id in Arabopsisa	Gene id in *B. rapab*	Annotation for Arabidopsis
AT1G04400.1	BraA08g035830.4.1C.1, BraA10g002840.4.1C.3, BraA10g002840.4.1C.2, BraA10g002840.4.1C.1, BraA10g002840.4.1C.14, BraA10g002840.4.1C.9, BraA10g002840.4.1C.7, BraA08g035830.4.1C.3	CRY2 description:cryptochrome 2 [Source:NCBI gene (formerly Entrezgene);Acc:839529]
AT1G04400.2	BraA08g035830.4.1C.1, BraA10g002840.4.1C.3, BraA10g002840.4.1C.2, BraA10g002840.4.1C.1, BraA10g002840.4.1C.14, BraA10g002840.4.1C.9, BraA10g002840.4.1C.7, BraA08g035830.4.1C.3	CRY2 description:cryptochrome 2 [Source:NCBI gene (formerly Entrezgene);Acc:839529]
AT1G04440.1	BraA09g067850.4.1C.4, BraA09g067850.4.1C.3, BraA09g067850.4.1C.6, BraA10g002880.4.1C.1, BraA09g067850.4.1C.11, BraA02g031900.4.1C.1	CKL13 description:casein kinase like 13 [Source:NCBI gene (formerly Entrezgene);Acc:839520]
AT1G06040.1	BraA08g035090.4.1C.1, BraA10g004640.4.1C.1	STO description:B-box zinc finger family protein [Source:NCBI gene (formerly Entrezgene);Acc:837113]
AT1G06040.2	BraA08g035090.4.1C.1, BraA10g004640.4.1C.1	STO description:B-box zinc finger family protein [Source:NCBI gene (formerly Entrezgene);Acc:837113]
AT1G09570.1	BraA06g006590.4.1C.1, BraA09g065400.4.1C.3, BraA09g065400.4.1C.2, BraA09g065400.4.1C.1, BraA09g065400.4.1C.4, BraA09g065400.4.1C.5	PHYA description:phytochrome A [Source:NCBI gene (formerly Entrezgene);Acc:837483]

AT1G09570.2	BraA06g006590.4.1C.1, BraA09g065400.4.1C.5, BraA09g065400.4.1C.3, BraA09g065400.4.1C.2, BraA09g065400.4.1C.1, BraA09g065400.4.1C.4	PHYA description:phytochrome A [Source:NCBI gene (formerly Entrezgene);Acc:837483]
AT1G09570.3	BraA06g006590.4.1C.1, BraA09g065400.4.1C.3, BraA09g065400.4.1C.2, BraA09g065400.4.1C.1, BraA09g065400.4.1C.4, BraA09g065400.4.1C.5	PHYA description:phytochrome A [Source:NCBI gene (formerly Entrezgene);Acc:837483]
AT1G09570.4	BraA06g006590.4.1C.1, BraA09g065400.4.1C.3, BraA09g065400.4.1C.2, BraA09g065400.4.1C.1, BraA09g065400.4.1C.4, BraA09g065400.4.1C.5	PHYA description:phytochrome A [Source:NCBI gene (formerly Entrezgene);Acc:837483]
AT1G09570.5	BraA06g006590.4.1C.1, BraA09g065400.4.1C.3, BraA09g065400.4.1C.2, BraA09g065400.4.1C.1, BraA09g065400.4.1C.4, BraA09g065400.4.1C.5	PHYA description:phytochrome A [Source:NCBI gene (formerly Entrezgene);Acc:837483]
AT1G09570.6	BraA06g006590.4.1C.1, BraA09g065400.4.1C.3, BraA09g065400.4.1C.2, BraA09g065400.4.1C.1, BraA09g065400.4.1C.4, BraA09g065400.4.1C.5	PHYA description:phytochrome A [Source:NCBI gene (formerly Entrezgene);Acc:837483]
AT1G09700.2	BraA06g006720.4.1C.1, BraA08g033470.4.1C.1	HYL1 description:dsRNA-binding domain-like superfamily protein [Source:NCBI gene (formerly Entrezgene);Acc:837498]
AT1G13260.1	BraA06g008640.4.1C.1, BraA09g063320.4.1C.1, BraA06g041590.4.1C.1, BraA02g038200.4.1C.1	RAV1 description:uncharacterized protein [Source:NCBI gene (formerly Entrezgene);Acc:837886]
AT1G15550.1	BraA06g011750.4.1C.1, BraA09g061620.4.1C.1	GA3OX1 description:gibberellin 3-oxidase 1 [Source:NCBI gene (formerly Entrezgene);Acc:838125]
AT1G18450.1	BraA06g014410.4.1C.1, BraA08g029710.4.1C.1, BraA06g014410.4.1C.2	ARP4 description:actin-related protein 4 [Source:NCBI gene (formerly Entrezgene);Acc:838425]

AT1G22690.1	BraA09g043310.4.1C.1	AT1G22690 description:Gibberellin-regulated family protein [Source:NCBI gene (formerly Entrezgene);Acc:838875]
AT1G22690.2	BraA09g043310.4.1C.1	AT1G22690 description:Gibberellin-regulated family protein [Source:NCBI gene (formerly Entrezgene);Acc:838875]
AT1G22690.3	BraA09g043310.4.1C.1	AT1G22690 description:Gibberellin-regulated family protein [Source:NCBI gene (formerly Entrezgene);Acc:838875]
AT1G22770.1	BraA09g043240.4.1C.4, BraA09g043240.4.1C.3, BraA09g043240.4.1C.1, BraA09g043240.4.1C.2	GI description:gigantea protein (GI) [Source:NCBI gene (formerly Entrezgene);Acc:838883]
AT1G24260.1	BraA09g040010.4.1C.1, BraA07g012910.4.1C.1	SEP3 description:K-box region and MADS-box transcription factor family protein [Source:NCBI gene (formerly Entrezgene);Acc:839040]
AT1G24260.2	BraA09g040010.4.1C.1, BraA07g012910.4.1C.1	SEP3 description:K-box region and MADS-box transcription factor family protein [Source:NCBI gene (formerly Entrezgene);Acc:839040]
AT1G25540.1	BraA08g026420.4.1C.1, BraA08g026420.4.1C.4, BraA09g040900.4.1C.1	PFT1 description:phytochrome and flowering time regulatory protein (PFT1) [Source:NCBI gene (formerly Entrezgene);Acc:839141]
AT1G25540.2	BraA08g026420.4.1C.1, BraA08g026420.4.1C.4, BraA09g040900.4.1C.1	PFT1 description:phytochrome and flowering time regulatory protein (PFT1) [Source:NCBI gene (formerly Entrezgene);Acc:839141]
AT1G26310.1	BraA02g019610.4.1C.1, BraA07g031720.4.1C.1	CAL description:K-box region and MADS-box transcription factor family protein [Source:NCBI gene (formerly Entrezgene);Acc:839172]
AT1G30040.1	BraA07g011190.4.1C.1, BraA09g037690.4.1C.1, BraA08g024370.4.1C.1	GA2OX2 description:gibberellin 2-oxidase [Source:NCBI gene (formerly Entrezgene);Acc:839883]
AT1G30040.2	BraA07g011190.4.1C.1, BraA09g037690.4.1C.1, BraA08g024370.4.1C.1	GA2OX2 description:gibberellin 2-oxidase [Source:NCBI gene (formerly Entrezgene);Acc:839883]

AT1G30950.1	BraA08g011000.4.1C.1	UFO description:F-box family protein [Source:NCBI gene (formerly Entrezgene);Acc:839982]
AT1G30960.1	BraA09g036550.4.1C.1	AT1G30960 description:GTP-binding family protein [Source:NCBI gene (formerly Entrezgene);Acc:839983]
AT1G44090.1	BraA08g006190.4.1C.1	GA20OX5 description:gibberellin 20-oxidase 5 [Source:NCBI gene (formerly Entrezgene);Acc:841012]
AT1G44446.1	BraA10g008030.4.1C.1, BraA10g008030.4.1C.8, BraA10g008030.4.1C.6	CH1 description:Pheophorbide a oxygenase family protein with Rieske 2Fe-2S domain-containing protein [Source:NCBI gene (formerly Entrezgene);Acc:841029]
AT1G44446.2	BraA10g008030.4.1C.1	CH1 description:Pheophorbide a oxygenase family protein with Rieske 2Fe-2S domain-containing protein [Source:NCBI gene (formerly Entrezgene);Acc:841029]
AT1G44446.3	BraA10g008030.4.1C.2, BraA10g008030.4.1C.1	CH1 description:Pheophorbide a oxygenase family protein with Rieske 2Fe-2S domain-containing protein [Source:NCBI gene (formerly Entrezgene);Acc:841029]
AT1G44446.4	BraA10g008030.4.1C.8, BraA10g008030.4.1C.6, BraA10g008030.4.1C.1	CH1 description:Pheophorbide a oxygenase family protein with Rieske 2Fe-2S domain-containing protein [Source:NCBI gene (formerly Entrezgene);Acc:841029]
AT1G48270.1	BraA06g004750.4.1C.1	GCR1 description:G-protein-coupled receptor 1 [Source:NCBI gene (formerly Entrezgene);Acc:841247]
AT1G48270.2	BraA06g004750.4.1C.1	GCR1 description:G-protein-coupled receptor 1 [Source:NCBI gene (formerly Entrezgene);Acc:841247]

AT1G49480.1	BraA08g003890.4.1C.1, BraA05g020650.4.1C.1	RTV1 description:uncharacterized protein [Source:NCBI gene (formerly Entrezgene);Acc:841372]
AT1G49480.2	BraA08g003890.4.1C.1, BraA05g020650.4.1C.1	RTV1 description:uncharacterized protein [Source:NCBI gene (formerly Entrezgene);Acc:841372]
AT1G49480.3	BraA08g003890.4.1C.1, BraA05g020650.4.1C.1	RTV1 description:uncharacterized protein [Source:NCBI gene (formerly Entrezgene);Acc:841372]
AT1G50960.1	BraA05g019350.4.1C.1	GA2OX7 description:gibberellin 2-oxidase 7 [Source:NCBI gene (formerly Entrezgene);Acc:841518]
AT1G52800.1	BraA06g001870.4.1C.1	AT1G52800 description:2-oxoglutarate (2OG) and Fe(II)-dependent oxygenase superfamily protein [Source:NCBI gene (formerly Entrezgene);Acc:841713]
AT1G53160.1	BraA06g001570.4.1C.1	SPL4 description:squamosa promoter binding protein-like 4 [Source:NCBI gene (formerly Entrezgene);Acc:841749]
AT1G53160.2	BraA06g001570.4.1C.1	SPL4 description:squamosa promoter binding protein-like 4 [Source:NCBI gene (formerly Entrezgene);Acc:841749]
AT1G53160.3	BraA06g001570.4.1C.1	SPL4 description:squamosa promoter binding protein-like 4 [Source:NCBI gene (formerly Entrezgene);Acc:841749]
AT1G57820.1	BraA07g032440.4.1C.1	VIM1 description:Zinc finger (C3HC4-type RING finger) family protein [Source:NCBI gene (formerly Entrezgene);Acc:842157]
AT1G57820.2	BraA07g032440.4.1C.1	VIM1 description:Zinc finger (C3HC4-type RING finger) family protein [Source:NCBI gene (formerly Entrezgene);Acc:842157]
AT1G57820.3	BraA07g032440.4.1C.1	VIM1 description:Zinc finger (C3HC4-type RING finger) family protein [Source:NCBI gene (formerly Entrezgene);Acc:842157]

AT1G60980.1	BraA10g030280.4.1C.1	GA20OX4 description:gibberellin 20-oxidase 4 [Source:NCBI gene (formerly Entrezgene);Acc:842389]
AT1G61040.1	BraA01g029970.4.1C.3, BraA01g029970.4.1C.2, BraA01g029970.4.1C.1	VIP5 description:plus-3 domain-containing protein [Source:NCBI gene (formerly Entrezgene);Acc:842395]
AT1G61040.2	BraA01g029970.4.1C.3, BraA01g029970.4.1C.2, BraA01g029970.4.1C.1	VIP5 description:plus-3 domain-containing protein [Source:NCBI gene (formerly Entrezgene);Acc:842395]
AT1G61040.3	BraA01g029970.4.1C.3, BraA01g029970.4.1C.2, BraA01g029970.4.1C.1	VIP5 description:plus-3 domain-containing protein [Source:NCBI gene (formerly Entrezgene);Acc:842395]
AT1G62750.1	BraA09g016210.4.1C.1, BraA09g016210.4.1C.3, BraA09g016210.4.1C.4, BraA09g016210.4.1C.2	SCO1 description:Translation elongation factor EFG/EF2 protein [Source:NCBI gene (formerly Entrezgene);Acc:842573]
AT1G65480.1	BraA02g017210.4.1C.1, BraA07g032840.4.1C.1	FT description:PEBP (phosphatidylethanolamine-binding protein) family protein [Source:NCBI gene (formerly Entrezgene);Acc:842859]
AT1G66350.1	BraA02g017570.4.1C.1	RGL1 description:RGA-like 1 [Source:NCBI gene (formerly Entrezgene);Acc:842953]
AT1G69120.1	BraA07g031720.4.1C.1, BraA02g019610.4.1C.1	AP1 description:K-box region and MADS-box transcription factor family protein [Source:NCBI gene (formerly Entrezgene);Acc:843244]
AT1G69120.2	BraA02g019610.4.1C.1, BraA07g031720.4.1C.1	AP1 description:K-box region and MADS-box transcription factor family protein [Source:NCBI gene (formerly Entrezgene);Acc:843244]
AT1G69120.3	BraA07g035330.4.1C.1, BraA07g031720.4.1C.1, BraA02g019610.4.1C.1	AP1 description:K-box region and MADS-box transcription factor family protein [Source:NCBI gene (formerly Entrezgene);Acc:843244]
AT1G70170.1	BraA07g031290.4.1C.1, BraA07g036310.4.1C.1	MMP description:matrix metalloproteinase [Source:NCBI gene (formerly Entrezgene);Acc:843353]

AT1G71692.1	BraA02g021110.4.1C.1	AGL12 description:AGAMOUS-like 12 [Source:NCBI gene (formerly Entrezgene);Acc:843497]
AT1G72830.1	BraA07g030260.4.1C.1, BraA07g038390.4.1C.1, BraA02g022130.4.1C.2, BraA02g022130.4.1C.1	NF-YA3 description:nuclear factor Y, subunit A3 [Source:NCBI gene (formerly Entrezgene);Acc:843614]
AT1G72830.2	BraA07g030260.4.1C.1, BraA07g038390.4.1C.1, BraA02g022130.4.1C.2, BraA02g022130.4.1C.1	NF-YA3 description:nuclear factor Y, subunit A3 [Source:NCBI gene (formerly Entrezgene);Acc:843614]
AT1G74660.1	BraA07g029420.4.1C.1	MIF1 description:mini zinc finger 1 [Source:NCBI gene (formerly Entrezgene);Acc:843805]
AT1G74660.2	BraA07g029420.4.1C.1	MIF1 description:mini zinc finger 1 [Source:NCBI gene (formerly Entrezgene);Acc:843805]
AT1G74670.1	BraA02g023310.4.1C.1	GASA6 description:Gibberellin-regulated family protein [Source:NCBI gene (formerly Entrezgene);Acc:843806]
AT1G77080.11	BraA02g047130.4.1C.1	MAF1 description:K-box region and MADS-box transcription factor family protein [Source:NCBI gene (formerly Entrezgene);Acc:844042]
AT1G77080.5	BraA02g047130.4.1C.1	MAF1 description:K-box region and MADS-box transcription factor family protein [Source:NCBI gene (formerly Entrezgene);Acc:844042]
AT1G77080.7	BraA02g047100.4.1C.1	MAF1 description:K-box region and MADS-box transcription factor family protein [Source:NCBI gene (formerly Entrezgene);Acc:844042]
AT1G78440.1	BraA02g025590.4.1C.1, BraA07g042710.4.1C.1, BraA07g042700.4.1C.1	ATGA2OX1 description:gibberellin 2-beta-dioxygenase [Source:NCBI gene (formerly Entrezgene);Acc:844180]
AT1G79460.1	BraA07g043540.4.1C.2, BraA07g043540.4.1C.1	GA2 description:Terpenoid cyclases/Protein prenyltransferases superfamily protein [Source:NCBI gene (formerly Entrezgene);Acc:844284]

AT1G79730.1	BraA10g033440.4.1C.1	ELF7 description:hydroxyproline-rich glycoprotein family protein [Source:NCBI gene (formerly Entrezgene);Acc:844312]
AT1G80330.1	BraA02g026990.4.1C.1	GA3OX4 description:gibberellin 3-oxidase 4 [Source:NCBI gene (formerly Entrezgene);Acc:844373]
AT1G80340.1	BraA02g026980.4.1C.1	GA3OX2 description:gibberellin 3-oxidase 2 [Source:NCBI gene (formerly Entrezgene);Acc:844374]
AT1G80680.1	BraA07g027070.4.1C.1	SAR3 description:SUPPRESSOR OF AUXIN RESISTANCE 3 [Source:NCBI gene (formerly Entrezgene);Acc:844407]
AT2G02560.1	BraA02g037160.4.1C.1	CAND1 description:cullin-associated and neddylation dissociated [Source:NCBI gene (formerly Entrezgene);Acc:814786]
AT2G02560.2	BraA02g037160.4.1C.1	CAND1 description:cullin-associated and neddylation dissociated [Source:NCBI gene (formerly Entrezgene);Acc:814786]
AT2G03710.1	BraA06g041990.4.1C.1	SEP4 description:K-box region and MADS-box transcription factor family protein [Source:NCBI gene (formerly Entrezgene);Acc:814898]
AT2G03710.2	BraA06g041990.4.1C.1	SEP4 description:K-box region and MADS-box transcription factor family protein [Source:NCBI gene (formerly Entrezgene);Acc:814898]
AT2G03710.3	BraA06g041990.4.1C.1, BraA02g037660.4.1C.1	SEP4 description:K-box region and MADS-box transcription factor family protein [Source:NCBI gene (formerly Entrezgene);Acc:814898]
AT2G04030.1	BraA06g041790.4.1C.1, BraA06g041790.4.1C.21, BraA06g041790.4.1C.28, BraA06g041790.4.1C.5, BraA09g025360.4.1C.3, BraA06g041790.4.1C.16, BraA06g041790.4.1C.12, BraA06g041790.4.1C.31, BraA06g041790.4.1C.4, BraA06g041790.4.1C.2	CR88 description:Chaperone protein htpG family protein [Source:NCBI gene (formerly Entrezgene);Acc:814930]

AT2G04030.2	BraA06g041790.4.1C.1, BraA06g041790.4.1C.21, BraA06g041790.4.1C.28, BraA06g041790.4.1C.5, BraA09g025360.4.1C.3, BraA06g041790.4.1C.16, BraA06g041790.4.1C.12, BraA06g041790.4.1C.31, BraA06g041790.4.1C.4, BraA06g041790.4.1C.2	CR88 description:Chaperone protein htpG family protein [Source:NCBI gene (formerly Entrezgene);Acc:814930]
AT2G18790.1	BraA05g032610.4.1C.1, BraA05g032610.4.1C.3, BraA05g032610.4.1C.4	PHYB description:phytochrome B [Source:NCBI gene (formerly Entrezgene);Acc:816394]
AT2G18790.2	BraA05g032610.4.1C.3, BraA05g032610.4.1C.1, BraA05g032610.4.1C.4	PHYB description:phytochrome B [Source:NCBI gene (formerly Entrezgene);Acc:816394]
AT2G18915.1	BraA07g001820.4.1C.1, BraA07g001840.4.1C.1, BraA07g001810.4.1C.1, BraA07g001810.4.1C.3, BraA07g001810.4.1C.2	LKP2 description:LOV KELCH protein 2 [Source:NCBI gene (formerly Entrezgene);Acc:816408]
AT2G18915.2	BraA07g001820.4.1C.1, BraA07g001840.4.1C.1, BraA07g001810.4.1C.1, BraA07g001810.4.1C.3, BraA07g001810.4.1C.2	LKP2 description:LOV KELCH protein 2 [Source:NCBI gene (formerly Entrezgene);Acc:816408]
AT2G19520.1	BraA07g001270.4.1C.4, BraA07g001270.4.1C.3, BraA07g001270.4.1C.2, BraA07g001270.4.1C.1, BraA09g012420.4.1C.1, BraA09g059470.4.1C.1, BraA07g001270.4.1C.6	FVE description:Transducin family protein / WD-40 repeat family protein [Source:NCBI gene (formerly Entrezgene);Acc:816471]
AT2G22540.1	BraA04g018360.4.1C.1, BraA09g057670.4.1C.1	SVP description:K-box region and MADS-box transcription factor family protein [Source:NCBI gene (formerly Entrezgene);Acc:816787]
AT2G22540.2	BraA04g018360.4.1C.1, BraA09g057670.4.1C.1	SVP description:K-box region and MADS-box transcription factor family protein [Source:NCBI gene (formerly Entrezgene);Acc:816787]

AT2G22540.3	BraA04g018360.4.1C.1, BraA09g057670.4.1C.1	SVP description:K-box region and MADS-box transcription factor family protein [Source:NCBI gene (formerly Entrezgene);Acc:816787]
AT2G22630.1	BraA09g057610.4.1C.1, BraA04g018420.4.1C.1, BraA01g001170.4.1C.1	AGL17 description:AGAMOUS-like 17 [Source:NCBI gene (formerly Entrezgene);Acc:816794]
AT2G22630.2	BraA09g057610.4.1C.1, BraA04g018420.4.1C.1, BraA01g001170.4.1C.1	AGL17 description:AGAMOUS-like 17 [Source:NCBI gene (formerly Entrezgene);Acc:816794]
AT2G23380.1	BraA04g019050.4.1C.1	CLF description:SET domain-containing protein [Source:NCBI gene (formerly Entrezgene);Acc:816870]
AT2G23380.2	BraA04g019050.4.1C.1	CLF description:SET domain-containing protein [Source:NCBI gene (formerly Entrezgene);Acc:816870]
AT2G26710.1	BraA07g018650.4.1C.1	BAS1 description:Cytochrome P450 superfamily protein [Source:NCBI gene (formerly Entrezgene);Acc:817212]
AT2G27550.1	BraA03g025520.4.1C.1, BraA04g021950.4.1C.1, BraA04g021830.4.1C.1, BraA07g019110.4.1C.1	ATC description:centroradiali [Source:NCBI gene (formerly Entrezgene);Acc:817302]
AT2G28550.1	BraA07g019860.4.1C.1	RAP2.7 description:uncharacterized protein [Source:NCBI gene (formerly Entrezgene);Acc:817403]
AT2G28550.5	BraA07g019860.4.1C.6, BraA07g019860.4.1C.1, BraA07g019860.4.1C.2	RAP2.7 description:uncharacterized protein [Source:NCBI gene (formerly Entrezgene);Acc:817403]
AT2G32950.1	BraA05g012250.4.1C.1, BraA05g012250.4.1C.3	COP1 description:Transducin/WD40 repeat-like superfamily protein [Source:NCBI gene (formerly Entrezgene);Acc:817857]
AT2G32950.2	BraA05g012250.4.1C.1, BraA05g012250.4.1C.3	COP1 description:Transducin/WD40 repeat-like superfamily protein [Source:NCBI gene (formerly Entrezgene);Acc:817857]

AT2G33810.1	BraA05g011410.4.1C.1, BraA03g018150.4.1C.1	SPL3 description:squamosa promoter binding protein-like 3 [Source:NCBI gene (formerly Entrezgene);Acc:817948]
AT2G34555.1	BraA05g010790.4.1C.1	ATGA2OX3 description:gibberellin 2-oxidase 3 [Source:NCBI gene (formerly Entrezgene);Acc:818019]
AT2G37678.1	BraA05g007990.4.1C.4, BraA05g007990.4.1C.3, BraA05g007990.4.1C.2	FHY1 description:far-red elongated hypocotyl 1 [Source:NCBI gene (formerly Entrezgene);Acc:5007942]
AT2G37678.2	BraA05g007990.4.1C.4, BraA05g007990.4.1C.3, BraA05g007990.4.1C.2, BraA05g007990.4.1C.1	FHY1 description:far-red elongated hypocotyl 1 [Source:NCBI gene (formerly Entrezgene);Acc:5007942]
AT2G39810.1	BraA04g030190.4.1C.1, BraA04g030190.4.1C.4, BraA04g030190.4.1C.2	HOS1 description:ubiquitin-protein ligase [Source:NCBI gene (formerly Entrezgene);Acc:818568]
AT2G39810.2	BraA04g030190.4.1C.1, BraA04g030190.4.1C.4, BraA04g030190.4.1C.3	HOS1 description:ubiquitin-protein ligase [Source:NCBI gene (formerly Entrezgene);Acc:818568]
AT2G40080.1	BraA04g030340.4.1C.1	ELF4 description:EARLY FLOWERING-like protein (DUF1313) [Source:NCBI gene (formerly Entrezgene);Acc:818596]
AT2G44680.1	BraA03g023800.4.1C.2, BraA03g023800.4.1C.1	CKB4 description:casein kinase II beta subunit 4 [Source:NCBI gene (formerly Entrezgene);Acc:819076]
AT2G44680.2	BraA03g023800.4.1C.2, BraA03g023800.4.1C.1	CKB4 description:casein kinase II beta subunit 4 [Source:NCBI gene (formerly Entrezgene);Acc:819076]
AT2G44680.3	BraA03g023800.4.1C.1, BraA03g023800.4.1C.2	CKB4 description:casein kinase II beta subunit 4 [Source:NCBI gene (formerly Entrezgene);Acc:819076]
AT2G45660.1	BraA05g005530.4.1C.1, BraA03g024180.4.1C.1, BraA04g033890.4.1C.2, BraA04g033890.4.1C.1	AGL20 description:AGAMOUS-like 20 [Source:NCBI gene (formerly Entrezgene);Acc:819174]

AT2G45660.2	BraA05g005530.4.1C.1, BraA04g033890.4.1C.2, BraA04g033890.4.1C.1, BraA03g024180.4.1C.1	AGL20 description:AGAMOUS-like 20 [Source:NCBI gene (formerly Entrezgene);Acc:819174]
AT2G46340.1	BraA05g001610.4.1C.7, BraA05g001610.4.1C.1	SPA1 description:SPA (suppressor of phyA-105) protein family [Source:NCBI gene (formerly Entrezgene);Acc:819242]
AT2G46340.2	BraA05g001610.4.1C.7, BraA05g001610.4.1C.1	SPA1 description:SPA (suppressor of phyA-105) protein family [Source:NCBI gene (formerly Entrezgene);Acc:819242]
AT2G47700.1	BraA05g000400.4.1C.3, BraA05g000400.4.1C.2	RFI2 description:RING/U-box superfamily protein [Source:NCBI gene (formerly Entrezgene);Acc:819383]
AT2G47700.2	BraA05g000400.4.1C.3, BraA05g000400.4.1C.2	RFI2 description:RING/U-box superfamily protein [Source:NCBI gene (formerly Entrezgene);Acc:819383]
AT3G01460.1	BraA01g046690.4.1C.2, BraA01g046690.4.1C.5	MBD9 description:methyl-CPG-binding domain 9 [Source:NCBI gene (formerly Entrezgene);Acc:821132]
AT3G02310.1	BraA05g044380.4.1C.1	SEP2 description:K-box region and MADS-box transcription factor family protein [Source:NCBI gene (formerly Entrezgene);Acc:821151]
AT3G02310.2	BraA05g044380.4.1C.1	SEP2 description:K-box region and MADS-box transcription factor family protein [Source:NCBI gene (formerly Entrezgene);Acc:821151]
AT3G03090.1	BraA05g043930.4.1C.1, BraA01g045700.4.1C.1, BraA05g043930.4.1C.4, BraA01g045700.4.1C.5, BraA05g043930.4.1C.3, BraA02g006950.4.1C.6, BraA02g006950.4.1C.2, BraA02g006950.4.1C.1	VGT1 description:vacuolar glucose transporter 1 [Source:NCBI gene (formerly Entrezgene);Acc:821117]
AT3G04510.1	BraA03g032280.4.1C.1, BraA01g044930.4.1C.1, BraA09g004870.4.1C.1	LSH2 description:LIGHT-DEPENDENT SHORT HYPOCOTYLS-like protein (DUF640) [Source:NCBI gene (formerly Entrezgene);Acc:819607]

AT3G04610.1	BraA03g032320.4.1C.5, BraA03g032320.4.1C.1	FLK description:RNA-binding KH domain-containing protein [Source:NCBI gene (formerly Entrezgene);Acc:819619]
AT3G04610.2	BraA03g032320.4.1C.5, BraA03g032320.4.1C.1	FLK description:RNA-binding KH domain-containing protein [Source:NCBI gene (formerly Entrezgene);Acc:819619]
AT3G04610.3	BraA03g032320.4.1C.5, BraA03g032320.4.1C.1	FLK description:RNA-binding KH domain-containing protein [Source:NCBI gene (formerly Entrezgene);Acc:819619]
AT3G04610.4	BraA03g032320.4.1C.5, BraA03g032320.4.1C.1	FLK description:RNA-binding KH domain-containing protein [Source:NCBI gene (formerly Entrezgene);Acc:819619]
AT3G05040.1	BraA06g036900.4.1C.2, BraA06g036900.4.1C.1	HST description:ARM repeat superfamily protein [Source:NCBI gene (formerly Entrezgene);Acc:819666]
AT3G05040.2	BraA06g036900.4.1C.2, BraA06g036900.4.1C.1	HST description:ARM repeat superfamily protein [Source:NCBI gene (formerly Entrezgene);Acc:819666]
AT3G05120.1	BraA06g036840.4.1C.1, BraA06g036840.4.1C.2	GID1A description:alpha/beta-Hydrolases superfamily protein [Source:NCBI gene (formerly Entrezgene);Acc:819674]
AT3G05690.1	BraA03g032920.4.1C.4, BraA03g032920.4.1C.2, BraA03g032920.4.1C.1	NF-YA2 description:nuclear factor Y, subunit A2 [Source:NCBI gene (formerly Entrezgene);Acc:819738]
AT3G05690.2	BraA03g032920.4.1C.4, BraA03g032920.4.1C.2, BraA03g032920.4.1C.1	NF-YA2 description:nuclear factor Y, subunit A2 [Source:NCBI gene (formerly Entrezgene);Acc:819738]
AT3G06930.1	BraA05g041010.4.1C.1, BraA02g041890.4.1C.1	PRMT4B description:protein arginine methyltransferase 4B [Source:NCBI gene (formerly Entrezgene);Acc:819878]
AT3G06930.2	BraA05g041010.4.1C.1, BraA02g041890.4.1C.1	PRMT4B description:protein arginine methyltransferase 4B [Source:NCBI gene (formerly Entrezgene);Acc:819878]

AT3G07650.1	BraA01g043190.4.1C.4, BraA01g043190.4.1C.2, BraA01g043190.4.1C.3, BraA01g043190.4.1C.1, BraA05g040400.4.1C.3, BraA05g040400.4.1C.1	COL9 description:CONSTANS-like 9 [Source:NCBI gene (formerly Entrezgene);Acc:819956]
AT3G07650.2	BraA01g043190.4.1C.4, BraA01g043190.4.1C.2, BraA01g043190.4.1C.3, BraA01g043190.4.1C.1, BraA05g040400.4.1C.3, BraA05g040400.4.1C.1	COL9 description:CONSTANS-like 9 [Source:NCBI gene (formerly Entrezgene);Acc:819956]
AT3G07650.3	BraA01g043190.4.1C.4, BraA01g043190.4.1C.2, BraA01g043190.4.1C.3, BraA01g043190.4.1C.1, BraA05g040400.4.1C.3, BraA05g040400.4.1C.1	COL9 description:CONSTANS-like 9 [Source:NCBI gene (formerly Entrezgene);Acc:819956]
AT3G07650.4	BraA01g043190.4.1C.4, BraA01g043190.4.1C.2, BraA01g043190.4.1C.3, BraA01g043190.4.1C.1, BraA05g040400.4.1C.3, BraA05g040400.4.1C.1	COL9 description:CONSTANS-like 9 [Source:NCBI gene (formerly Entrezgene);Acc:819956]
AT3G10390.1	BraA03g035050.4.1C.1	FLD description:protein FLOWERING locus D-like protein [Source:NCBI gene (formerly Entrezgene);Acc:820202]
AT3G10390.2	BraA03g035050.4.1C.1	FLD description:protein FLOWERING locus D-like protein [Source:NCBI gene (formerly Entrezgene);Acc:820202]
AT3G10390.3	BraA03g035050.4.1C.1	FLD description:protein FLOWERING locus D-like protein [Source:NCBI gene (formerly Entrezgene);Acc:820202]
AT3G10390.4	BraA03g035050.4.1C.1	FLD description:protein FLOWERING locus D-like protein [Source:NCBI gene (formerly Entrezgene);Acc:820202]
AT3G11440.2	BraA05g037780.4.1C.3, BraA05g037780.4.1C.2, BraA05g037780.4.1C.1	MYB65 description:myb domain protein 65 [Source:NCBI gene (formerly Entrezgene);Acc:820317]

AT3G11540.1	BraA05g037670.4.1C.1, BraA05g037670.4.1C.3, BraA03g035590.4.1C.7, BraA03g035590.4.1C.2, BraA03g035590.4.1C.1, BraA03g035590.4.1C.4	SPY description:Tetratricopeptide repeat (TPR)-like superfamily protein [Source:NCBI gene (formerly Entrezgene);Acc:820327]
AT3G13682.1	BraA01g039720.4.1C.1	LDL2 description:LSD1-like2 [Source:NCBI gene (formerly Entrezgene);Acc:820577]
AT3G15270.1	BraA05g034230.4.1C.1, BraA01g038640.4.1C.1, BraA05g018010.4.1C.1	SPL5 description:squamosa promoter binding protein-like 5 [Source:NCBI gene (formerly Entrezgene);Acc:820758]
AT3G18990.1	BraA01g035780.4.1C.1, BraA05g030510.4.1C.1	VRN1 description:AP2/B3-like transcriptional factor family protein [Source:NCBI gene (formerly Entrezgene);Acc:821432]
AT3G18990.2	BraA01g035780.4.1C.1, BraA05g030510.4.1C.1	VRN1 description:AP2/B3-like transcriptional factor family protein [Source:NCBI gene (formerly Entrezgene);Acc:821432]
AT3G19980.1	BraA03g039780.4.1C.1, BraA05g029560.4.1C.2, BraA05g029560.4.1C.1, BraA01g035030.4.1C.1, BraA03g039780.4.1C.2, BraA08g003200.4.1C.6, BraA08g003200.4.1C.2, BraA08g003200.4.1C.3, BraA08g003200.4.1C.5	FYPP3 description:flower-specific, phytochrome-associated protein phosphatase 3 [Source:NCBI gene (formerly Entrezgene);Acc:821536]
AT3G20550.1	BraA03g040010.4.1C.2, BraA03g040010.4.1C.1	DDL description:SMAD/FHA domain-containing protein [Source:NCBI gene (formerly Entrezgene);Acc:821601]
AT3G20740.1	BraA01g034560.4.1C.1	FIE description:Transducin/WD40 repeat-like superfamily protein [Source:NCBI gene (formerly Entrezgene);Acc:821622]
AT3G21320.1	BraA05g027820.4.1C.1	AT3G21320 description:EARLY FLOWERING protein [Source:NCBI gene (formerly Entrezgene);Acc:821686]

AT3G22380.1	BraA01g033220.4.1C.22, BraA01g033220.4.1C.18, BraA01g033220.4.1C.5, BraA01g033220.4.1C.4, BraA01g033220.4.1C.2, BraA01g033220.4.1C.1, BraA01g033220.4.1C.19, BraA01g033220.4.1C.16, BraA01g033220.4.1C.15, BraA01g033220.4.1C.11, BraA01g033220.4.1C.8, BraA01g033220.4.1C.7, BraA01g033220.4.1C.3, BraA01g033220.4.1C.20	TIC description:time for coffee [Source:NCBI gene (formerly Entrezgene);Acc:821807]
AT3G22380.2	BraA01g033220.4.1C.22, BraA01g033220.4.1C.18, BraA01g033220.4.1C.5, BraA01g033220.4.1C.4, BraA01g033220.4.1C.2, BraA01g033220.4.1C.1, BraA01g033220.4.1C.19, BraA01g033220.4.1C.16, BraA01g033220.4.1C.15, BraA01g033220.4.1C.11, BraA01g033220.4.1C.8, BraA01g033220.4.1C.7, BraA01g033220.4.1C.3, BraA01g033220.4.1C.20	TIC description:time for coffee [Source:NCBI gene (formerly Entrezgene);Acc:821807]
AT3G22380.3	BraA01g033220.4.1C.22, BraA01g033220.4.1C.18, BraA01g033220.4.1C.5, BraA01g033220.4.1C.4, BraA01g033220.4.1C.2, BraA01g033220.4.1C.1, BraA01g033220.4.1C.19, BraA01g033220.4.1C.16, BraA01g033220.4.1C.15, BraA01g033220.4.1C.11, BraA01g033220.4.1C.8, BraA01g033220.4.1C.7, BraA01g033220.4.1C.3, BraA01g033220.4.1C.20	TIC description:time for coffee [Source:NCBI gene (formerly Entrezgene);Acc:821807]
AT3G24440.1	BraA07g009000.4.1C.1	VRN5 description:Fibronectin type III domain-containing protein [Source:NCBI gene (formerly Entrezgene);Acc:822034]

AT3G24440.2	BraA07g009000.4.1C.1	VRN5 description:Fibronectin type III domain-containing protein [Source:NCBI gene (formerly Entrezgene);Acc:822034]
AT3G26120.1	BraA02g038420.4.1C.1	TEL1 description:terminal EAR1-like 1 [Source:NCBI gene (formerly Entrezgene);Acc:822211]
AT3G26640.1	BraA09g063610.4.1C.1	LWD2 description:Transducin/WD40 repeat-like superfamily protein [Source:NCBI gene (formerly Entrezgene);Acc:822276]
AT3G30180.1	BraA02g040900.4.1C.1, BraA06g038980.4.1C.1, BraA09g004190.4.1C.1, BraA04g016660.4.1C.1	BR6OX2 description:brassinosteroid-6-oxidase 2 [Source:NCBI gene (formerly Entrezgene);Acc:822709]
AT3G33520.1	BraA08g000140.4.1C.1	ARP6 description:actin-related protein 6 [Source:NCBI gene (formerly Entrezgene);Acc:823150]
AT3G46640.3	BraA06g021010.4.1C.4, BraA06g021010.4.1C.3, BraA06g021010.4.1C.2, BraA06g021010.4.1C.1	PCL1 description:Homeodomain-like superfamily protein [Source:NCBI gene (formerly Entrezgene);Acc:823817]
AT3G54720.1	BraA09g048600.4.1C.1, BraA04g005440.4.1C.1	AMP1 description:Peptidase M28 family protein [Source:NCBI gene (formerly Entrezgene);Acc:824637]
AT3G54990.1	BraA09g048930.4.1C.1	SMZ description:Integrase-type DNA-binding superfamily protein [Source:NCBI gene (formerly Entrezgene);Acc:824664]
AT3G54990.2	BraA09g048930.4.1C.1	SMZ description:Integrase-type DNA-binding superfamily protein [Source:NCBI gene (formerly Entrezgene);Acc:824664]
AT3G57230.1	BraA01g004160.4.1C.1, BraA03g060290.4.1C.1	AGL16 description:AGAMOUS-like 16 [Source:NCBI gene (formerly Entrezgene);Acc:824890]
AT3G57230.2	BraA01g004160.4.1C.1, BraA03g060290.4.1C.1	AGL16 description:AGAMOUS-like 16 [Source:NCBI gene (formerly Entrezgene);Acc:824890]

AT3G57230.3	BraA01g004160.4.1C.1, BraA03g060290.4.1C.1	AGL16 description:AGAMOUS-like 16 [Source:NCBI gene (formerly Entrezgene);Acc:824890]
AT3G57230.4	BraA01g004160.4.1C.1, BraA03g060290.4.1C.1	AGL16 description:AGAMOUS-like 16 [Source:NCBI gene (formerly Entrezgene);Acc:824890]
AT3G57230.5	BraA01g004160.4.1C.1, BraA03g060290.4.1C.1	AGL16 description:AGAMOUS-like 16 [Source:NCBI gene (formerly Entrezgene);Acc:824890]
AT3G57300.1	BraA09g051050.4.1C.1, BraA09g051050.4.1C.5, BraA09g051050.4.1C.3, BraA04g003430.4.1C.6, BraA04g003430.4.1C.3, BraA04g003430.4.1C.2, BraA04g003430.4.1C.1, BraA09g051050.4.1C.7	INO80 description:DNA helicase INO80-like protein [Source:NCBI gene (formerly Entrezgene);Acc:824897]
AT3G57300.3	BraA09g051050.4.1C.7, BraA09g051050.4.1C.1, BraA09g051050.4.1C.5, BraA09g051050.4.1C.3, BraA04g003430.4.1C.6, BraA04g003430.4.1C.3, BraA04g003430.4.1C.2, BraA04g003430.4.1C.1	INO80 description:DNA helicase INO80-like protein [Source:NCBI gene (formerly Entrezgene);Acc:824897]
AT3G57300.4	BraA09g051050.4.1C.1, BraA09g051050.4.1C.5, BraA09g051050.4.1C.3, BraA04g003430.4.1C.6, BraA04g003430.4.1C.3, BraA04g003430.4.1C.2, BraA04g003430.4.1C.1, BraA09g051050.4.1C.7	INO80 description:DNA helicase INO80-like protein [Source:NCBI gene (formerly Entrezgene);Acc:824897]
AT3G57390.1	BraA09g051120.4.1C.1, BraA04g003390.4.1C.1	AGL18 description:AGAMOUS-like 18 [Source:NCBI gene (formerly Entrezgene);Acc:824906]
AT3G57390.2	BraA04g003390.4.1C.1	AGL18 description:AGAMOUS-like 18 [Source:NCBI gene (formerly Entrezgene);Acc:824906]

AT3G57390.3	BraA09g051120.4.1C.1, BraA04g003390.4.1C.1	AGL18 description:AGAMOUS-like 18 [Source:NCBI gene (formerly Entrezgene);Acc:824906]
AT3G60250.1	BraA07g025230.4.1C.1	CKB3 description:casein kinase II beta chain 3 [Source:NCBI gene (formerly Entrezgene);Acc:825196]
AT3G60250.2	BraA07g025230.4.1C.1	CKB3 description:casein kinase II beta chain 3 [Source:NCBI gene (formerly Entrezgene);Acc:825196]
AT3G63010.1	BraA07g026460.4.1C.1, BraA04g000390.4.1C.1, BraA09g055310.4.1C.1	GID1B description:alpha/beta-Hydrolases superfamily protein [Source:NCBI gene (formerly Entrezgene);Acc:825476]
AT4G00450.1	BraA09g000350.4.1C.2, BraA09g000350.4.1C.4, BraA09g000350.4.1C.5, BraA09g000350.4.1C.3, BraA09g000350.4.1C.1	CCT description:RNA polymerase II transcription mediator [Source:NCBI gene (formerly Entrezgene);Acc:827954]
AT4G00450.2	BraA09g000350.4.1C.2, BraA09g000350.4.1C.4, BraA09g000350.4.1C.5, BraA09g000350.4.1C.3, BraA09g000350.4.1C.1	CCT description:RNA polymerase II transcription mediator [Source:NCBI gene (formerly Entrezgene);Acc:827954]
AT4G00450.3	BraA09g000350.4.1C.2, BraA09g000350.4.1C.4, BraA09g000350.4.1C.5, BraA09g000350.4.1C.3, BraA09g000350.4.1C.1	CCT description:RNA polymerase II transcription mediator [Source:NCBI gene (formerly Entrezgene);Acc:827954]
AT4G01060.1	BraA03g030470.4.1C.1, BraA02g027660.4.1C.1, BraA09g000800.4.1C.1	CPL3 description:CAPRICE-like MYB3 [Source:NCBI gene (formerly Entrezgene);Acc:827917]
AT4G01060.2	BraA03g030470.4.1C.1, BraA02g027660.4.1C.1, BraA09g000800.4.1C.1	CPL3 description:CAPRICE-like MYB3 [Source:NCBI gene (formerly Entrezgene);Acc:827917]
AT4G01060.3	BraA03g030470.4.1C.1, BraA02g027660.4.1C.1, BraA09g000800.4.1C.1	CPL3 description:CAPRICE-like MYB3 [Source:NCBI gene (formerly Entrezgene);Acc:827917]

AT4G02780.1	BraA09g002340.4.1C.1	GA1 description:Terpenoid cyclases/Protein prenyltransferases superfamily protein [Source:NCBI gene (formerly Entrezgene);Acc:828182]
AT4G03400.1	BraA09g002740.4.1C.1, BraA09g002740.4.1C.11, BraA03g043660.4.1C.1	DFL2 description:Auxin-responsive GH3 family protein [Source:NCBI gene (formerly Entrezgene);Acc:827938]
AT4G03400.2	BraA09g002740.4.1C.1, BraA09g002740.4.1C.11, BraA03g043660.4.1C.1	DFL2 description:Auxin-responsive GH3 family protein [Source:NCBI gene (formerly Entrezgene);Acc:827938]
AT4G04890.1	BraA09g026530.4.1C.3, BraA09g026530.4.1C.2, BraA09g026530.4.1C.1, BraA09g026530.4.1C.4, BraA08g015400.4.1C.1	PDF2 description:protodermal factor 2 [Source:NCBI gene (formerly Entrezgene);Acc:825828]
AT4G04890.2	BraA09g026530.4.1C.3, BraA09g026530.4.1C.2, BraA09g026530.4.1C.1, BraA09g026530.4.1C.4, BraA08g015400.4.1C.1	PDF2 description:protodermal factor 2 [Source:NCBI gene (formerly Entrezgene);Acc:825828]
AT4G08920.1	BraA09g030800.4.1C.1, BraA09g030800.4.1C.4, BraA09g030800.4.1C.5	CRY1 description:cryptochrome 1 [Source:NCBI gene (formerly Entrezgene);Acc:826470]
AT4G11110.1	BraA09g028660.4.1C.2, BraA09g028660.4.1C.1	SPA2 description:SPA1-related 2 [Source:NCBI gene (formerly Entrezgene);Acc:826712]
AT4G11880.2	BraA09g027750.4.1C.1, BraA03g051880.4.1C.1, BraA01g013730.4.1C.1, BraA08g015890.4.1C.1	AGL14 description:AGAMOUS-like 14 [Source:NCBI gene (formerly Entrezgene);Acc:826795]
AT4G11880.3	BraA09g027750.4.1C.1, BraA03g051880.4.1C.1, BraA01g013730.4.1C.1, BraA08g015890.4.1C.1	AGL14 description:AGAMOUS-like 14 [Source:NCBI gene (formerly Entrezgene);Acc:826795]
AT4G14110.1	BraA04g008490.4.1C.1, BraA08g010430.4.1C.1	COP9 description:COP9 signalosome, subunit CSN8 [Source:NCBI gene (formerly Entrezgene);Acc:827049]

AT4G14690.1	BraA01g022250.4.1C.1	ELIP2 description:Chlorophyll A-B binding family protein [Source:NCBI gene (formerly Entrezgene);Acc:827119]
AT4G15090.1	BraA05g016390.4.1C.4, BraA05g016390.4.1C.3, BraA05g016390.4.1C.1	FAR1 description:FRS (FAR1 Related Sequences) transcription factor family [Source:NCBI gene (formerly Entrezgene);Acc:827173]
AT4G15090.2	BraA05g016390.4.1C.4, BraA05g016390.4.1C.3, BraA05g016390.4.1C.1	FAR1 description:FRS (FAR1 Related Sequences) transcription factor family [Source:NCBI gene (formerly Entrezgene);Acc:827173]
AT4G15090.3	BraA05g016390.4.1C.4, BraA05g016390.4.1C.3, BraA05g016390.4.1C.1	FAR1 description:FRS (FAR1 Related Sequences) transcription factor family [Source:NCBI gene (formerly Entrezgene);Acc:827173]
AT4G15090.4	BraA05g016390.4.1C.4, BraA05g016390.4.1C.3, BraA05g016390.4.1C.1	FAR1 description:FRS (FAR1 Related Sequences) transcription factor family [Source:NCBI gene (formerly Entrezgene);Acc:827173]
AT4G15090.5	BraA05g016390.4.1C.4, BraA05g016390.4.1C.3, BraA05g016390.4.1C.1	FAR1 description:FRS (FAR1 Related Sequences) transcription factor family [Source:NCBI gene (formerly Entrezgene);Acc:827173]
AT4G16250.1	BraA05g032610.4.1C.1, BraA05g032610.4.1C.3, BraA05g032610.4.1C.4	PHYD description:phytochrome D [Source:NCBI gene (formerly Entrezgene);Acc:827319]
AT4G16280.1	BraA01g020680.4.1C.1	FCA description:flowering time control protein FCA [Source:NCBI gene (formerly Entrezgene);Acc:827323]
AT4G16280.2	BraA01g020680.4.1C.1	FCA description:flowering time control protein FCA [Source:NCBI gene (formerly Entrezgene);Acc:827323]
AT4G16280.3	BraA01g020680.4.1C.1	FCA description:flowering time control protein FCA [Source:NCBI gene (formerly Entrezgene);Acc:827323]
AT4G16280.4	BraA01g020680.4.1C.1	FCA description:flowering time control protein FCA [Source:NCBI gene (formerly Entrezgene);Acc:827323]

AT4G16780.1	BraA01g019830.4.1C.1	HB-2 description:homeobox protein 2 [Source:NCBI gene (formerly Entrezgene);Acc:827384]
AT4G16845.1	BraA08g012890.4.1C.6, BraA08g012890.4.1C.5, BraA08g012890.4.1C.3, BraA08g012890.4.1C.2, BraA08g012890.4.1C.4, BraA08g012890.4.1C.8	VRN2 description:VEFS-Box of polycomb protein [Source:NCBI gene (formerly Entrezgene);Acc:827392]
AT4G16845.2	BraA08g012890.4.1C.8, BraA08g012890.4.1C.6, BraA08g012890.4.1C.5, BraA08g012890.4.1C.3, BraA08g012890.4.1C.2, BraA08g012890.4.1C.4	VRN2 description:VEFS-Box of polycomb protein [Source:NCBI gene (formerly Entrezgene);Acc:827392]
AT4G17640.1	BraA03g048580.4.1C.2, BraA03g048580.4.1C.3, BraA03g048580.4.1C.4	CKB2 description:casein kinase II beta chain 2 [Source:NCBI gene (formerly Entrezgene);Acc:827484]
AT4G17640.2	BraA03g048580.4.1C.3, BraA03g048580.4.1C.2, BraA03g048580.4.1C.4, BraA02g035040.4.1C.2	CKB2 description:casein kinase II beta chain 2 [Source:NCBI gene (formerly Entrezgene);Acc:827484]
AT4G18130.1	BraA01g009770.4.1C.1	PHYE description:phytochrome E [Source:NCBI gene (formerly Entrezgene);Acc:827538]
AT4G20370.1	BraA07g041570.4.1C.1	TSF description:PEBP (phosphatidylethanolamine-binding protein) family protein [Source:NCBI gene (formerly Entrezgene);Acc:827785]
AT4G21200.1	BraA08g015110.4.1C.1, BraA01g012040.4.1C.1, BraA03g050660.4.1C.1	GA2OX8 description:gibberellin 2-oxidase 8 [Source:NCBI gene (formerly Entrezgene);Acc:827868]
AT4G21200.3	BraA03g050660.4.1C.1, BraA08g015110.4.1C.1, BraA01g012040.4.1C.1	GA2OX8 description:gibberellin 2-oxidase 8 [Source:NCBI gene (formerly Entrezgene);Acc:827868]
AT4G21690.1	BraA08g014930.4.1C.1	GA3OX3 description:gibberellin 3-oxidase 3 [Source:NCBI gene (formerly Entrezgene);Acc:828256]

AT4G22140.1	BraA01g012880.4.1C.1, BraA03g051250.4.1C.1, BraA08g015580.4.1C.3	EBS description:PHD finger family protein / bromo-adjacent homology (BAH) domain-containing protein [Source:NCBI gene (formerly Entrezgene);Acc:828303]
AT4G22140.2	BraA01g012880.4.1C.1, BraA08g015580.4.1C.3, BraA03g051250.4.1C.1	EBS description:PHD finger family protein / bromo-adjacent homology (BAH) domain-containing protein [Source:NCBI gene (formerly Entrezgene);Acc:828303]
AT4G22140.3	BraA01g012880.4.1C.1, BraA03g051250.4.1C.1, BraA08g015580.4.1C.3	EBS description:PHD finger family protein / bromo-adjacent homology (BAH) domain-containing protein [Source:NCBI gene (formerly Entrezgene);Acc:828303]
AT4G22950.1	BraA08g015890.4.1C.1	AGL19 description:AGAMOUS-like 19 [Source:NCBI gene (formerly Entrezgene);Acc:828394]
AT4G22950.2	BraA03g051880.4.1C.1, BraA01g013730.4.1C.1, BraA08g015890.4.1C.1	AGL19 description:AGAMOUS-like 19 [Source:NCBI gene (formerly Entrezgene);Acc:828394]
AT4G22950.3	BraA08g015890.4.1C.1	AGL19 description:AGAMOUS-like 19 [Source:NCBI gene (formerly Entrezgene);Acc:828394]
AT4G23340.1	BraA03g052270.4.1C.1	AT4G23340 description:2-oxoglutarate (2OG) and Fe(II)-dependent oxygenase superfamily protein [Source:NCBI gene (formerly Entrezgene);Acc:828433]
AT4G23340.2	BraA03g052270.4.1C.1	AT4G23340 description:2-oxoglutarate (2OG) and Fe(II)-dependent oxygenase superfamily protein [Source:NCBI gene (formerly Entrezgene);Acc:828433]
AT4G24540.1	BraA01g015410.4.1C.1	AGL24 description:AGAMOUS-like 24 [Source:NCBI gene (formerly Entrezgene);Acc:828556]

AT4G24620.1	BraA01g015490.4.1C.1, BraA03g053260.4.1C.1, BraA03g053260.4.1C.2	PGI1 description:phosphoglucose isomerase 1 [Source:NCBI gene (formerly Entrezgene);Acc:828564]
AT4G24620.2	BraA01g015490.4.1C.1, BraA03g053260.4.1C.1, BraA03g053260.4.1C.2	PGI1 description:phosphoglucose isomerase 1 [Source:NCBI gene (formerly Entrezgene);Acc:828564]
AT4G25420.1	BraA03g053810.4.1C.1, BraA01g016260.4.1C.1	GA20OX1 description:2-oxoglutarate (2OG) and Fe(II)-dependent oxygenase superfamily protein [Source:NCBI gene (formerly Entrezgene);Acc:828645]
AT4G25420.2	BraA03g053810.4.1C.1, BraA01g016260.4.1C.1	GA20OX1 description:2-oxoglutarate (2OG) and Fe(II)-dependent oxygenase superfamily protein [Source:NCBI gene (formerly Entrezgene);Acc:828645]
AT4G27430.1	BraA01g018260.4.1C.1, BraA01g018260.4.1C.3, BraA01g018260.4.1C.2	CIP7 description:COP1-interacting protein 7 [Source:NCBI gene (formerly Entrezgene);Acc:828851]
AT4G27430.2	BraA01g018260.4.1C.1, BraA01g018260.4.1C.3, BraA01g018260.4.1C.2	CIP7 description:COP1-interacting protein 7 [Source:NCBI gene (formerly Entrezgene);Acc:828851]
AT4G29130.1	BraA09g012660.4.1C.1, BraA07g000880.4.1C.2, BraA07g000880.4.1C.1, BraA07g000880.4.1C.3	HXK1 description:hexokinase 1 [Source:NCBI gene (formerly Entrezgene);Acc:829034]
AT4G29830.1	BraA01g007960.4.1C.1	VIP3 description:Transducin/WD40 repeat-like superfamily protein [Source:NCBI gene (formerly Entrezgene);Acc:829105]
AT4G31120.1	BraA03g057890.4.1C.1, BraA08g018520.4.1C.1, BraA08g018520.4.1C.2	SKB1 description:SHK1 binding protein 1 [Source:NCBI gene (formerly Entrezgene);Acc:829240]
AT4G31120.2	BraA03g057890.4.1C.1, BraA08g018520.4.1C.1, BraA08g018520.4.1C.2	SKB1 description:SHK1 binding protein 1 [Source:NCBI gene (formerly Entrezgene);Acc:829240]
AT4G31380.1	BraA01g006620.4.1C.1, BraA08g018340.4.1C.1, BraA03g058020.4.1C.1	FLP1 description:flowering-promoting factor-like protein [Source:NCBI gene (formerly Entrezgene);Acc:28720195]

AT4G31500.1	BraA08g007880.4.1C.1	CYP83B1 description:cytochrome P450, family 83, subfamily B, polypeptide 1 [Source:NCBI gene (formerly Entrezgene);Acc:829277]
AT4G32040.1	BraA01g006060.4.1C.1	KNAT5 description:homeobox protein knotted-1-like 5 [Source:NCBI gene (formerly Entrezgene);Acc:829335]
AT4G32040.2	BraA01g006060.4.1C.1	KNAT5 description:homeobox protein knotted-1-like 5 [Source:NCBI gene (formerly Entrezgene);Acc:829335]
AT4G35900.1	BraA01g002750.4.1C.1, BraA03g061470.4.1C.1	FD description:Basic-leucine zipper (bZIP) transcription factor family protein [Source:NCBI gene (formerly Entrezgene);Acc:829744]
AT4G35900.2	BraA01g002750.4.1C.1, BraA03g061470.4.1C.1	FD description:Basic-leucine zipper (bZIP) transcription factor family protein [Source:NCBI gene (formerly Entrezgene);Acc:829744]
AT4G36920.1	BraA01g001770.4.1C.2, BraA03g062330.4.1C.1, BraA03g062330.4.1C.3, BraA01g001770.4.1C.1, BraA03g062330.4.1C.2	AP2 description:Integrase-type DNA-binding superfamily protein [Source:NCBI gene (formerly Entrezgene);Acc:829845]
AT4G36920.2	BraA01g001770.4.1C.2, BraA03g062330.4.1C.1, BraA03g062330.4.1C.3, BraA01g001770.4.1C.1, BraA03g062330.4.1C.2	AP2 description:Integrase-type DNA-binding superfamily protein [Source:NCBI gene (formerly Entrezgene);Acc:829845]
AT4G37580.1	BraA08g022260.4.1C.1, BraA03g062590.4.1C.1	HLS1 description:Acyl-CoA N-acyltransferases (NAT) superfamily protein [Source:NCBI gene (formerly Entrezgene);Acc:829913]
AT4G37580.2	BraA03g062590.4.1C.1, BraA08g022260.4.1C.1, BraA04g018840.4.1C.1	HLS1 description:Acyl-CoA N-acyltransferases (NAT) superfamily protein [Source:NCBI gene (formerly Entrezgene);Acc:829913]
AT4G37940.1	BraA01g001170.4.1C.1	AGL21 description:AGAMOUS-like 21 [Source:NCBI gene (formerly Entrezgene);Acc:829950]

AT4G37940.2	BraA01g001170.4.1C.1	AGL21 description:AGAMOUS-like 21 [Source:NCBI gene (formerly Entrezgene);Acc:829950]
AT4G37940.3	BraA01g001170.4.1C.1	AGL21 description:AGAMOUS-like 21 [Source:NCBI gene (formerly Entrezgene);Acc:829950]
AT4G39400.1	BraA06g047220.4.1C.1, BraA01g000530.4.1C.1, BraA01g000530.4.1C.2, BraA08g023180.4.1C.2, BraA08g023180.4.1C.1	BRI1 description:Leucine-rich receptor-like protein kinase family protein [Source:NCBI gene (formerly Entrezgene);Acc:830095]
AT5G02840.1	BraA10g033230.4.1C.2, BraA10g033230.4.1C.1, BraA03g001160.4.1C.1, BraA03g001160.4.1C.2	LCL1 description:LHY/CCA1-like 1 [Source:NCBI gene (formerly Entrezgene);Acc:831766]
AT5G02840.2	BraA10g033230.4.1C.2, BraA10g033230.4.1C.1, BraA03g001160.4.1C.1, BraA03g001160.4.1C.2	LCL1 description:LHY/CCA1-like 1 [Source:NCBI gene (formerly Entrezgene);Acc:831766]
AT5G02840.3	BraA10g033230.4.1C.1, BraA10g033230.4.1C.2, BraA03g001160.4.1C.2, BraA03g001160.4.1C.1	LCL1 description:LHY/CCA1-like 1 [Source:NCBI gene (formerly Entrezgene);Acc:831766]
AT5G02840.4	BraA10g033230.4.1C.2, BraA10g033230.4.1C.1, BraA03g001160.4.1C.2, BraA03g001160.4.1C.1	LCL1 description:LHY/CCA1-like 1 [Source:NCBI gene (formerly Entrezgene);Acc:831766]
AT5G03790.1	BraA10g032700.4.1C.1	HB51 description:homeobox 51 [Source:NCBI gene (formerly Entrezgene);Acc:831723]
AT5G03790.2	BraA10g032700.4.1C.1, BraA03g001470.4.1C.1	HB51 description:homeobox 51 [Source:NCBI gene (formerly Entrezgene);Acc:831723]
AT5G03840.1	BraA03g001510.4.1C.1, BraA10g032670.4.1C.1	TFL1 description:PEBP (phosphatidylethanolamine-binding protein) family protein [Source:NCBI gene (formerly Entrezgene);Acc:831683]
AT5G05690.1	BraA10g028790.4.1C.1, BraA02g001850.4.1C.1	CPD description:Cytochrome P450 superfamily protein [Source:NCBI gene (formerly Entrezgene);Acc:830453]

AT5G05690.2	BraA10g028790.4.1C.1, BraA02g001850.4.1C.1	CPD description:Cytochrome P450 superfamily protein [Source:NCBI gene (formerly Entrezgene);Acc:830453]
AT5G05690.3	BraA10g028790.4.1C.2, BraA10g028790.4.1C.1, BraA02g001850.4.1C.1	CPD description:Cytochrome P450 superfamily protein [Source:NCBI gene (formerly Entrezgene);Acc:830453]
AT5G07200.1	BraA02g002320.4.1C.1, BraA10g030280.4.1C.1, BraA03g003020.4.1C.1	GA20OX3 description:gibberellin 20-oxidase 3 [Source:NCBI gene (formerly Entrezgene);Acc:830611]
AT5G10140.1	BraA02g003400.4.1C.1, BraA10g028070.4.1C.1, BraA03g004340.4.1C.1, BraA03g016210.4.1C.1	FLC description:K-box region and MADS-box transcription factor family protein [Source:NCBI gene (formerly Entrezgene);Acc:830878]
AT5G10140.2	BraA10g028070.4.1C.1, BraA02g003400.4.1C.1, BraA03g004340.4.1C.1, BraA03g016210.4.1C.1	FLC description:K-box region and MADS-box transcription factor family protein [Source:NCBI gene (formerly Entrezgene);Acc:830878]
AT5G10140.3	BraA10g028070.4.1C.1, BraA02g003400.4.1C.1, BraA03g004340.4.1C.1, BraA03g016210.4.1C.1	FLC description:K-box region and MADS-box transcription factor family protein [Source:NCBI gene (formerly Entrezgene);Acc:830878]
AT5G10140.4	BraA10g028070.4.1C.1, BraA02g003400.4.1C.1, BraA03g004340.4.1C.1, BraA03g016210.4.1C.1	FLC description:K-box region and MADS-box transcription factor family protein [Source:NCBI gene (formerly Entrezgene);Acc:830878]
AT5G10625.1	BraA10g027630.4.1C.1	AT5G10625 description:flowering-promoting factor-like protein [Source:NCBI gene (formerly Entrezgene);Acc:2745983]
AT5G11260.1	BraA10g027210.4.1C.1, BraA02g003960.4.1C.1	HY5 description:Basic-leucine zipper (bZIP) transcription factor family protein [Source:NCBI gene (formerly Entrezgene);Acc:830996]
AT5G11260.2	BraA10g027210.4.1C.1	HY5 description:Basic-leucine zipper (bZIP) transcription factor family protein [Source:NCBI gene (formerly Entrezgene);Acc:830996]
AT5G12840.1	BraA10g026180.4.1C.1	NF-YA1 description:nuclear factor Y, subunit A1 [Source:NCBI gene (formerly Entrezgene);Acc:831124]

AT5G12840.2	BraA10g026180.4.1C.1	NF-YA1 description:nuclear factor Y, subunit A1 [Source:NCBI gene (formerly Entrezgene);Acc:831124]
AT5G12840.3	BraA10g026180.4.1C.1	NF-YA1 description:nuclear factor Y, subunit A1 [Source:NCBI gene (formerly Entrezgene);Acc:831124]
AT5G12840.4	BraA10g026180.4.1C.1	NF-YA1 description:nuclear factor Y, subunit A1 [Source:NCBI gene (formerly Entrezgene);Acc:831124]
AT5G12840.5	BraA10g026180.4.1C.1	NF-YA1 description:nuclear factor Y, subunit A1 [Source:NCBI gene (formerly Entrezgene);Acc:831124]
AT5G13480.1	BraA03g005920.4.1C.2, BraA03g005920.4.1C.1, BraA02g005030.4.1C.1	FY description:Transducin/WD40 repeat-like superfamily protein [Source:NCBI gene (formerly Entrezgene);Acc:831191]
AT5G13480.2	BraA03g005920.4.1C.2, BraA03g005920.4.1C.1, BraA02g005030.4.1C.1	FY description:Transducin/WD40 repeat-like superfamily protein [Source:NCBI gene (formerly Entrezgene);Acc:831191]
AT5G13480.3	BraA03g005920.4.1C.2, BraA03g005920.4.1C.1, BraA02g005030.4.1C.1	FY description:Transducin/WD40 repeat-like superfamily protein [Source:NCBI gene (formerly Entrezgene);Acc:831191]
AT5G13480.4	BraA03g005920.4.1C.2, BraA03g005920.4.1C.1, BraA02g005030.4.1C.1	FY description:Transducin/WD40 repeat-like superfamily protein [Source:NCBI gene (formerly Entrezgene);Acc:831191]
AT5G15230.1	BraA02g006020.4.1C.1	GASA4 description:GAST1 protein homolog 4 [Source:NCBI gene (formerly Entrezgene);Acc:831375]
AT5G15230.2	BraA02g006020.4.1C.1	GASA4 description:GAST1 protein homolog 4 [Source:NCBI gene (formerly Entrezgene);Acc:831375]
AT5G19550.1	BraA02g008430.4.1C.1, BraA10g021140.4.1C.1, BraA03g065310.4.1C.1, BraA03g065300.4.1C.1	ASP2 description:aspartate aminotransferase 2 [Source:NCBI gene (formerly Entrezgene);Acc:832075]

AT5G24860.1	BraA09g006280.4.1C.1, BraA06g034940.4.1C.1	FPF1 description:flowering promoting factor 1 [Source:NCBI gene (formerly Entrezgene);Acc:832555]
AT5G25810.1	BraA06g035680.4.1C.1	tny description:Integrase-type DNA-binding superfamily protein [Source:NCBI gene (formerly Entrezgene);Acc:832650]
AT5G25900.1	BraA06g035740.4.1C.1	GA3 description:GA requiring 3 [Source:NCBI gene (formerly Entrezgene);Acc:832659]
AT5G27320.1	BraA06g036840.4.1C.1, BraA06g036840.4.1C.2	GID1C description:alpha/beta-Hydrolases superfamily protein [Source:NCBI gene (formerly Entrezgene);Acc:832790]
AT5G28490.1	BraA02g042470.4.1C.1, BraA06g037110.4.1C.2, BraA06g037110.4.1C.1, BraA09g004870.4.1C.1, BraA01g044930.4.1C.1	LSH1 description:LIGHT-DEPENDENT SHORT HYPOCOTYLS-like protein (DUF640) [Source:NCBI gene (formerly Entrezgene);Acc:832941]
AT5G35840.1	BraA05g015780.4.1C.1	PHYC description:phytochrome C [Source:NCBI gene (formerly Entrezgene);Acc:833570]
AT5G37770.1	BraA04g016490.4.1C.1	TCH2 description:EF hand calcium-binding protein family [Source:NCBI gene (formerly Entrezgene);Acc:833755]
AT5G37770.2	BraA04g016490.4.1C.1	TCH2 description:EF hand calcium-binding protein family [Source:NCBI gene (formerly Entrezgene);Acc:833755]
AT5G39660.1	BraA04g013590.4.1C.1	CDF2 description:cycling DOF factor 2 [Source:NCBI gene (formerly Entrezgene);Acc:833962]
AT5G39660.2	BraA04g013590.4.1C.1	CDF2 description:cycling DOF factor 2 [Source:NCBI gene (formerly Entrezgene);Acc:833962]
AT5G39660.3	BraA04g013590.4.1C.1	CDF2 description:cycling DOF factor 2 [Source:NCBI gene (formerly Entrezgene);Acc:833962]

AT5G41360.1	BraA04g015630.4.1C.1, BraA07g021530.4.1C.2, BraA04g015630.4.1C.3	XPB2 description:homolog of Xeroderma pigmentosum complementation group B 2 [Source:NCBI gene (formerly Entrezgene);Acc:834138]
AT5G41360.2	BraA04g015630.4.1C.1, BraA07g021530.4.1C.2, BraA04g015630.4.1C.3	XPB2 description:homolog of Xeroderma pigmentosum complementation group B 2 [Source:NCBI gene (formerly Entrezgene);Acc:834138]
AT5G46210.1	BraA06g044900.4.1C.1, BraA02g034080.4.1C.1, BraA09g022630.4.1C.1, BraA02g034080.4.1C.2	CUL4 description:cullin4 [Source:NCBI gene (formerly Entrezgene);Acc:834663]
AT5G46910.1	BraA02g034720.4.1C.3, BraA02g034720.4.1C.1, BraA09g023370.4.1C.5, BraA09g023370.4.1C.1	AT5G46910 description:Transcription factor jumonji (jmj) family protein / zinc finger (C5HC2 type) family protein [Source:NCBI gene (formerly Entrezgene);Acc:834736]
AT5G46910.2	BraA02g034720.4.1C.3, BraA02g034720.4.1C.1, BraA09g023370.4.1C.5, BraA09g023370.4.1C.1	AT5G46910 description:Transcription factor jumonji (jmj) family protein / zinc finger (C5HC2 type) family protein [Source:NCBI gene (formerly Entrezgene);Acc:834736]
AT5G47010.1	BraA02g034910.4.1C.3, BraA02g034910.4.1C.2, BraA02g034910.4.1C.1	LBA1 description:RNA helicase [Source:NCBI gene (formerly Entrezgene);Acc:834747]
AT5G47080.1	BraA02g035040.4.1C.2, BraA02g035040.4.1C.1	CKB1 description:casein kinase II beta chain 1 [Source:NCBI gene (formerly Entrezgene);Acc:834754]
AT5G47080.2	BraA02g035040.4.1C.2, BraA02g035040.4.1C.1	CKB1 description:casein kinase II beta chain 1 [Source:NCBI gene (formerly Entrezgene);Acc:834754]
AT5G47080.3	BraA02g035040.4.1C.2, BraA02g035040.4.1C.1	CKB1 description:casein kinase II beta chain 1 [Source:NCBI gene (formerly Entrezgene);Acc:834754]
AT5G47080.4	BraA02g035040.4.1C.1, BraA02g035040.4.1C.2, BraA03g048580.4.1C.2	CKB1 description:casein kinase II beta chain 1 [Source:NCBI gene (formerly Entrezgene);Acc:834754]

AT5G48150.1	BraA02g041330.4.1C.4, BraA02g041330.4.1C.3, BraA02g041330.4.1C.2, BraA02g041330.4.1C.1, BraA02g041330.4.1C.6, BraA09g004360.4.1C.6, BraA09g004360.4.1C.3, BraA09g004360.4.1C.2, BraA09g004360.4.1C.1, BraA09g004360.4.1C.5, BraA06g038550.4.1C.1	PAT1 description:GRAS family transcription factor [Source:NCBI gene (formerly Entrezgene);Acc:834867]
AT5G48150.2	BraA02g041330.4.1C.4, BraA02g041330.4.1C.3, BraA02g041330.4.1C.2, BraA02g041330.4.1C.1, BraA02g041330.4.1C.6, BraA09g004360.4.1C.6, BraA09g004360.4.1C.3, BraA09g004360.4.1C.2, BraA09g004360.4.1C.1, BraA09g004360.4.1C.5, BraA06g038550.4.1C.1	PAT1 description:GRAS family transcription factor [Source:NCBI gene (formerly Entrezgene);Acc:834867]
AT5G48150.3	BraA02g041330.4.1C.4, BraA02g041330.4.1C.3, BraA02g041330.4.1C.2, BraA02g041330.4.1C.1, BraA02g041330.4.1C.6, BraA09g004360.4.1C.6, BraA09g004360.4.1C.3, BraA09g004360.4.1C.2, BraA09g004360.4.1C.1, BraA09g004360.4.1C.5, BraA06g038550.4.1C.1	PAT1 description:GRAS family transcription factor [Source:NCBI gene (formerly Entrezgene);Acc:834867]
AT5G48150.4	BraA02g041330.4.1C.4, BraA02g041330.4.1C.3, BraA02g041330.4.1C.2, BraA02g041330.4.1C.1, BraA02g041330.4.1C.6, BraA09g004360.4.1C.6, BraA09g004360.4.1C.3, BraA09g004360.4.1C.2,	PAT1 description:GRAS family transcription factor [Source:NCBI gene (formerly Entrezgene);Acc:834867]

	BraA09g004360.4.1C.1, BraA09g004360.4.1C.5, BraA06g038550.4.1C.1	
AT5G49020.1	BraA02g041890.4.1C.1, BraA05g041010.4.1C.1	PRMT4A description:protein arginine methyltransferase 4A [Source:NCBI gene (formerly Entrezgene);Acc:834961]
AT5G49020.2	BraA02g041890.4.1C.1, BraA05g041010.4.1C.1	PRMT4A description:protein arginine methyltransferase 4A [Source:NCBI gene (formerly Entrezgene);Acc:834961]
AT5G51230.1	BraA02g016460.4.1C.2, BraA02g016460.4.1C.3, BraA02g016460.4.1C.4	EMF2 description:VEFS-Box of polycomb protein [Source:NCBI gene (formerly Entrezgene);Acc:835198]
AT5G51230.2	BraA02g016460.4.1C.3, BraA02g016460.4.1C.2, BraA02g016460.4.1C.4	EMF2 description:VEFS-Box of polycomb protein [Source:NCBI gene (formerly Entrezgene);Acc:835198]
AT5G51230.3	BraA02g016460.4.1C.3, BraA02g016460.4.1C.2, BraA02g016460.4.1C.4	EMF2 description:VEFS-Box of polycomb protein [Source:NCBI gene (formerly Entrezgene);Acc:835198]
AT5G51310.1	BraA02g016430.4.1C.1	AT5G51310 description:2-oxoglutarate (2OG) and Fe(II)-dependent oxygenase superfamily protein [Source:NCBI gene (formerly Entrezgene);Acc:835205]
AT5G51810.1	BraA10g010880.4.1C.1, BraA02g016150.4.1C.1	GA20OX2 description:gibberellin 20 oxidase 2 [Source:NCBI gene (formerly Entrezgene);Acc:835256]
AT5G51810.2	BraA10g010880.4.1C.1, BraA02g016150.4.1C.1	GA20OX2 description:gibberellin 20 oxidase 2 [Source:NCBI gene (formerly Entrezgene);Acc:835256]
AT5G51820.1	BraA10g010900.4.1C.1, BraA10g010900.4.1C.5, BraA10g010900.4.1C.2, BraA10g010900.4.1C.4	PGM description:phosphoglucomutase [Source:NCBI gene (formerly Entrezgene);Acc:835257]
AT5G54510.1	BraA02g014240.4.1C.1, BraA03g014210.4.1C.1, BraA03g054930.4.1C.1, BraA01g018090.4.1C.1	DFL1 description:Auxin-responsive GH3 family protein [Source:NCBI gene (formerly Entrezgene);Acc:835539]

AT5G58230.1	BraA03g012300.4.1C.1, BraA10g016640.4.1C.1, BraA02g012010.4.1C.1	MSI1 description:Transducin/WD40 repeat-like superfamily protein [Source:NCBI gene (formerly Entrezgene);Acc:835935]
AT5G59560.1	BraA02g011180.4.1C.2, BraA02g011180.4.1C.1	SRR1 description:sensitivity to red light reduced protein (SRR1) [Source:NCBI gene (formerly Entrezgene);Acc:836075]
AT5G59560.2	BraA02g011180.4.1C.2, BraA02g011180.4.1C.1	SRR1 description:sensitivity to red light reduced protein (SRR1) [Source:NCBI gene (formerly Entrezgene);Acc:836075]
AT5G59710.1	BraA02g011020.4.1C.4, BraA02g011020.4.1C.3, BraA02g011020.4.1C.2, BraA03g011440.4.1C.4, BraA03g011440.4.1C.1, BraA02g011020.4.1C.1	VIP2 description:VIRE2 interacting protein 2 [Source:NCBI gene (formerly Entrezgene);Acc:836092]
AT5G59710.2	BraA02g011020.4.1C.4, BraA02g011020.4.1C.3, BraA02g011020.4.1C.2, BraA03g011440.4.1C.4, BraA03g011440.4.1C.1, BraA02g011020.4.1C.1	VIP2 description:VIRE2 interacting protein 2 [Source:NCBI gene (formerly Entrezgene);Acc:836092]
AT5G59710.3	BraA02g011020.4.1C.4, BraA02g011020.4.1C.3, BraA02g011020.4.1C.2, BraA03g011440.4.1C.4, BraA03g011440.4.1C.1, BraA02g011020.4.1C.1, BraA02g011020.4.1C.6	VIP2 description:VIRE2 interacting protein 2 [Source:NCBI gene (formerly Entrezgene);Acc:836092]
AT5G60910.1	BraA03g044550.4.1C.1, BraA02g044850.4.1C.1	AGL8 description:AGAMOUS-like 8 [Source:NCBI gene (formerly Entrezgene);Acc:836212]
AT5G60910.2	BraA03g044550.4.1C.1, BraA02g044850.4.1C.1	AGL8 description:AGAMOUS-like 8 [Source:NCBI gene (formerly Entrezgene);Acc:836212]
AT5G61150.1	BraA09g007090.4.1C.2, BraA09g007090.4.1C.3, BraA02g045030.4.1C.1	VIP4 description:leo1-like family protein [Source:NCBI gene (formerly Entrezgene);Acc:836236]

AT5G61150.2	BraA09g007090.4.1C.2, BraA09g007090.4.1C.3, BraA02g045030.4.1C.1	VIP4 description:leo1-like family protein [Source:NCBI gene (formerly Entrezgene);Acc:836236]
AT5G61850.1	BraA06g027820.4.1C.1, BraA02g045330.4.1C.1	LFY description:floral meristem identity control protein LEAFY (LFY) [Source:NCBI gene (formerly Entrezgene);Acc:836307]
AT5G61850.2	BraA06g027820.4.1C.1, BraA02g045330.4.1C.1	LFY description:floral meristem identity control protein LEAFY (LFY) [Source:NCBI gene (formerly Entrezgene);Acc:836307]
AT5G62040.1	BraA06g027970.4.1C.1	BFT description:PEBP (phosphatidylethanolamine-binding protein) family protein [Source:NCBI gene (formerly Entrezgene);Acc:836324]
AT5G62430.1	BraA02g045850.4.1C.1	CDF1 description:cycling DOF factor 1 [Source:NCBI gene (formerly Entrezgene);Acc:836364]
AT5G62640.1	BraA06g028790.4.1C.1, BraA06g028790.4.1C.5, BraA06g028790.4.1C.6, BraA06g028790.4.1C.2, BraA06g028790.4.1C.3	ELF5 description:proline-rich family protein [Source:NCBI gene (formerly Entrezgene);Acc:836385]
AT5G62640.2	BraA06g028790.4.1C.1, BraA06g028790.4.1C.5, BraA06g028790.4.1C.6, BraA06g028790.4.1C.2, BraA06g028790.4.1C.3	ELF5 description:proline-rich family protein [Source:NCBI gene (formerly Entrezgene);Acc:836385]
AT5G62640.3	BraA06g028790.4.1C.1, BraA06g028790.4.1C.6, BraA06g028790.4.1C.2, BraA06g028790.4.1C.5	ELF5 description:proline-rich family protein [Source:NCBI gene (formerly Entrezgene);Acc:836385]
AT5G64813.1	BraA06g030870.4.1C.1, BraA09g008760.4.1C.1, BraA09g008760.4.1C.3	LIP1 description:Ras-related small GTP-binding family protein [Source:NCBI gene (formerly Entrezgene);Acc:836603]
AT5G64813.2	BraA06g030870.4.1C.1, BraA09g008760.4.1C.1, BraA09g008760.4.1C.3	LIP1 description:Ras-related small GTP-binding family protein [Source:NCBI gene (formerly Entrezgene);Acc:836603]

AT5G64813.3	BraA06g030870.4.1C.1, BraA09g008760.4.1C.1, BraA09g008760.4.1C.3	LIP1 description:Ras-related small GTP-binding family protein [Source:NCBI gene (formerly Entrezgene);Acc:836603]
AT5G65060.3	BraA02g047130.4.1C.1	MAF3 description:K-box region and MADS-box transcription factor family protein [Source:NCBI gene (formerly Entrezgene);Acc:836630]
AT5G65070.1	BraA06g031110.4.1C.1	MAF4 description:K-box region and MADS-box transcription factor family protein [Source:NCBI gene (formerly Entrezgene);Acc:836631]
AT5G65070.6	BraA06g031110.4.1C.1, BraA02g047120.4.1C.1	MAF4 description:K-box region and MADS-box transcription factor family protein [Source:NCBI gene (formerly Entrezgene);Acc:836631]
AT5G65540.1	BraA06g031600.4.1C.1, BraA06g031600.4.1C.2	AT5G65540 description:transcription initiation factor TFIID subunit [Source:NCBI gene (formerly Entrezgene);Acc:836680]
AT5G67100.1	BraA07g017580.4.1C.1	ICU2 description:DNA-directed DNA polymerase [Source:NCBI gene (formerly Entrezgene);Acc:836845]
AT5G67100.2	BraA07g017580.4.1C.1	ICU2 description:DNA-directed DNA polymerase [Source:NCBI gene (formerly Entrezgene);Acc:836845]

a Gene IDs summarized by Brachi et al. (2010) as Arabidopsis thaliana flowering-related genes.

b B. rapa gene IDs identified as homologs based on BLAST analysis with alignment length > 80%, identity > 80%, and E-value < 0.01.

Supplemental Table 4. Differentially expressed genes between GA_3-treated (GA^+) and untreated (GA^-) samples in 'CHOY' at five days after GA_3 application.

Gene ID	Splicing variant for each gene	TPM values for each of the three biological replicates.						Annotation
		GA -			GA+			
		1	2	3	1	2	3	
BraA02g041330.4.1C	BraA02g041330.4.1C.1	10.715	4.621	8.414	12.882	15.631	16.583	PAT1 description:GRAS family transcription factor
	BraA02g041330.4.1C.2	10.715	4.621	8.414	12.882	15.631	16.583	PAT1 description:GRAS family transcription factor
	BraA02g041330.4.1C.3	10.715	4.621	8.414	12.882	15.631	16.583	PAT1 description:GRAS family transcription factor
	BraA02g041330.4.1C.4	10.715	4.621	8.414	12.882	15.631	16.583	PAT1 description:GRAS family transcription factor
	BraA02g041330.4.1C.6	11.402	4.917	8.954	13.708	16.633	17.646	PAT1 description:GRAS family transcription factor
BraA03g006650.4.1C	BraA03g006650.4.1C.1	58.446	43.352	52.853	62.085	67.792	77.767	AT5G14920 description:Gibberellin-regulated family protein
BraA03g030470.4.1	BraA03g030470.4.1C.1	23.174	42.261	47.272	19.678	7.826	16.501	CPL3 description:CAPRICE-like MYB3
BraA03g039240.4.1C	BraA03g039240.4.1C.2	7.678	5.22	10.754	12.954	13.53	13.053	VRN1 description:AP2/B3-like transcriptional factor family protein
	BraA03g039240.4.1C.4	7.678	5.22	10.754	12.954	13.53	13.053	VRN1 description:AP2/B3-like transcriptional factor family protein
BraA03g057890.4.1C	BraA03g057890.4.1C.2	14.25	12.856	12.397	6.533	5.172	9.799	SKB1 description:SHK1 binding protein 1
BraA04g018360.4.1C	BraA04g018360.4.1C.1	12.755	14.079	15.897	16.225	19.494	18.21	SVP description:K-box region and MADS-box transcription factor family protein

	BraA04g018360.4.1C.2	13.464	14.861	16.78	17.126	20.577	19.222	SVP description:K-box region and MADS-box transcription factor family protein
BraA04g030340.4.1C	BraA04g030340.4.1C.1	8.653	4.716	10.905	3.364	2.183	1.578	ELF4 description:EARLY FLOWERING-like protein (DUF1313)
BraA05g007990.4.1C	BraA05g007990.4.1C.1	17.469	15.013	18.352	10.746	12.175	13.2	FHY1 description:far-red elongated hypocotyl 1
	BraA05g007990.4.1C.2	17.359	15.434	18.532	11.757	13.885	12.812	FHY1 description:far-red elongated hypocotyl 1
	BraA05g007990.4.1C.3	17.359	15.434	18.532	11.757	13.885	12.812	FHY1 description:far-red elongated hypocotyl 1
	BraA05g007990.4.1C.4	17.359	15.434	18.532	11.757	13.885	12.812	FHY1 description:far-red elongated hypocotyl 1
BraA07g025700.4.1C	BraA07g025700.4.1C.1	218.18	193.008	202.57	180.135	178.81	170.21	AT5G28450 description:Chlorophyll A-B binding family protein
	BraA07g025700.4.1C.2	218.18	193.008	202.57	180.135	178.81	170.21	AT5G28450 description:Chlorophyll A-B binding family protein
	BraA07g025700.4.1C.4	305.508	268.569	285.827	254.686	252.232	240.502	AT5G28450 description:Chlorophyll A-B binding family protein
	BraA07g025700.4.1C.5	203.784	179.111	186.233	169.749	170.565	156.902	AT5G28450 description:Chlorophyll A-B binding family protein
	BraA07g025700.4.1C.7	346.111	302.686	324.582	289.737	287.264	274.867	AT5G28450 description:Chlorophyll A-B binding family protein
BraA09g002740.4.1C	BraA09g002740.4.1C.2	41.617	43.662	33.581	51.856	53.856	49.432	DFL2 description:Auxin-responsive GH3 family protein
	BraA09g002740.4.1C.3	31.215	32.583	24.138	38.259	39.331	34.99	DFL2 description:Auxin-responsive GH3 family protein

BraA09g002740.4.1C.5	41.617	43.662	33.581	51.856	53.856	49.432	DFL2 description:Auxin-responsive GH3 family protein
BraA09g002740.4.1C.7	22.671	24.078	18.146	28.616	29.343	26.104	DFL2 description:Auxin-responsive GH3 family protein
BraA09g002740.4.1C.8	31.215	32.583	24.138	38.259	39.331	34.99	DFL2 description:Auxin-responsive GH3 family protein
BraA09g002740.4.1C.9	31.215	32.583	24.138	38.259	39.331	34.99	DFL2 description:Auxin-responsive GH3 family protein

Supplemental Table 5. Differentially expressed genes between GA_3-treated (GA^+) and untreated (GA^-) samples in 'Kohiki' at five days after GA_3 application.

Gene ID	Splicing variant for each gene	TPM values for each of the three biological replicates.						Annotation
		GA -			GA+			
		1	2	3	1	2	3	
BraA01g018260.4.1C	BraA01g018260.4.1C.1	22.161	11.026	13.015	30.178	27.798	26.528	CIP7 description:COP1-interacting protein 7
	BraA01g018260.4.1C.2	26.391	12.771	14.991	35.082	32.862	31.57	CIP7 description:COP1-interacting protein 7
	BraA01g018260.4.1C.3	26.391	12.771	14.991	35.082	32.862	31.57	CIP7 description:COP1-interacting protein 7
BraA02g041330.4.1C	BraA02g041330.4.1C.1	8.319	7.887	8.639	10.571	13.593	10.783	PAT1 description:GRAS family transcription factor
	BraA02g041330.4.1C.2	8.319	7.887	8.639	10.571	13.593	10.783	PAT1 description:GRAS family transcription factor
	BraA02g041330.4.1C.3	8.319	7.887	8.639	10.571	13.593	10.783	PAT1 description:GRAS family transcription factor
	BraA02g041330.4.1C.4	8.319	7.887	8.639	10.571	13.593	10.783	PAT1 description:GRAS family transcription factor
	BraA02g041330.4.1C.6	8.853	8.393	9.192	11.248	14.464	11.475	PAT1 description:GRAS family transcription factor
BraA03g018150.4.1C	BraA03g018150.4.1C.1	2.129	3.18	1.257	38.147	29.636	16.742	SPL3 description:squamosa promoter binding protein-like 3
BraA04g001160.4.1C	BraA04g001160.4.1C.2	408.2	255.193	209.065	511.841	448.267	645.328	AT5G28450 description:Chlorophyll A-B binding family protein

	BraA04g001160.4.1C.3	476.337	296.192	238.338	596.658	523.914	755.112	AT5G28450 description:Chlorophyll A-B binding family protein
	BraA04g001160.4.1C.5	476.337	296.192	238.338	596.658	523.914	755.112	AT5G28450 description:Chlorophyll A-B binding family protein
	BraA04g001160.4.1C.4	408.2	255.193	209.065	511.841	448.267	645.328	AT5G28450 description:Chlorophyll A-B binding family protein
	BraA04g001160.4.1C.8	476.337	296.192	238.338	596.658	523.914	755.112	AT5G28450 description:Chlorophyll A-B binding family protein
BraA04g016490.4.1C	BraA04g016490.4.1C.1	38.693	24.807	18.533	70.204	54.308	68.263	TCH2 description:EF hand calcium-binding protein family
BraA05g011410.4.1C	BraA05g011410.4.1C.1	12.361	4.237	5.503	52.857	31.852	37.682	SPL3 description:squamosa promoter binding protein-like 3
	BraA06g041790.4.1C.1	26.364	29.241	29.433	24.07	20.223	23.615	CR88 description:Chaperone protein htpG family protein
	BraA06g041790.4.1C.2	26.876	29.809	30.005	24.537	20.615	24.073	CR88 description:Chaperone protein htpG family protein
BraA06g041790.4.1C	BraA06g041790.4.1C.4	31.538	32.633	33.371	28.721	24.222	28.41	CR88 description:Chaperone protein htpG family protein
	BraA06g041790.4.1C.5	26.465	29.353	29.546	24.162	20.3	23.705	CR88 description:Chaperone protein htpG family protein
	BraA06g041790.4.1C.9	27.865	30.992	31.367	25.805	21.298	25.105	CR88 description:Chaperone protein htpG family protein

	BraA06g041790.4.1C.16	29.595	31.094	32.085	27.062	22.831	26.66	CR88 description:Chaperone protein htpG family protein
	BraA06g041790.4.1C.12	24.82	32.82	32.077	19.125	18.045	21.038	CR88 description:Chaperone protein htpG family protein
	BraA06g041790.4.1C.21	26.398	29.279	29.471	24.1	20.249	23.645	CR88 description:Chaperone protein htpG family protein
	BraA06g041790.4.1C.22	27.157	30.121	30.318	24.793	20.831	24.325	CR88 description:Chaperone protein htpG family protein
	BraA06g041790.4.1C.24	18.242	28.063	29.665	11.799	12.254	14.131	CR88 description:Chaperone protein htpG family protein
	BraA06g041790.4.1C.28	26.431	29.316	29.508	24.131	20.275	23.675	CR88 description:Chaperone protein htpG family protein
	BraA06g041790.4.1C.29	28.458	37.106	28.019	22.712	21.243	23.195	CR88 description:Chaperone protein htpG family protein
	BraA06g041790.4.1C.30	19.932	29.196	31.127	15.585	15.429	15.303	CR88 description:Chaperone protein htpG family protein
	BraA06g041790.4.1C.31	31.538	32.633	33.371	28.721	24.222	28.41	CR88 description:Chaperone protein htpG family protein
BraA06g044230.4.1C	BraA06g044230.4.1C.1	44.006	30.966	19.891	48.944	54.75	59.705	CKB2 description:casein kinase II beta chain 2
BraA08g033050.4.1	BraA08g033050.4.1C.2	16.945	18.848	7.237	4.749	2.811	1.723	ARR3 description:response regulator 3
BraA10g033260.4.1C	BraA10g033260.4.1C.1	8.697	13.992	15.092	6.638	7.217	6.995	PRR7 description:pseudo-response regulator 7

Supplemental Table 6. Differentially expressed genes between GA_3-treated (GA^+) and untreated (GA^-) samples in 'CHOY' at seven days after GA_3 application.

Gene ID	Splicing variant for each gene	TPM values for each of the three biological replicates.						Annotation
		GA -			GA+			
		1	2	3	1	2	3	
BraA02g044150.4.1C	BraA02g044150.4.1C.1	12.238	14.251	12.234	3.655	6.783	3.364	PRR5 description:two-component response regulator-like protein
BraA02g047100.4.1C	BraA02g047100.4.1C.1	17.973	13.152	14.459	21.768	22.635	18.768	MAF1 description:K-box region and MADS-box transcription factor family protein
BraA03g006840.4.1C	BraA03g006840.4.1C.1	138.688	109.215	137.745	308.27	193.637	254.327	GASA4 description:GAST1 protein homolog 4
BraA03g018150.4.1C	BraA03g018150.4.1C.1	7.328	4.161	7.939	37.155	17.138	28.454	SPL3 description:squamosa promoter binding protein-like 3
BraA03g024180.4.1C	BraA03g024180.4.1C.1	29.093	12.678	30.892	48.176	57.486	48.186	AGL20 description:AGAMOUS-like 20
BraA03g039240.4.1C	BraA03g039240.4.1C.2	4.834	6.601	5.681	10.767	7.495	9.449	VRN1 description:AP2/B3-like transcriptional factor family protein
	BraA03g039240.4.1C.4	4.834	6.601	5.681	10.767	7.495	9.449	VRN1 description:AP2/B3-like transcriptional factor family protein
BraA03g045130.4.1C	BraA03g045130.4.1C.4	15.247	18.206	19.091	3.559	13.067	8.874	TOC1 description:CCT motif -containing response regulator protein

	BraA03g045130.4.1C.8	16.908	20.894	21.727	3.975	13.414	9.993	TOC1 description:CCT motif - containing response regulator protein
BraA03g062330.4.1C	BraA03g062330.4.1C.4	9.01	10.197	10.668	4.783	5.107	5.569	AP2 description:Integrase-type DNA-binding superfamily protein
BraA04g016490.4.1C	BraA04g016490.4.1C.1	62.226	33.336	44.407	90.433	111.163	73.388	TCH2 description:EF hand calcium-binding protein family
BraA04g018360.4.1C	BraA04g018360.4.1C.1	9.148	8.338	9.101	13.89	11.786	13.556	SVP description:K-box region and MADS-box transcription factor family protein
	BraA04g018360.4.1C.2	9.656	8.802	9.607	14.661	12.44	14.309	SVP description:K-box region and MADS-box transcription factor family protein
BraA05g007990.4.1C	BraA05g007990.4.1C.1	4.501	4.081	4.952	10.469	8.129	11.363	FHY1 description:far-red elongated hypocotyl 1
	BraA05g007990.4.1C.2	4.368	3.96	4.806	10.161	7.89	11.132	FHY1 description:far-red elongated hypocotyl 1
	BraA05g007990.4.1C.3	4.368	3.96	4.806	10.161	7.89	11.132	FHY1 description:far-red elongated hypocotyl 1
	BraA05g007990.4.1C.4	4.368	3.96	4.806	10.161	7.89	11.132	FHY1 description:far-red elongated hypocotyl 1
BraA05g011410.4.1C	BraA05g011410.4.1C.1	9.372	5.736	6.403	22.124	18.015	18.419	SPL3 description:squamosa promoter binding protein-like 3

	BraA06g034580.4.1C.1	28.858	32.937	37.898	8.848	22.25	13.234	PRR9 description:pseudo-response regulator 9
	BraA06g034580.4.1C.2	26.336	28.465	33.712	4.766	18.409	10.84	PRR9 description:pseudo-response regulator 9
	BraA06g034580.4.1C.3	29.028	28.118	37.14	3.046	18.87	11.093	PRR9 description:pseudo-response regulator 9
	BraA06g034580.4.1C.4	30.634	38.455	42.21	13.457	26.745	17.362	PRR5 description:two-component response regulator-like protein
BraA06g034580.4.1C	BraA06g034580.4.1C.5	30.625	37.272	41.687	12.941	26.24	17.009	PRR5 description:two-component response regulator-like protein
	BraA06g034580.4.1C.6	31.376	29.991	40.556	2.994	19.983	12.096	PRR9 description:pseudo-response regulator 9
	BraA06g034580.4.1C.7	30.634	38.455	42.21	13.457	26.745	17.362	PRR5 description:two-component response regulator-like protein
	BraA06g034580.4.1C.8	29.755	33.961	39.075	9.123	22.941	13.645	PRR9 description:pseudo-response regulator 9
BraA06g034940.4.1C	BraA06g034940.4.1C.1	6.69	4.46	8.03	15.562	12.879	14.819	FPF1 description:flowering promoting factor 1
BraA06g037110.4.1C	BraA06g037110.4.1C.1	7.286	6.259	8.467	17.616	20.759	12.463	LSH1 description:LIGHT-DEPENDENT SHORT HYPOCOTYLS-like protein (DUF640)

	BraA06g037110.4.1C.2	7.286	6.259	8.467	17.616	20.759	12.463	LSH1 description:LIGHT-DEPENDENT SHORT HYPOCOTYLS-like protein (DUF640)
	BraA07g030570.4.1C.3	6.419	5.075	8.008	10.967	8.494	9.396	TFIIIA description:transcription factor IIIA
BraA07g030570.4.1C	BraA07g030570.4.1C.4	6.419	5.075	8.008	10.967	8.494	9.396	TFIIIA description:transcription factor IIIA
	BraA07g030570.4.1C.5	6.419	5.075	8.008	10.967	8.494	9.396	TFIIIA description:transcription factor IIIA
BraA09g002740.4.1C	BraA09g002740.4.1C.2	12.843	11.461	6.242	14.934	19.678	19.419	DFL2 description:Auxin-responsive GH3 family protein
	BraA09g002740.4.1C.5	12.843	11.461	6.242	14.934	19.678	19.419	DFL2 description:Auxin-responsive GH3 family protein
	BraA09g007160.4.1C.1	17.935	19.882	17.734	10.624	16.51	12.114	TOC1 description:CCT motif -containing response regulator protein
BraA09g007160.4.1C	BraA09g007160.4.1C.3	18.036	20.124	17.707	9.527	16.045	11.684	TOC1 description:CCT motif -containing response regulator protein
	BraA09g007160.4.1C.4	19.652	21.489	19.851	12.48	18.461	14.028	TOC1 description:CCT motif -containing response regulator protein
BraA09g016210.4.1C	BraA09g016210.4.1C.6	29.073	34.108	24.275	6.72	18.803	19.132	SCO1 description:Translation elongation factor EFG/EF2 protein

	BraA09g016210.4.1C.11	32.613	41.381	29.19	12.527	21.484	23.225	SCO1 description:Translation elongation factor EFG/EF2 protein
	BraA09g016210.4.1C.15	25.302	31.59	23.127	3.027	15.105	15.734	SCO1 description:Translation elongation factor EFG/EF2 protein
	BraA09g016210.4.1C.14	26.227	33.637	24.457	8.056	17.422	18.554	SCO1 description:Translation elongation factor EFG/EF2 protein
	BraA09g016210.4.1C.16	20.719	26.461	18.3	2.446	12.377	14.091	SCO1 description:Translation elongation factor EFG/EF2 protein
BraA09g030800.4.1C	BraA09g030800.4.1C.8	11.054	10.758	8.253	1.354	7.626	2.838	CRY1 description:cryptochrome 1
	BraA10g002840.4.1C.1	10.643	12.553	15.234	8.24	9.51	8.095	CRY2 description:cryptochrome 2
	BraA10g002840.4.1C.2	10.643	12.553	15.234	8.24	9.51	8.095	CRY2 description:cryptochrome 2
	BraA10g002840.4.1C.3	10.643	12.553	15.234	8.24	9.51	8.095	CRY2 description:cryptochrome 2
	BraA10g002840.4.1C.7	10.662	12.575	15.261	8.254	9.527	8.109	CRY2 description:cryptochrome 2
	BraA10g002840.4.1C.9	10.662	12.575	15.261	8.254	9.527	8.109	CRY2 description:cryptochrome 2
BraA10g002840.4.1C	BraA10g002840.4.1C.10	14.078	16.409	19.052	11.964	12.698	10.704	CRY2 description:cryptochrome 2
	BraA10g002840.4.1C.11	7.567	8.368	10.636	2.188	4.474	4.733	CRY2 description:cryptochrome 2
	BraA10g002840.4.1C.12	10.291	12.12	14.324	4.179	8.886	6.658	CRY2 description:cryptochrome 2
	BraA10g002840.4.1C.13	10.267	12.092	14.291	4.169	8.865	6.643	CRY2 description:cryptochrome 2
	BraA10g002840.4.1C.14	10.662	12.575	15.261	8.254	9.527	8.109	CRY2 description:cryptochrome 2
	BraA10g002840.4.1C.15	11.571	12.748	16.413	7.07	9.936	8.217	CRY2 description:cryptochrome 2
BraA10g008030.4.1C	BraA10g008030.4.1C.2	13.296	13.67	11.051	3.21	6.5	8.843	CH1 description:Pheophorbide a oxygenase family protein with Rieske 2Fe-2S domain-containing protein

	BraA10g008030.4.1C.7	11.841	12.458	9.355	2.13	5.839	7.802	CH1 description:Pheophorbide a oxygenase family protein with Rieske 2Fe-2S domain-containing protein
BraA10g027210.4.1C	BraA10g027210.4.1C.1	7.47	5.161	2.588	10.801	8.45	13.828	HY5 description:Basic-leucine zipper (bZIP) transcription factor family protein
BraA10g028070.4.1C	BraA10g028070.4.1C.1	21.069	20.474	21.441	14.57	17.309	17.516	FLC description:K-box region and MADS-box transcription factor family protein

Supplemental Table 7. Differentially expressed genes between GA_3-treated (GA^+) and untreated (GA^-) samples in ‘Kohiki’ at seven days after GA_3 application.

Gene ID	Splicing variant for each gene	TPM values for each of the three biological replicates. GA -			GA+			Annotation
		1	2	3	1	2	3	
BraA01g000530.4.1C	BraA01g000530.4.1C.3	5.586	3.609	1.036	6.685	8.121	10.952	BRI1 description:Leucine-rich receptor-like protein kinase family protein
BraA01g018090.4.1C	BraA01g018090.4.1C.1	1.701	2.039	0.316	6.951	12.332	5.203	DFL1 description:Auxin-responsive GH3 family protein
BraA01g018260.4.1C	BraA01g018260.4.1C.1	9.74	8.103	9.635	11.476	12.029	12.618	CIP7 description:COP1-interacting protein 7
	BraA01g018260.4.1C.2	10.932	9.387	11.501	13.203	13.381	14.418	CIP7 description:COP1-interacting protein 7
	BraA01g018260.4.1C.3	10.932	9.387	11.501	13.203	13.381	14.418	CIP7 description:COP1-interacting protein 7
BraA01g033220.4.1C	BraA01g033220.4.1C.1	6.533	3.925	2.306	8.261	10.073	9.081	TIC description:time for coffee
	BraA01g033220.4.1C.2	6.533	3.925	2.306	8.261	10.073	9.081	TIC description:time for coffee
	BraA01g033220.4.1C.6	6.583	4.067	0.981	8.493	10.088	9.599	TIC description:time for coffee
	BraA01g033220.4.1C.9	6.583	4.067	0.981	8.493	10.088	9.599	TIC description:time for coffee
	BraA01g033220.4.1C.12	6.821	3.983	2.508	8.135	10.431	8.901	TIC description:time for coffee

	BraA01g033220.4.1C.13	6.607	4.332	1.175	9.358	10.587	9.961	TIC description:time for coffee
	BraA01g033220.4.1C.14	6.583	4.067	0.981	8.493	10.088	9.599	TIC description:time for coffee
	BraA01g033220.4.1C.17	6.696	4.024	2.009	7.95	10.606	9.353	TIC description:time for coffee
	BraA01g033220.4.1C.18	6.533	3.925	2.306	8.261	10.073	9.081	TIC description:time for coffee
	BraA01g033220.4.1C.19	6.585	3.957	2.314	8.328	10.154	9.154	TIC description:time for coffee
	BraA01g033220.4.1C.22	6.098	3.897	1.587	8.154	10.032	8.94	TIC description:time for coffee
BraA02g003400.4.1C	BraA02g003400.4.1C.1	36.98	35.476	47.804	26.73	25.626	30.754	MAF4 description:K-box region and MADS-box transcription factor family protein
BraA02g034910.4.1C	BraA02g034910.4.1C.1	8.579	5.438	1.072	10.395	11.982	12.176	LBA1 description:RNA helicase
	BraA02g034910.4.1C.2	8.278	5.461	0.704	10.024	11.812	11.884	LBA1 description:RNA helicase
	BraA02g034910.4.1C.3	8.579	5.438	1.072	10.395	11.982	12.176	LBA1 description:RNA helicase
	BraA02g034910.4.1C.4	10.743	6.823	1.775	12.636	14.77	14.414	LBA1 description:RNA helicase
BraA02g041330.4.1C	BraA02g041330.4.1C.1	6.109	7.773	6.823	9.242	11.297	9.088	PAT1 description:GRAS family transcription factor
	BraA02g041330.4.1C.2	6.109	7.773	6.823	9.242	11.297	9.088	PAT1 description:GRAS family transcription factor
	BraA02g041330.4.1C.3	6.109	7.773	6.823	9.242	11.297	9.088	PAT1 description:GRAS family transcription factor
	BraA02g041330.4.1C.4	6.109	7.773	6.823	9.242	11.297	9.088	PAT1 description:GRAS family transcription factor

	BraA02g041330.4.1C.6	6.501	8.271	7.26	9.834	12.021	9.671	PAT1 description:GRAS family transcription factor
BraA03g006650.4.1C	BraA03g006650.4.1C.1	39.074	32.079	27.045	69.175	123.452	179.263	AT5G14920 description:Gibberellin-regulated family protein
BraA03g018150.4.1C	BraA03g018150.4.1C.1	1.259	0.993	5.041	10.34	13.65	11.75	SPL3 description:squamosa promoter binding protein-like 3
BraA03g020990.4.1C	BraA03g020990.4.1C.1	8.119	3.407	0.632	8.586	14.254	13.527	SYD description:P-loop containing nucleoside triphosphate hydrolases superfamily protein
BraA03g022850.4.1C	BraA03g022850.4.1C.1	4.661	5.916	1.758	8.69	9.16	12.094	PIL5 description:phytochrome interacting factor 3-like 5
	BraA03g022850.4.1C.2	4.455	5.465	1.301	7.221	8.456	11.775	PIL5 description:phytochrome interacting factor 3-like 5
	BraA03g022850.4.1C.3	4.181	5.941	1.843	8.802	8.965	12.044	PIL5 description:phytochrome interacting factor 3-like 5
	BraA03g022850.4.1C.4	4.661	5.916	1.758	8.69	9.16	12.094	PIL5 description:phytochrome interacting factor 3-like 5
BraA03g028490.4.1C	BraA03g028490.4.1C.1	11.775	12.597	19.948	59.765	88.638	85.985	EARLI1 description:Bifunctional inhibitor/lipid-transfer protein/seed storage 2S albumin superfamily protein
BraA03g040950.4.1C	BraA03g040950.4.1C.3	8.13	5.052	1.552	8.708	13.732	14.372	TIC description:time for coffee
	BraA03g040950.4.1C.4	7.419	4.258	1.061	7.958	12.67	12.855	TIC description:time for coffee

	BraA03g055960.4.1C.2	7.971	10.91	9.299	13.594	12.193	11.386	CKL13 description:casein kinase like 13
BraA03g055960.4.1C	BraA03g055960.4.1C.3	7.971	10.91	9.299	13.594	12.193	11.386	CKL13 description:casein kinase like 13
	BraA03g055960.4.1C.6	7.971	10.91	9.299	13.594	12.193	11.386	CKL13 description:casein kinase like 13
BraA03g057890.4.1C	BraA03g057890.4.1C.2	8.78	11.267	9.569	12.553	14.101	13.438	SKB1 description:SHK1 binding protein 1
	BraA04g001160.4.1C.1	236.583	286.266	265.007	176.513	161.742	120.297	AT5G28450 description:Chlorophyll A-B binding family protein
	BraA04g001160.4.1C.2	259.709	325.849	345.632	184.108	176.676	133.593	AT5G28450 description:Chlorophyll A-B binding family protein
	BraA04g001160.4.1C.3	296.13	368.117	398.142	209.313	200.291	151.18	AT5G28450 description:Chlorophyll A-B binding family protein
BraA04g001160.4.1C	BraA04g001160.4.1C.5	296.13	368.117	398.142	209.313	200.291	151.18	AT5G28450 description:Chlorophyll A-B binding family protein
	BraA04g001160.4.1C.4	259.709	325.849	345.632	184.108	176.676	133.593	AT5G28450 description:Chlorophyll A-B binding family protein
	BraA04g001160.4.1C.8	296.13	368.117	398.142	209.313	200.291	151.18	AT5G28450 description:Chlorophyll A-B binding family protein
	BraA04g001160.4.1C.7	217.304	264.726	240.306	162.62	149.237	111.127	AT5G28450 description:Chlorophyll A-B binding family protein
BraA05g026910.4.1C	BraA05g026910.4.1C.1	4.582	3.425	1.906	7.291	11.136	10.829	TIC description:time for coffee

BraA06g037110.4.1C	BraA06g037110.4.1C.1	6.306	5.502	8.216	18.432	15.486	20.589	LSH1 description:LIGHT-DEPENDENT SHORT HYPOCOTYLS-like protein (DUF640)
	BraA06g037110.4.1C.2	6.306	5.502	8.216	18.432	15.486	20.589	LSH1 description:LIGHT-DEPENDENT SHORT HYPOCOTYLS-like protein (DUF640)
BraA06g043330.4.1C	BraA06g043330.4.1C.1	13.812	13.518	5.976	27.487	19.078	20.675	GAI description:GRAS family transcription factor family protein
	BraA06g043330.4.1C.2	13.812	13.518	5.976	27.487	19.078	20.675	GAI description:GRAS family transcription factor family protein
BraA06g047220.4.1C	BraA06g047220.4.1C.1	7.827	5.834	3.588	11.067	16.667	15.299	BRI1 description:Leucine-rich receptor-like protein kinase family protein
BraA07g025700.4.1C	BraA07g025700.4.1C.1	134.088	162.828	194.49	96.116	85.317	69.751	AT5G28450 description:Chlorophyll A-B binding family protein
	BraA07g025700.4.1C.2	134.088	162.828	194.49	96.116	85.317	69.751	AT5G28450 description:Chlorophyll A-B binding family protein
	BraA07g025700.4.1C.3	150.496	190.95	246.698	110.051	97.446	76.924	AT5G28450 description:Chlorophyll A-B binding family protein
	BraA07g025700.4.1C.4	154.549	195.56	258.181	113.56	99.21	79.295	AT5G28450 description:Chlorophyll A-B binding family protein

	BraA07g025700.4.1C.5	131.471	159.961	187.142	93.847	84.236	68.193	AT5G28450 description:Chlorophyll A-B binding family protein
	BraA07g025700.4.1C.6	150.496	190.95	246.698	110.051	97.446	76.924	AT5G28450 description:Chlorophyll A-B binding family protein
	BraA07g025700.4.1C.7	162.904	205.224	285.697	115.474	104.465	81.719	AT5G28450 description:Chlorophyll A-B binding family protein
BraA07g043390.4.1C	BraA07g043390.4.1C.1	6.225	3.307	1.763	7.128	10.865	9.207	NUA description:nuclear pore anchor
	BraA07g043390.4.1C.2	7.027	3.809	2.133	7.901	12.266	10.337	NUA description:nuclear pore anchor
	BraA07g043390.4.1C.3	6.86	3.719	2.082	7.713	11.974	10.091	NUA description:nuclear pore anchor
	BraA07g043390.4.1C.4	8.544	4.906	3.039	9.36	14.173	11.202	NUA description:nuclear pore anchor
	BraA07g043390.4.1C.7	8.544	4.906	3.039	9.36	14.173	11.202	NUA description:nuclear pore anchor
	BraA07g043390.4.1C.5	6.859	3.794	2.127	7.835	12.125	10.06	NUA description:nuclear pore anchor
	BraA07g043390.4.1C.8	6.86	3.719	2.082	7.713	11.974	10.091	NUA description:nuclear pore anchor
	BraA07g043390.4.1C.9	6.86	3.719	2.082	7.713	11.974	10.091	NUA description:nuclear pore anchor
	BraA07g043390.4.1C.10	6.236	3.314	1.766	7.142	10.886	9.225	NUA description:nuclear pore anchor
	BraA07g043390.4.1C.11	6.545	3.574	1.263	7.603	11.661	9.773	NUA description:nuclear pore anchor
	BraA07g043390.4.1C.12	5.916	3.162	1.061	7.003	10.539	8.873	NUA description:nuclear pore anchor
	BraA07g043390.4.1C.13	5.949	3.18	1.067	7.042	10.599	8.923	NUA description:nuclear pore anchor
BraA08g009400.4.1C	BraA08g009400.4.1C.10	6.407	3.512	5.207	7.794	9.944	10.04	HAF2 description:histone acetyltransferase of the TAFII250 family 2

	BraA08g009400.4.1C.11	6.608	3.623	5.303	8.039	10.256	10.355	HAF2 description:histone acetyltransferase of the TAFII250 family 2
	BraA08g009400.4.1C.19	6.42	3.519	5.152	7.81	9.964	10.06	HAF2 description:histone acetyltransferase of the TAFII250 family 2
	BraA08g009400.4.1C.30	6.387	3.443	5.069	7.834	9.815	10.018	HAF2 description:histone acetyltransferase of the TAFII250 family 2
	BraA08g009400.4.1C.35	7.941	4.254	7.857	9.824	11.511	12.736	HAF2 description:histone acetyltransferase of the TAFII250 family 2
BraA08g023180.4.1	BraA08g023180.4.1C.1	11.068	7.303	2.382	16.182	19.943	21.989	BRI1 description:Leucine-rich receptor-like protein kinase family protein
	BraA08g023180.4.1C.2	11.068	7.303	2.382	16.182	19.943	21.989	BRI1 description:Leucine-rich receptor-like protein kinase family protein
BraA09g007160.4.1C	BraA09g007160.4.1C.3	20.758	16.575	17.652	21.398	24.71	23.327	TOC1 description:CCT motif -containing response regulator protein
BraA09g016210.4.1C	BraA09g016210.4.1C.1	23.615	23.001	20.351	19.425	14.921	11.297	SCO1 description:Translation elongation factor EFG/EF2 protein

BraA09g016210.4.1C.2	24.081	23.875	22.838	19.51	14.93	11.009	SCO1 description:Translation elongation factor EFG/EF2 protein
BraA09g016210.4.1C.3	24.336	23.703	20.973	20.019	15.376	11.642	SCO1 description:Translation elongation factor EFG/EF2 protein
BraA09g016210.4.1C.4	24.081	23.875	22.838	19.51	14.93	11.009	SCO1 description:Translation elongation factor EFG/EF2 protein
BraA09g016210.4.1C.10	24.532	23.893	21.141	20.179	15.5	11.736	SCO1 description:Translation elongation factor EFG/EF2 protein
BraA09g016210.4.1C.7	33.076	35.931	48.598	27.543	20.38	14.519	SCO1 description:Translation elongation factor EFG/EF2 protein
BraA09g016210.4.1C.8	26.113	26.454	29.238	21.379	16.34	11.661	SCO1 description:Translation elongation factor EFG/EF2 protein
BraA09g016210.4.1C.9	24.899	25	22.011	20.099	14.801	11.943	SCO1 description:Translation elongation factor EFG/EF2 protein
BraA09g016210.4.1C.12	25.035	24.383	21.575	20.593	15.817	11.976	SCO1 description:Translation elongation factor EFG/EF2 protein
BraA09g016210.4.1C.17	36.566	37.577	44.801	31.028	24.41	17.318	SCO1 description:Translation elongation factor EFG/EF2 protein
BraA09g016210.4.1C.18	28.892	31.387	42.452	24.059	17.803	12.683	SCO1 description:Translation elongation factor EFG/EF2 protein
BraA09g016210.4.1C.20	24.049	23.423	20.725	19.782	15.195	11.505	SCO1 description:Translation elongation factor EFG/EF2 protein

	BraA09g016210.4.1C.19	23.986	23.362	20.671	19.73	15.155	11.475	SCO1 description:Translation elongation factor EFG/EF2 protein
BraA09g024210.4.1C	BraA09g024210.4.1C.1	25.54	21.264	16.376	45.639	39.103	42.09	GAI description:GRAS family transcription factor family protein
BraA09g027750.4.1C	BraA09g027750.4.1C.1	6.615	6.433	7.103	10.098	9.188	10.691	AGL14 description:AGAMOUS-like 14
BraA09g043240.4.1C	BraA09g043240.4.1C.1	18.529	11.882	2.719	21.556	26.166	26.607	GI description:gigantea protein (GI)
	BraA09g043240.4.1C.2	18.111	11.614	2.658	21.071	25.577	26.008	GI description:gigantea protein (GI)
	BraA09g043240.4.1C.3	18.529	11.882	2.719	21.556	26.166	26.607	GI description:gigantea protein (GI)
	BraA09g043240.4.1C.4	18.529	11.882	2.719	21.556	26.166	26.607	GI description:gigantea protein (GI)
